# Large‐scale analysis of *Drosophila* core promoter function using synthetic promoters

**DOI:** 10.15252/msb.20209816

**Published:** 2022-02-14

**Authors:** Zhan Qi, Christophe Jung, Peter Bandilla, Claudia Ludwig, Mark Heron, Anja Sophie Kiesel, Mariam Museridze, Julia Philippou‐Massier, Miroslav Nikolov, Alessio Renna Max Schnepf, Ulrich Unnerstall, Stefano Ceolin, Bettina Mühlig, Nicolas Gompel, Johannes Soeding, Ulrike Gaul

**Affiliations:** ^1^ Department of Biochemistry, Gene Center Ludwig‐Maximillians‐Universität München Feodor‐Lynen‐str 25 Munich Germany; ^2^ Department of Biology II, Evolutionary Biology Ludwig‐Maximilians‐Universität München Planegg‐Martinsried Germany; ^3^ Max Planck Institute for Biophysical Chemistry Göttingen Germany

**Keywords:** gene expression, modeling, motif search, mutational analysis, promoter, Biotechnology & Synthetic Biology, Chromatin, Transcription & Genomics

## Abstract

The core promoter plays a central role in setting metazoan gene expression levels, but how exactly it “computes” expression remains poorly understood. To dissect its function, we carried out a comprehensive structure–function analysis in *Drosophila*. First, we performed a genome‐wide bioinformatic analysis, providing an improved picture of the sequence motifs architecture. We then measured synthetic promoters’ activities of ~3,000 mutational variants with and without an external stimulus (hormonal activation), at large scale and with high accuracy using robotics and a dual luciferase reporter assay. We observed a strong impact on activity of the different types of mutations, including knockout of individual sequence motifs and motif combinations, variations of motif strength, nucleosome positioning, and flanking sequences. A linear combination of the individual motif features largely accounts for the combinatorial effects on core promoter activity. These findings shed new light on the quantitative assessment of gene expression in metazoans.

## Graphical abstract

A large‐scale structure‐function analysis of ~3,000 synthetic promoters reveals the sequence determinants of *Drosophila* core promoters.

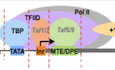

## Introduction

Appropriate gene expression with the correct timing is crucial for the development and diversity of all organisms. The control of gene expression occurs primarily at the process of transcription (Levine & Tjian, 2003), and the core promoter—the region immediately surrounding the transcription start site (TSS)—makes an essential contribution for setting the gene expression level (Lubliner *et al*, 2015).

The RNA polymerase II (Pol II) core promoter is the minimal DNA sequence that is recognized by the basal transcription machinery (Smale & Kadonaga, 2003; Thomas & Chiang, 2006; Juven‐Gershon *et al*, 2008). It comprises the TSS and approximately 150 bp of the flanking sequence. The accurate transcription initiation and basal expression level of a gene are primarily determined by differential recruitment of the transcription machinery, consisting of Pol II and general transcription factors (GTFs), to its core promoter region (Smale & Kadonaga, 2003; Thomas & Chiang, 2006; Juven‐Gershon *et al*, 2008; Lagha *et al*, 2013; Pimmett *et al*, 2021). Genome‐wide studies have revealed various properties of native core promoters. In particular, sequence motifs that are over‐represented around TSSs mostly mark the potential binding sites of GTFs or other transcription factors (TFs) (Burke & Kadonaga, 1997; FitzGerald *et al*, 2006; Ohler, 2006; Parry *et al*, 2010). A number of core promoter elements (CPE) have been described in eukaryotic core promoters, such as the TATA box, the initiator (Inr), or the downstream promoter element (DPE). These elements however typically only occur in a fraction of promoters, prompting the question of how the transcription machinery finds the core promoter in the absence of such motifs. Yet unknown motifs or the incorporation of physical properties of the DNA within the core promoter region may contribute to an explanation. Moreover, genetic variations occurring at the motif sites alter both promoter strength and TSS position significantly (Schor *et al*, 2017). Although the genomic analysis of native sequences suggests certain causal relationships, the variations in genomic sequences have been very challenging to predict (Seizl *et al*, 2011). This makes it difficult to uncover the sequence attributes responsible for activity changes. Noteworthy, Arnold *et al* (2017) showed for the main motifs Inr, TATA, and DPE that the resemblance with the canonical sequences correlates with the responsiveness of the enhancer targeting the promoter (i.e., how much expression changes when an enhancer is active), with an increasing responsiveness observed for higher position weight matrix (PWM) match scores. Interestingly, they also found that the correlation is higher for strongly responsive sequences than for weaker ones. However, it remains difficult to ascertain the influence of specific features except by directly altering them and measuring the effect on expression levels.

Facilitated by DNA synthesis technology and next‐generation sequencing, high‐throughput approaches such as massively parallel reporter assays (MPRAs) have been developed to test how the DNA sequence affects gene expression (transcripts) at single molecule resolution and at large scale (Patwardhan *et al*, 2009; Melnikov *et al*, 2012; Sharon *et al*, 2012; Arnold *et al*, 2013). A second kind of MPRA method quantifies the protein fluorescence as the readout of reporter gene expression but can only obtain discrete expression measurements because of their “bin” sorting design (which cannot sense subtle effects) and of the intrinsically relatively narrow dynamical range of the fluorescence readout (Lubliner *et al*, 2015). Moreover, most of these studies focused on enhancers, especially on single TF binding sites. Only few MPRAs were designed for *in vivo* promoter analysis, such as the extensive studies on fully designed yeast proximal promoter regions (Sharon *et al*, 2012) and yeast core promoter sequences (Lubliner *et al*, 2015), or the analysis of autonomous promoter activity of random genome fragments in humans (Van Arensbergen *et al*, 2017), and in *Drosophila melanogaster* (*D. melanogaster*) (Arnold *et al*, 2017). Thus, despite the pivotal role of core promoters in transcription initiation, it remains poorly understood how the components and sequence features of the core promoter determine expression levels.

**Figure 1:**
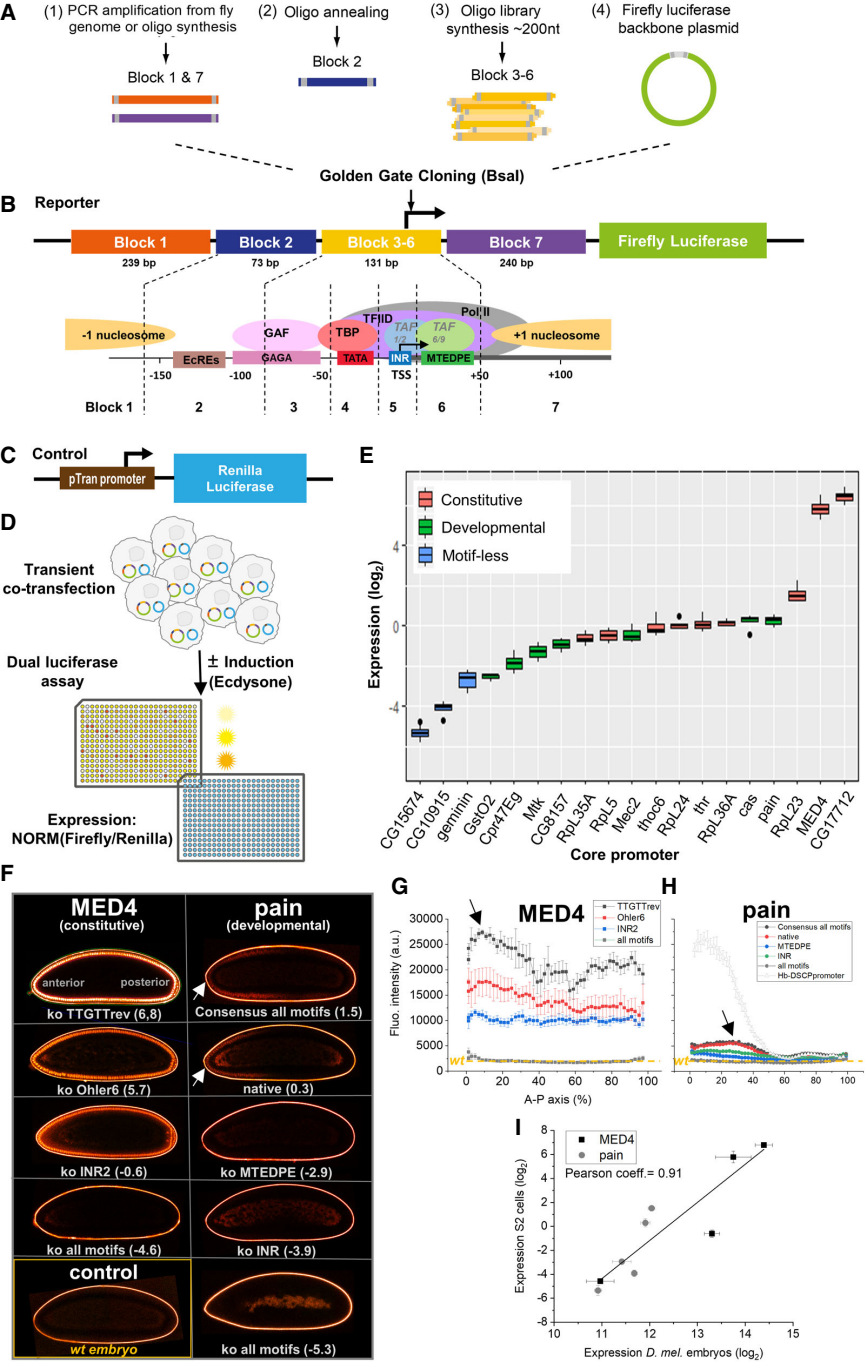
Experimental workflow and assay reproducibility AThe promoter region was divided into 7 building blocks: *block 1* with 239 bp of a potential −1 nucleosomal sequence; *block 2* with 73 bp sequence representing the ecdysone receptor binding region; *block 3‐6* with 131 bp sequence representing the native and perturbative core promoter regions from different architectures; *block 7* with 240 bp of a potential +1 nucleosomal sequence.BSynthetic promoter design—building blocks. The promoter region (sketch in lower panel) was divided into 7 building blocks: *block 1* with 239 bp sequence representing a potential −1 nucleosome; *block 2* with 73 bp sequence representing the ecdysone receptor binding region; *block 3‐6* with 131 bp sequence representing the native and perturbative core promoter regions from different architectures; *block 7* with 240 bp sequence representing a potential +1 nucleosome.CControl co‐transfected vector (backbone not represented) used for data normalization (Material and Methods), and consisting in a pTran promoter driving the expression of the Renilla Luciferase gene.DSimplified dual luciferase assay experimental workflow. To measure promoter activity quantitatively on a large scale with high reproducibility, we integrated the golden gate cloning strategy (BsaI cloning) with a high‐throughput experimental pipeline using automated robot systems for colony picking, reporter plasmids isolation, transient co‐transfection and dual luciferase assay (Fig EV1 and Materials and Methods for details).ENormalized expression levels of the native core promoters. Their activities spanned over a broad range of three orders of magnitude (promoter constructs contained *block 1.11* and *block 7.11* combination as nucleosomal sequences). Each color represents a different class of core promoter architecture. The middle hinge represents the median. The interquartile range the difference between the 75^th^ and 25^th^ percentiles. Individual points represent values over 1.5 times the interquartile range. 3‐4 biological replicate measurements (including new cell transfection procedures and measurements of promoter activities).FConfocal fluorescence sections of living *D. melanogaster* embryos (after ~40min at *stage5* during embryonic development) expressing an optimized reporter *mNeonGreen* protein (Ceolin *et al*, 2020), and carrying *hunchback anterior enhancer—tested core promoter—mNeon*. The fluorescence signal of *mNeonGreen* can be seen (in false colors) in the nuclei at the embryo peripheries. The promoters tested correspond to motif knockouts or motif substitution with consensus sequences from the constitutive MED4 (on the left) and the developmental *pain* (on the right) promoters. The ko motifs or the type of mutations are indicated in white, together with the normalized expression levels (in bracket) measured with our luciferase assay pipeline. Whereas MED4 promoters drive strong expression along the entire anterior (A)—posterior (P) axis of the embryo, *pain* embryos show weaker expression, consistently with the expression levels measured in the luciferase assay. Noteworthy, in contrast to the homogenous AP expression with the constitutive MED4 gene, the A‐P expressions patterns for developmental pain resemble the known AP gradient of expression typically observed for the Hb enhancer. The white arrows indicate the fluorescence signals of the nuclei in the anterior part of the embryos.G, HQuantification of the expression patterns in developing embryos, projected along the A‐P axis for MED4 (G) and for pain (H) for the different promoter variants, respectively. The errors bars are standard deviations from 3 to 4 biological replicates measurements (different embryos). The fluorescence background measured in a wild‐type embryo is shown as yellow dotted lines. The fluorescence patterns for pain recapitulate the typical *hb_ant* enhancer activity, characterized by a gradient of reporter expression (black arrow in H) with a sharp drop at around A‐P = 50%, which was expected for a developmental gene. An exemplary AP profile for the *hb_ant* enhancer is shown as gray empty triangles (the background was adjusted at about 3,000 a.u for better comparison). In contrast, the constructs containing the constitutive MED4 gene promoter lead to a stronger and more homogeneous expression with an only slightly enhanced expression level at the anterior tip (black arrow in G).IScatter plot of expression levels obtained in *D. melanogaster* S2 cells by our luciferase assay pipeline versus *mNeonGreen* reporter expression in living *D. melanogaster* embryos, revealing a high correlation (Pearson coefficient 0.91) between the two datasets. Error bars represent standard deviations of 3–4 biological replicate measurements.

**Figure EV1:**
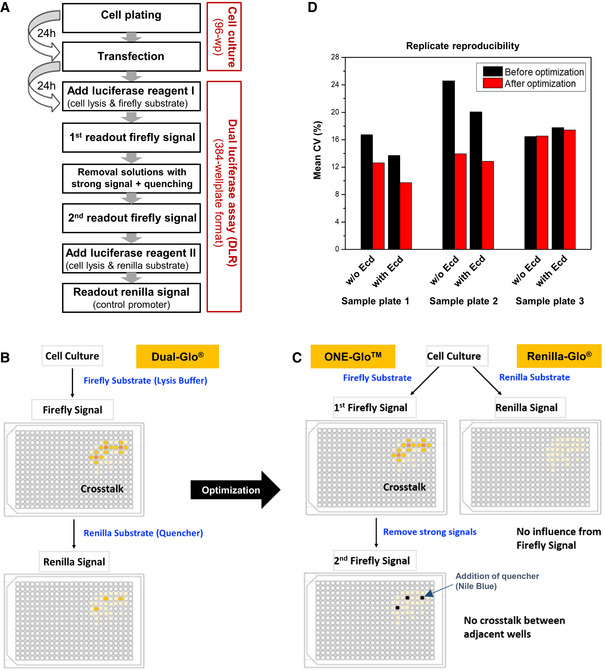
Assay development and reproducibility AWorkflow for automated transfection in 96‐well plate format, followed by dual luciferase (DLR) assay in 384‐well plate format (Materials and Methods). The transfection, lysis, and cell detachment occur in four cell culture 96‐wellplates, followed by their splitting into two 384‐well plates for separate readout of the Firefly and Renilla luminescence signals. This enabled to gain 4‐fold higher throughput and to save 2/3 of the luciferase assay reagent.B, CExperimental strategy to eliminate *crosstalk* artifacts. Separating Firefly and Renilla readouts (the two upper panels) avoids potential *crosstalk* between the Firefly and Renilla luminescence light within the same well. A second readout of the Firefly signal after removal of the solution from wells with very strong signal eliminates the *crosstalk* between neighboring wells.DComparison of expression level measurements for three 96‐well plates containing the same promoter construct samples measured on different days with and without ecdysone induction. Standard normalization (before optimization) uses only the ratios of Firefly and Renilla signals. After optimization of data normalization procedure of the luciferase assay readout (Materials and Methods), the mean reproducibility of the measurements improved from a mean coefficient of variation ~13% after optimizations, versus ~18% before.

This study aims to dissect the core promoter comprehensively and to elucidate the sequence determinants of promoters in *D. melanogaster S2* cells. We first questioned the motif architecture of *D. melanogaster* core promoters by developing a statistical framework based on PWMs to compute the over‐representation of candidate motifs in promoter sequences. Using the state‐of‐the‐art motif finding tool *XXmotif* algorithm (Luehr *et al*, 2012) leads to the *de novo* detection of all currently known, but also of several previously unknown motifs that are conserved and enriched in promoter regions. *Drosophila melanogaster* core promoters cluster into four classes characterized by distinct motif architectures and other promoter attributes. We then tested promoter activity using a dual luciferase assay, which is highly sensitive with a linear and broad dynamical range. We have integrated the entire experimental pipeline using automated robotic systems, including cloning and luciferase gene expression readout (Figs 1 and EV1). By extensively measuring the activity of mutagenized core promoter sequences for 19 representative genes, we corroborate the functional specificity of sequence motifs. We demonstrate that their strength, as measured by the position weight matrix (PWM) score, and their precise positioning are essential features determining core promoter activity. Additionally, we comprehensively mutagenized core promoter motifs using single base‐pair mutations to produce expression‐based position probability matrices (PPMs). Combinatorial motif mutations that alter both the strength and the positioning of all motifs often result in strong effects on activity, which are compared with the effects of individual motif mutations: We found that a linear combination of these individual motif features can largely account for the joint effects on core promoter activity. In addition, we investigated the influence of surrounding regions on promoter activity. By testing sequences impacting −1 and +1 nucleosomes, we show that their influence on the constitutive core promoter activity is relatively mild, the effect being stronger for nucleosome positioning sequences downstream of the TSS. We also tested the influence of context sequences (i.e., the background sequences surrounding the CPEs) and confirm their strong impact on expression. Finally, we investigated the response upon activation through an external hormonal stimulus by the steroid hormone ecdysone (a transcriptional activator). This hormone is important for metamorphosis, molting, and development of the eye and the nervous system in insects (Yamanaka *et al*, 2013). Its active form (20‐hydroxyecdysone) constitutes, together with its receptor (the ecdysone receptor EcR), a well‐studied activator system for gene expression. We found that the responsiveness of a given promoter depends on its architecture. Notably, ecdysone can induce both developmental and constitutive core promoters but the induction is stronger with the developmental ones. We also found a negative correlation between the ecdysone inducibility and the basal expression level; this correlation is more significant for constitutive promoters.

## Results

**Figure EV2:**
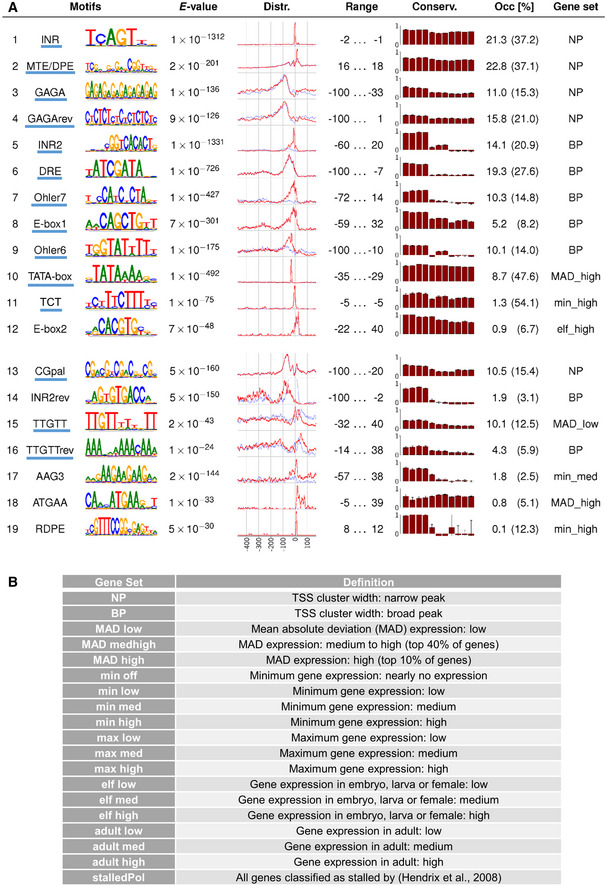
Core promoter motifs detected by *XXmotif* and subsequent analysis to validate the motifs Core promoter motifs of *D. melanogaster* we detected using *XXmotif*. Motifs underlined in blue are the ones used in our experimental pipeline. The first twelve motifs were previously described in literature, while the seven below the gap are novel. Column “*E‐values*” indicates the significance level obtained with *XXmotif*. Column “*Distr*” depicts a smoothed distribution (over five nucleotides) of all identified binding sites within the gene set having the highest mutual information (and positive correlation), indicated in column *“Gene set*” (details in B and in Appendix Table S2). *XXmotif* reports the region (relative to the defined TSS) with the highest enrichment of binding sites (Column “*Range*”). Column “*Conserv*.” indicates the average conservation of binding sites, where 1 represents a perfect conservation, and 0 the background conservation. The bars correspond to 11 related *Drosophila* species, ordered by ascending evolutionary distance (details in Appendix Fig S1). The novel motifs are highly conserved. Column “*Occ [%]*” gives the frequency of motif sites within the whole sequence set (the gene set of highest mutual information).Legend for the abbreviations used in the column “*Gene Set*” and in Fig EV3.

To select the genes to be tested, we first determined the *D. melanogaster* core promoter architecture by performing a bioinformatic analysis based on experimentally derived features, including expression strengths and variation during developmental stages (Graveley *et al*, 2011), Pol II stalling (Zeitlinger *et al*, 2007; Hendrix *et al*, 2008), TSSs mapping from CAGE data (Ni *et al*, 2010; Hoskins *et al*, 2011), and motif composition. To this end, we first applied the *XXmotif* algorithm presented previously (Luehr *et al*, 2012) for a genome‐wide motif search in annotated core promoter regions. *XXmotif* combines a *P*‐value that evaluates from its PWM whether the motif sites are located non‐randomly with respect to the TSS with motif over‐representation and conservation *P*‐values. Hence, this *de novo* motif analysis can be performed in a single run on large regions of the core promoter without losing the descriptive power of a PWM. Our analysis identified widely known motifs as well as some novel motif candidates with optimized PWMs based on enrichment, localization and conservation (Fig EV2 and Appendix Table S1). All identified CPEs are highly significant with E‐values ranging from 7 × 10^−48^ to 1 × 10^−1,331^ for known motifs, and 1 × 10^−24^ to 5 × 10^−160^ for the novel motifs. The already known motifs include INR, MTE/DPE (an overlapping version of the two previously identified motifs MTE and DPE, hereafter referred to as MTEDPE), GAGA, GAGArev, INR2 (also known as motif 1 or Ohler1), DRE, Ohler7, E‐Box1, Ohler6, TATA‐Box, TCT, and E‐Box2 (Fig EV2); we named the new motifs CGpal, INR2rev, TTGTT, TTGTTrev, AAG3, ATGAA, and RDPE (ribosomal downstream promoter element). The INR and two other previously described motifs, INR2 and TCT, are precisely positioned at the TSS, the TCT motif often co‐occurring with TATA‐Box. In contrast to Ohler (2006), we only identify a DPE motif that overlaps the adjacent MTE, but no separate MTE motif. Moreover, we identify two motifs (E‐box1 and E‐box2) containing the known E‐box consensus CANNTG that is bound by basic helix–loop–helix leucine zipper (bHLH‐zip) transcription factors. E‐box1 consists of the CAGCTG consensus and was computationally identified by FitzGerald *et al* (2006). E‐box2 consists of the CACGTG consensus; it is positioned downstream with respect to the TSS and bound by Myc‐Max complexes that activate the transcription of nearby genes (Walhout *et al*, 1997). We validated the novel (and the known) CPEs by examining the conservation of binding sites (passing the minimal score threshold and enriched region filter) between *Drosophila* species (Fig EV2A and Appendix Fig S1). Since sequence conservation is an inadequate measure for the conservation of CPEs (it ignores the PWM), we analyzed the difference of PWM scores between *D. melanogaster* and 11 related species (Appendix Fig S1). Three types of conservation can be distinguished: first, CPEs that are conserved in all *Drosophila* species (i.e., INR, MTE/DPE, TATA‐box, INR3, CACGTG, ATGAA, E‐box2); second, CPEs that are only conserved in the *melanogaster* subgroup—the four leftmost plotted species that are the closest related (Fig EV2A and Appendix Fig S1)—(i. e., INR2, DRE, Ohler7, Ohler6, revINR2, AAG3, RDPE); and third, CPEs that are well conserved within the *melanogaster* species and moderately conserved within the whole *Drosophila* genus (i.e., GAGA, revGAGA, E‐box1, CGpal, TTGTT, revTTGTT). Thus, the new motifs we discovered are well conserved among multiple *Drosophila* species.

**Figure EV3:**
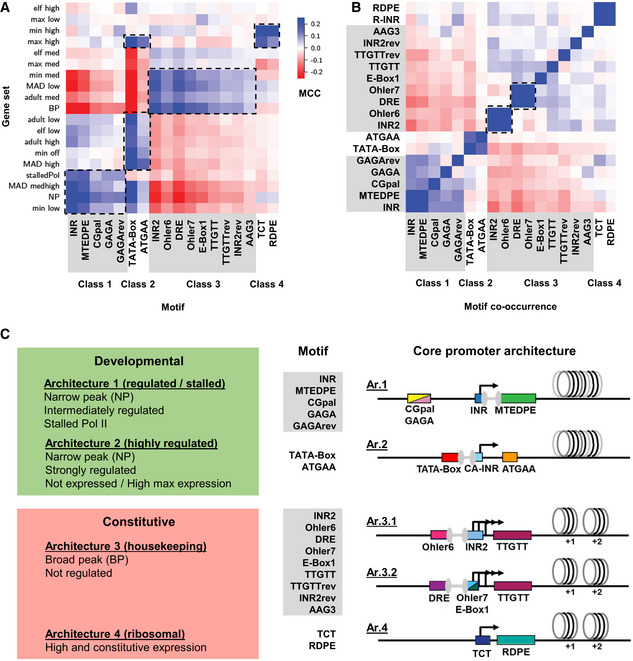
Genome‐wide analysis of promoter features Correlation of core promoter motifs with different features (motifs and meaning of the different features listed in Fig EV2B) reveals four distinct motif classes: class 1 motifs enriched in the gene sets of stalledPol, MAD medhigh, NP, and min low; class 2 motifs enriched in the gene sets of max high, adult low, elf low, adult high, min off and MAD high; class 3 motifs enriched in the gene sets of min med, MAD low, adult med and BP; class 4 motifs enriched in the gene sets of min high and max high (details on the motifs in Fig EV2A and on the different gene sets in Appendix Table S1). MCC: Matthews correlation coefficient. Groups of core promoter motifs that correlate strongly positively with particular features are highlighted with black dashed boxes.Core promoter elements co‐occur in architectures. Correlation of all core promoter elements to each other reveals elements that occur preferentially within the same promoter (positive correlation, blue, examples highlighted with black dashed boxes) or avoid each other (red). With the exception of the housekeeping class (class3)—which consists of two architectures (see C)—each promoter classes matches one architecture. In agreement with the four identified classes, most CPEs are positively correlated to all elements within their class and negatively correlated to CPEs belonging to other classes. Only the Class 4 elements are positively correlated to some motifs of especially Class 3. Negative correlations between elements within the same class are only found for elements located on both strands (e.g., GAGA versus revGAGA, TTGTT versus revTTGTT) and for two groups of elements within Class 3 (highlighted with black dotted squares): Class 3A (DRE and Ohler7) and Class 3B (INR2 and Ohler6). The two groups are correlated internally and anticorrelated with each other, indicating that the elements of each group bind a complex together. The remaining elements of Class 3, TTGTT, revTTGTT, revINR2, and E‐box1 show weak positive correlations to all other elements of their class suggesting that both transcription initiating complexes acting in this class have overlapping subunits.The four core promoter architectures identified. Class 1 motifs (INR, MTEDPE, CGpal, GAGA, GAGArev) occur in genes with NP core promoters (Architecture 1, 3,976 genes). The enriched genes are intermediately regulated and show strong correlations to stalled Pol II. Class 2 motifs including TATA‐Box and ATGAA also present in NP promoter genes; however, the enriched genes are strongly regulated ones that are either not expressed or most highly expressed in at least one developmental stage (Ar.2, 815 genes). Class 3 motifs (INR2, Ohler6, DRE, Ohler7, E‐Box1, TTGTT, TTGTTrev, INR2rev, AAG3) are the ones only found in genes with BP core promoters (Ar.3, 5,170 genes). The enriched genes are not regulated and similarly expressed in all developmental stages (housekeeping function). Ar.3 can be further subdivided in two additional sub‐architectures Ar.3.1 and Ar.3.2, as discussed in B. Class 4 motifs (TCT, RDPE) correlate with strongly expressed genes which mainly encode the ribosomal proteins (Ar.4, 64 genes).

**Figure EV4:**
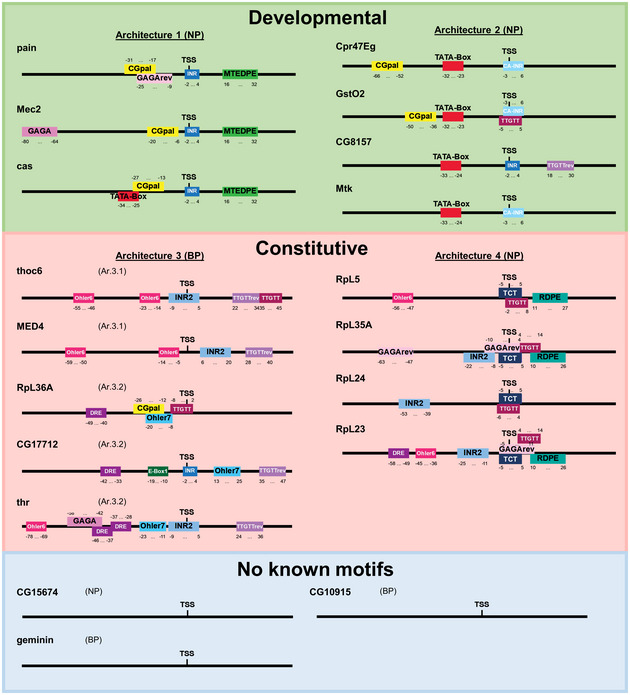
The wild‐type core promoters selected in this study and their motif composition Two‐to‐four native sequences were chosen (position −80 to +50 relative to TSS; TSS itself at position 0) from each of the four core promoter architectures Ar.1, Ar.2, Ar.3 (Ar.3.1, Ar.3.2), Ar.4 defined in Fig EV3, and one additional architecture with no known motif (termed motif‐less promoters). In total, 19 wild‐type core promoters with annotated motif positions are shown here. NP, narrow peak; BP, broad peak. Their sequences are listed in Appendix Table S2. Developmental and constitutive promoters are highlighted in green and red, respectively. Motif‐less promoters in blue.

After having identified CPEs, we then used available data on expression strength, regulation of expression, developmental stage of expression, polymerase stalling, and peakedness of the transcription initiation cluster to define gene sets that allow us to analyze correlations to specific sets of CPEs (Fig EV3 and Appendix Fig S2). To assure high‐quality sets, we derived an expression‐independent score for the peakedness of transcription initiation patterns (MAD score) and separated expression classes by analyzing their distribution. By correlating all identified motifs to the gene sets (Fig EV3A and B, Appendix Fig S2), four architectures of core promoter motifs could be defined (Ar.1 – Ar.4, Fig EV3C). Based on their association with gene functions (Zabidi *et al*, 2015), two architectures could be attributed to developmental promoters (Ar.1, Ar.2, 7 promoters selected in this study, details in Fig EV4), the two other architectures to constitutive ones (Ar.3, Ar.4, 9 promoters selected). We also found an additional class of promoters containing no known motifs (three promoters selected). Finally, to analyze the differences of the physical properties of the four core promoter classes, we computed the positioned dinucleotide frequencies within a large nucleotide window (500 bps) around the TSS (Appendix Fig S3 and Materials and Methods). All architectures show a strong composition bias for A and T containing dinucleotides, preferentially for “AA” and “TT”, adjacent to the core promoter region located between −100 and +50 bps with respect to the TSS. However, the classes vary strongly in the shape of A/T enrichment and the most frequently occurring dinucleotides (Appendix Fig S3). To conclude, the four core promoter architectures are distinctive in motif composition, gene features, and physical properties of the DNA.

### Design of synthetic promoter sequences

The synthetic promoter sequences were designed to test three different features separately: core promoter sequence features (especially motifs), influence of genomic ±1 nucleosomal flanking sequences, and transcriptional response to external stimulus. The synthetic promoter sequences were inserted into constructs made of combined building sequence blocks, which comprise different functional regions (Fig 1A and B): (1) a motif‐rich core promoter region of 130 bp around the TSS with native and perturbed sequences from different core promoter architectures (referred to as *block 3‐6*); (2) genomic −1 and +1 nucleosome positioning sequences to mimic the endogenous ±1 nucleosomal context (referred as *block 1* and *7*, respectively), and (3) a stimulus‐response element for binding of the ecdysone receptors to recruit the steroid hormone ecdysone for transcriptional activation (*block 2)*.

**Figure 3:**
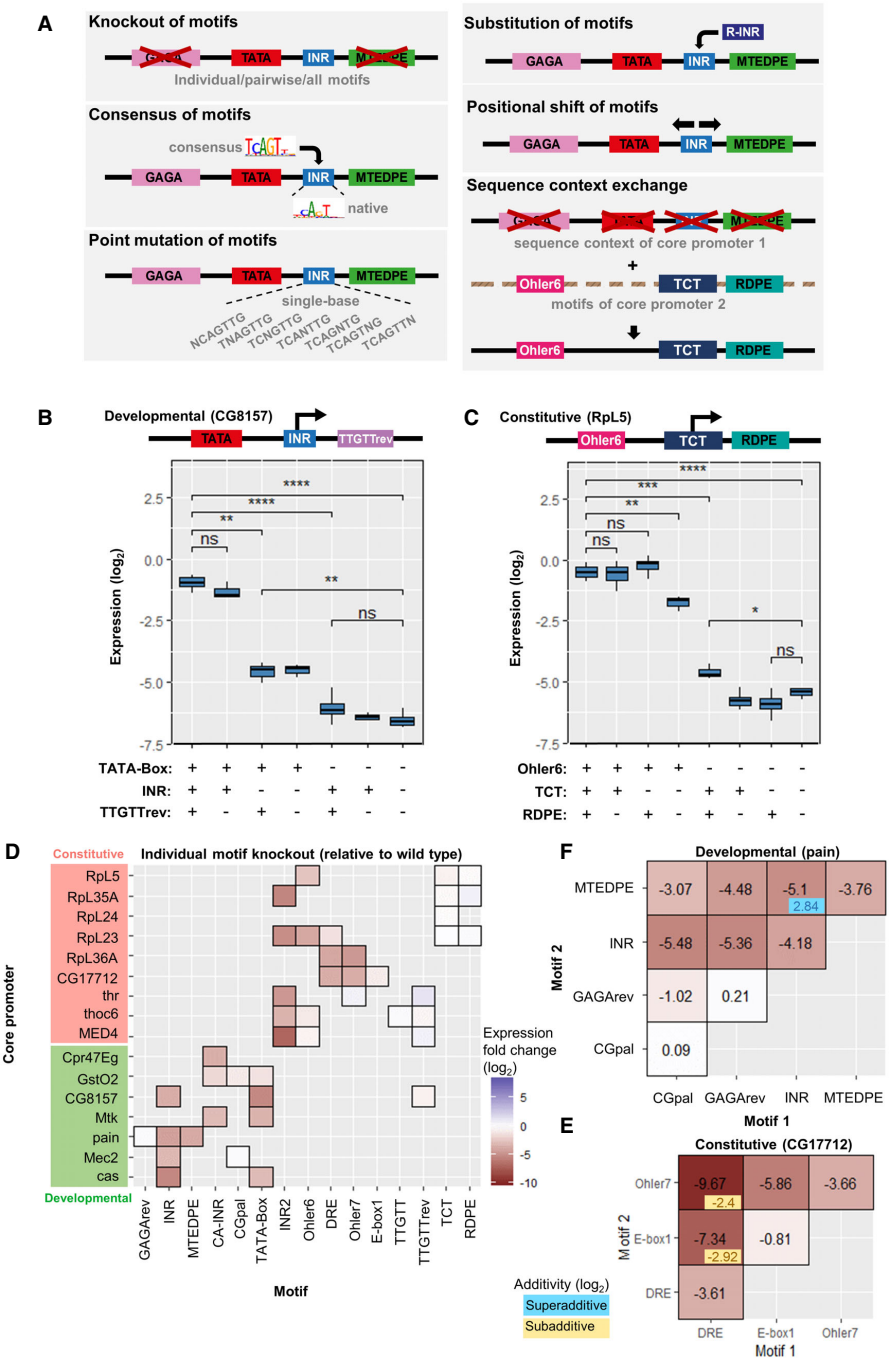
Combinatorial mutations designed for the motif‐rich core promoter region and results for motif knockout AMotif‐wise combinatorial mutations within the core promoter: motif strength and motif position are changed individually. From top to bottom: knockout of motifs (individual or pairwise knockout of motifs, and knockout of all motifs); replacing the original motif with its computationally (*XXmotif*) derived sequences with different PWM scores (consensus with the highest score), or insertion of the consensus into the motif‐less promoter sequences; point mutation of motifs; substitution with functionally or positionally equivalent motifs from other architectures; shift of motif positions; sequence context exchange between different core promoters. The *Mec2* motif composition is shown here as an example.B, CComparison of normalized expression levels between wild‐type configuration and motif knockouts for two types of core promoters (developmental: CG8157 (B); constitutive: RpL5 (C)). Upper panels: schematic depiction of the wild‐type motif compositions (TTGTT motif in RpL5 is ignored due to its strong overlap with R‐INR). Two‐sample t‐test: ns, not significant, *P ≤ 0.05; **P ≤ 0.01; ***P ≤ 0.001; ****P ≤ 0.0001. The middle hinge represents the median. The interquartile range the difference between the 75^th^ and 25^th^ percentiles. 3–4 biological replicate measurements.DMean expression fold changes compared to wild‐type expressions for individual knockout of motifs in different core promoters. Constitutive and developmental promoters are highlighted in red and green, respectively.E, FEffect of pairwise motif knockout (log_2_ scale) in core promoters CG7712 (E) and pain (F), respectively. The heatmaps display the mean expression fold changes compared to wild‐type expressions for pairwise knockout of motifs compared to individual knockouts (diagonals). Additivity was calculated as the difference between the pairwise effect and the sum of two individual effects, subadditive (in blue): > 0; superadditive (in yellow): < 0; Additivity values for effects > 3 × SD_noise_ shown in the right lower corner of each pairwise effect.

**Figure 6:**
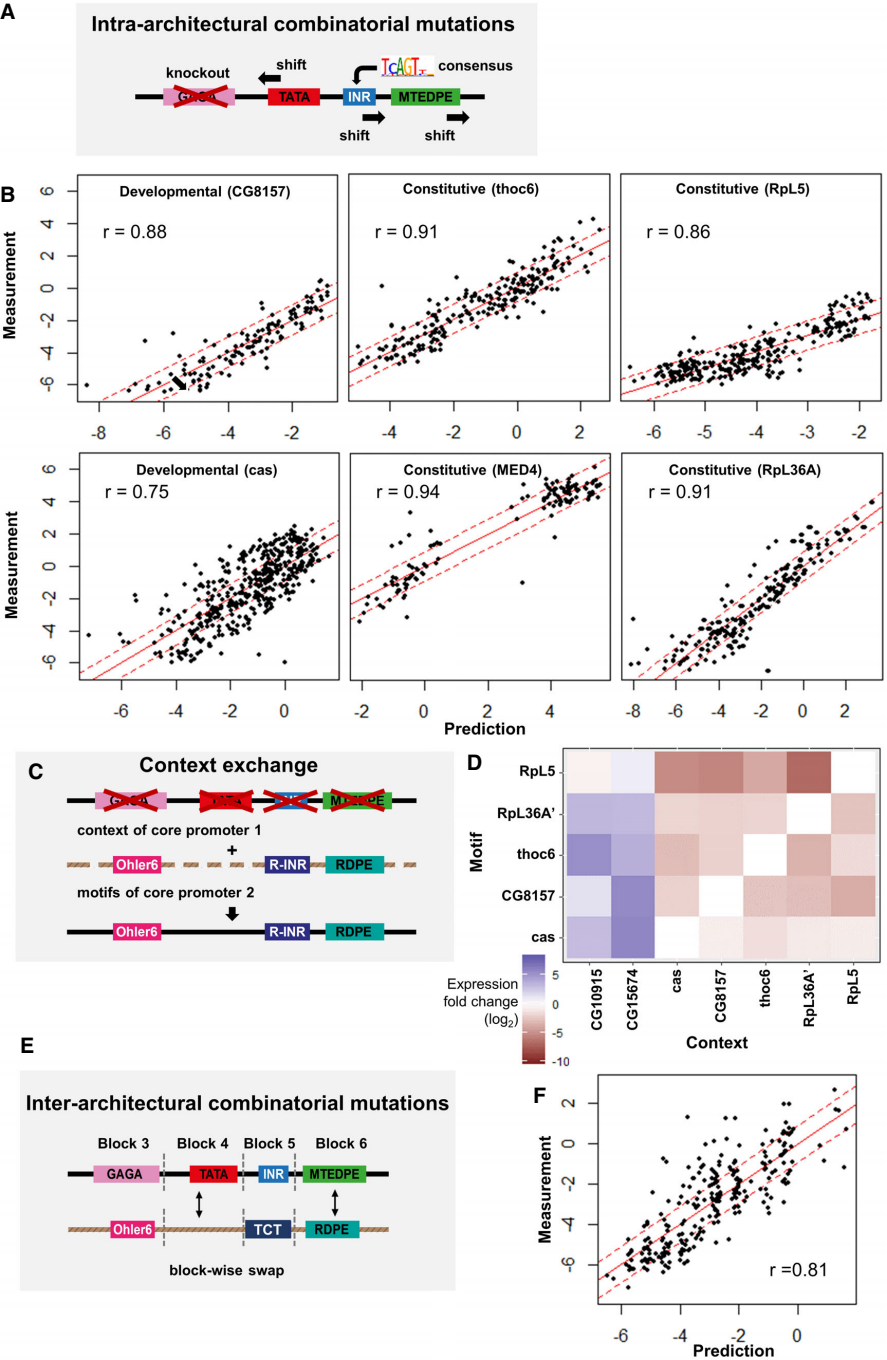
Linear regression modeling Intra‐architectural mutations: both change of motif strength and motif position within the same construct. The Mec2 motif composition is shown here as an example.Linear regression applied to predict the synthetic promoter activity based on individual motif features (intra‐architectural mutations). The measured expressions (on the y‐axis) for 6 tested core promoter sequences with combinatorial motif mutations compared to the predicted expressions (on the x‐axis) from the linear regression (log_2_ scale). Red solid line: y = x; red dashed lines: y = x ± 3 × SD, where SD denotes the median of all standard deviations over all measured synthetic promoter constructs. It is an estimate for the noise in the expression measurements. The linear regression model can explain on average 88% of the variance in expression (average *r* = 0.88).Context exchange between different core promoters: the motifs of promoter 2 with their respective relative distance are conserved and are incorporated in the sequence context of promoter 1.Effect of motif context sequence exchange. Heatmap depicting the mean expression fold changes caused by motifs (y‐axis) inserting of RpL5, RpL36A, thoc6, CG8157, and cas to different context sequences (x‐axis). The heatmap shows the expression changes relative to wild‐type expressions of the context‐origin promoters CG10915, CG15674, cas, CG8157, thoc6, RpL36A’, and RpL5, respectively.Inter‐architectural mutations: block‐wise combinatorial mutations between different core promoters. The motifs together with their sequence context within a block are swapped with others.Linear regression analysis for inter‐architectural block‐wise combinatorial mutations. The measured expressions (on the y‐axis) for inter‐architectural block‐wise combinatorial mutations compared to the predicted expressions (on the x‐axis) from the linear regression fit (log_2_ scale). Red solid line: y = x; red dashed lines: y = x ± 3 × SD, where SD denotes the median of all standard deviations over all measured synthetic promoter constructs. Pearson coefficient 0.81.

*Block 2* contains three EcR/USP heterodimer binding sites with 17 bp spacers in between (Materials and Methods). We found that this configuration responds the strongest to activation. All *block 1*s and *block 7*s are native *D. melanogaster* nucleosomal sequences selected to provide a variety of nucleosome occupancies (Heron, 2017). To systematically examine the sequence motifs of the motif‐rich core promoter (*block 3‐6*), we devised various mutations of wild‐type promoters (Figs 3A and 6A, C and E), including individual or pairwise knockout (complete replacement with non‐functional sequences) of motifs, knockout of all motifs, replacing the original motif with its *XXmotif*‐derived highest frequent genomic sequence (hereafter referred to as consensus), point mutations of motifs, shift of motif positions, and substitution with functionally or positionally equivalent motifs from other architectures (Fig 3A). In addition to widely known motifs like INR and TATA‐Box, we also tested four of the new motif candidates discovered by *XXmotif* (CGpal, TTGTT, TTGTTrev, and RDPE; Fig EV2A and Appendix Table S1). We compared the activities measured from synthetic promoters containing mutated motifs with the corresponding wild‐type strengths. The results obtained with the point mutations allowed an analysis of motif specificity. Recent studies on TF binding suggest that the sequence motifs alone cannot fully explain the activity variation (Schone *et al*, 2018; Yella *et al*, 2018). Therefore, we also tested in our experiments the context sequences surrounding the motifs (Fig 6C). Finally, combinatorial mutations altering both strength and positioning of all motifs within core promoter architectures (Fig 6A) as well as block‐wise swaps between architectures (Fig 6E) were performed for more in‐depth analysis which enabled quantitative modeling of promoter activity based on individual sequence features. The *block 3‐6* sequences were assembled with one inducible *block 2* and different combinations of *block 1* and *7* nucleosomal sequences, constructing the entire library of synthetic promoters to be tested in our experiments (Fig 1 and Dataset EV1).

### Core promoter activity measurements for thousands of designed sequences

We applied our method to produce and measure both basal and induced expressions from synthesized oligonucleotides representing wild‐type (Appendix Table S2) and mutated core promoters. We designed in total 3826 synthetic promoter sequences (Appendix Table S3 for an overview) and were able to recover and test experimentally ~3,000 of these sequences (the core promoter sequences were available multiplexed and the recovery of all sequences is experimentally not possible; see Materials and Methods). For most of the constructs (> 88%), we measured with and without ecdysone stimulation at least three replicates each. The expression levels range over more than four orders of magnitude and have a very high reproducibility among replicates, with a mean coefficient of variance (CV) of 21% for all the measurements (Fig EV1D for an example). To determine the activity level range of the native core promoters (Appendix Fig S1D), we measured the constructs containing all wild‐type (i.e., native) promoter sequences (given a fixed combination of *blocks* 1 and 7 defined below) (Fig 1E). The expression levels showed a broad range that spanned over three orders of magnitude. Two housekeeping core promoters MED4 and CG17712 drove the highest expressions, while the ribosomal class generally showed an intermediate activity. Strikingly, the core promoters with no known motif showed the lowest activity (in blue in Fig 1E). The low number of selected promoters does not however allow for a relevance analysis.

### Activity of our synthetic promoters in developing *Drosophila melanogaster* embryos

To check whether our synthetic promoters exhibit similar activity in an *in vivo* context, we measured their activity in developing *D. melanogaster* embryos using a fluorescent protein reporter for gene expression we recently developed (Ceolin *et al*, 2020) (Fig 1F–I). To this aim, we created in total 9 fly lines carrying different core promoters. The promoter sequences were cloned in the reporter vector downstream of an *hb_ant* enhancer, which is active in the anterior half of the embryo (Segal *et al*, 2008). Each construct was integrated at the same site in the fly genome. The tested promoters included different mutations (and also replacement with motif consensus sequences) for one constitutive (MED4) and one developmental gene promoter (pain). We imaged embryos in triplicates using a confocal microscope and performed data analysis, as previously described to quantify the protein fluorescence (Ceolin *et al*, 2020) (Materials and Methods). Representative confocal sections for each fly line are presented in Fig 1F; a signal mostly localized in the nuclei (white arrows) is observed for both promoter groups. No signal can be detected in a wild‐type embryo imaged as negative control (lower left corner of Fig 1F). The intensity of the fluorescence increases with the expression levels of the promoters, as measured by our luciferase assay pipeline (indicated in brackets in log_2_ scale in Fig 1F). To quantify the signal, we defined bins corresponding to 2% of egg length along a line connecting the anterior and posterior tips of the embryo and plotted the fluorescence intensity profile along the AP axis; for simplicity, only data from the dorsal side of the embryo were used (Fig 1G and H). The MED4 mutants (Fig 1G) exhibit a relatively homogeneous fluorescence intensity with a slight enhanced signal at the anterior tip (black arrow). By contrast, the pain mutants (Fig 1H) show a decreasing fluorescence signal going from anterior to posterior (black arrow), which recapitulates the known spatiotemporal activity of the *hb_ant* enhancer (Lucas *et al*, 2013) (indicated in black in Fig 1H and measured with a reporter construct containing the *hb_ant* enhancer and the DSCP synthetic promoter (Pfeiffer *et al*, 2010)). Interestingly, INR and an MEDPE are two common motifs in the pain (Fig EV4) and DSCP core promoters, whereas MED4 has a completely different motif composition including two Ohlers motifs (INR2 and TTGTrev) (Fig EV4). This could explain the different behaviors of pain and MED4 in these *in vivo* experiments as different players could activate the two core promoters at different time points and strength during development. Finally, we plotted the expression levels obtained in S2 cells with our luciferase assay as a function of the average fluorescence intensities along the AP axis for all the constructs (Fig 1I) and obtained a fair correlation (Pearson coefficient 0.91). Hence, our *in vivo* experiments confirmed the *in cellulo* results, and one observes in embryos strong difference in behaviors for the constitutive and the developmental genes selected.

### Influence of nucleosomal sequences on promoter activity

**Figure 2:**
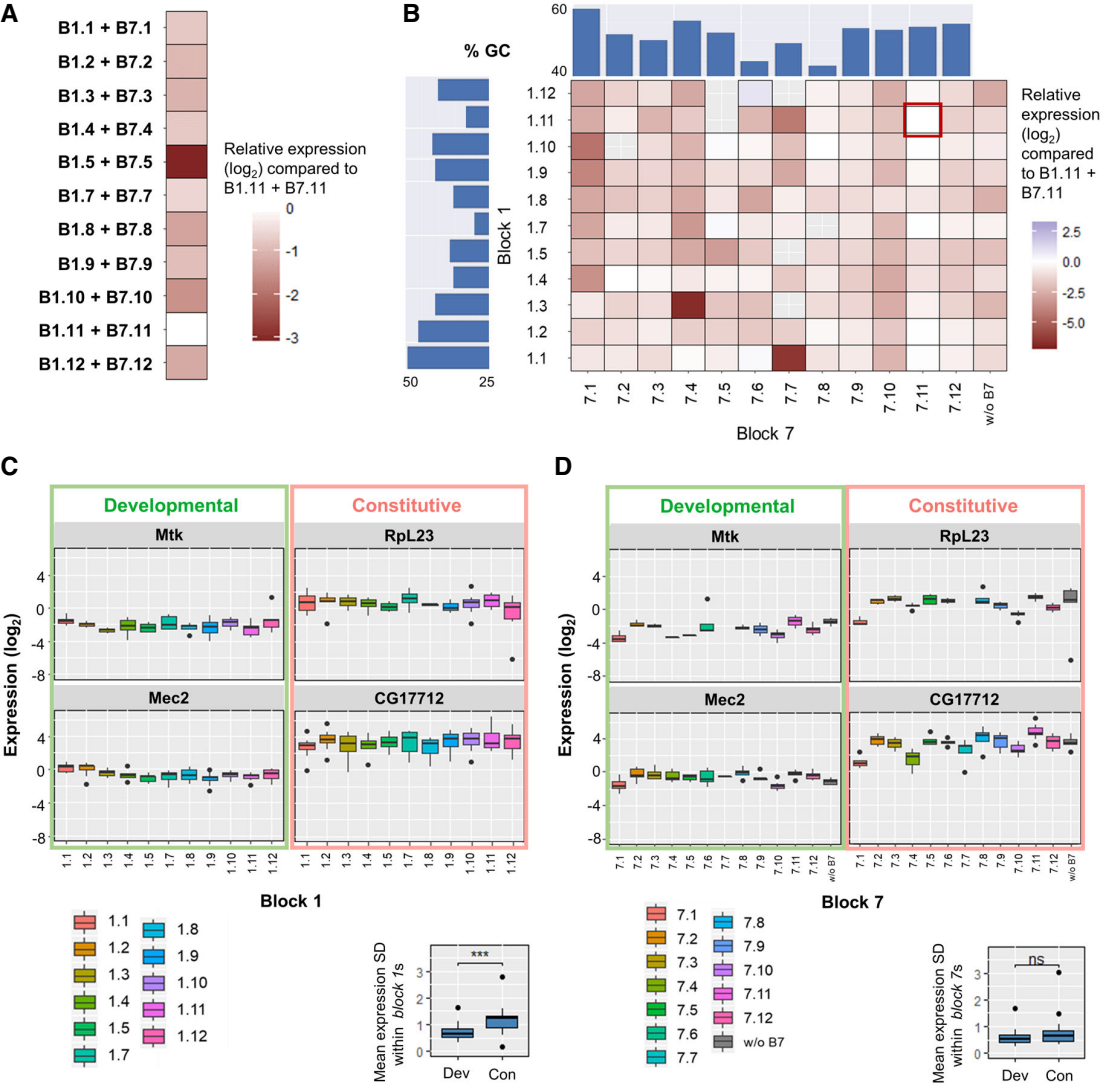
Expression levels of the native core promoters and the effect of nucleosomal sequence context on expression Heatmap depicting the relative expression level measurements of promoter constructs with different pairs of the nucleosomal sequences block 1 and block 7 compared to *B1.11* + *B7.11* expressions (log_2_ scale). Results were pooled for all tested native core promoters to calculate the average deviation to *B1.11* + *B7.11* expressions.Heatmap depicting the relative expression level measurements of promoter constructs with different free combinations of *block 1* and *block 7* compared to *B1.11* + *B7.11* expressions (marked with a red rectangle). Results were pooled for all tested native core promoters to calculate the average deviation to *B1.11* + *B7.11* expressions. Bar plots on the top and the left represent the GC content of each *block 1* and *block 7* sequence. *Block 7* with column “w/o B7” represents the results obtained from promoters without block 7 sequence.Boxplots depicting *block 1* effects for tested core promoters. Effects of different *block 7*s were merged in each column (within the same *block 1*): the median SD is 0.66 for developmental promoters compared to 1.23 for constitutive promoters (lower right corner); Wilcoxon rank‐sum test ***P = 3.1 × 10^−4^, significant. The middle hinge represents the median. The interquartile range the difference between the 75^th^ and 25^th^ percentiles. Individual points represent values over 1.5 times the interquartile range. 3–4 biological replicate measurements.Boxplots depicting *block 7* effects for tested core promoters. Effects of different *block 1*s were merged in each column (within the same *block 7*): the median SD is 0.54 for developmental promoters compared to 0.64 for constitutive promoters (lower right corner); Wilcoxon rank‐sum test *P* = 0.3, not significant. Block 7 with column “w/o B7” represents the results obtained from promoters without block 7 sequence. Developmental and constitutive promoters are highlighted in green and red, respectively. The middle hinge represents the median. The interquartile range the difference between the 75^th^ and 25^th^ percentiles. Individual points represent values over 1.5 times the interquartile range. 3–4 biological replicate measurements.

We then checked the influence of different nucleosomal contexts on the expression level of five native core promoters (Mtk, RpL23, Mec2, CG17712, and geminin) selected from the different architectures such that their activities covered the entire dynamic range of our measurements. Probing with our assay the pairwise *block 1.X* and *7.X* (with *X* an arbitrary index corresponding to the gene selected for their nucleosomal sequences; Appendix Table S4 and S5) showed that the paired *block 1.11* and *block 7.11* (hereafter termed as *B1.11* + *B7.11*) gave the highest average expression for the five genes (Fig 2A). Using *MNase*‐Seq, we measured the nucleosome occupancy on the plasmid of the synthetic promoter construct containing this pair (Appendix Fig S4A, higher panel): Nucleosome patterns were visible on the *B1.11* and *B7.11* sequences and were similar to what was observed at the genomic locus (Appendix Fig S4A, lower panel). Therefore, this *B1.11* + *B7.11* combination was selected as the fixed nucleosomal context sequence for highly mutated *block 3‐6*s in the subsequent experiments described below.

We however first explored the effect on expression of the free combination of the different *blocks 1.X* and *7*.*Y* (Fig 2B; Appendix Tables S4 and S5 for the sequences). As expected, we observed lower average activities compared to the constructs containing combinations *B1.11* + *B7.11* (an average signal reduction > 2.5‐fold). We computed the GC content of each *block 1*s and *7*s (in blue in Fig 2B), speculating that as the GC content usually correlates well with nucleosome occupancy it might correlate with our expression data. Nevertherless, we could not find any clear relationship between GC content of the different *block 1*s and *7*s and the expression levels. To test whether or not the presence or absence of *block 7*s has a strong influence on the expression levels, we also checked core promoter sequences with *block 1*s variants only and no *block 7* (Appendix Fig S4B). In these constructs, the length of the 5′ UTR was thus reduced from 333 nt to 89 nt. We observed that the expression levels of different mutated core promoters (randomly chosen) with or without *block 7.11* sequence were in the same order of magnitude, whether induced by ecdysone or not (Appendix Fig S4B; all constructs contained *block 1*.11 kept constant, *PCC r = *0.96, *P = *1.2 × 10^−5^). Hence, the presence of *block 7* has a relatively limited influence on expression level.

After having evaluated the overall effect of different nucleosomal sequences, we next explored potential promoter specificity. Indeed, the two tested constitutive promoters RpL23 and CG17712 exhibited stronger expression variations when altering *block 7s* (Fig 2C; the median SD within the same *block 1* is 1.23 compared to 0.66 for developmental promoters; Wilcoxon rank‐sum test *P = *3.1 × 10^−4^). By contrast, both the constitutive and developmental promoters showed similar and milder expression fluctuations upon *block 1* variation (Fig 2D; the median SDs within the same *block 7* for constitutive and developmental promoters are 0.64 and 0.54, respectively; Wilcoxon rank‐sum test *P = *0.3, not significant). The sequence downstream the TSS (*B7.X*) forming a prominent +1 nucleosome may set a transcriptional obstacle. It may also influence post‐transcriptional events as it constitutes the main component of the 5′ UTR region. Previous genome‐wide studies showed constitutive promoters tend to have a preferred canonical nucleosome pattern with a strongly positioned +1 nucleosome (Mavrich *et al*, 2008; Rach *et al*, 2011). This would explain why *block 7s*, which were designed in our experiments to act as different potential +1 nucleosomes, have a more prominent influence on constitutive promoter activities.

In overall, different potential nucleosomal contexts showed moderate effects on expression levels with a stronger effect for sequences potentially forming +1 nucleosomes. Constitutive core promotes were more sensitive to the influence of nucleosomal sequences downstream of the TSS.

### Knockout of motifs generally decreases expression consistently between core promoters

To find out whether motif knockouts significantly affect expression, we compared the expression levels of the wild‐type configuration with individual, pairwise, and all‐motif knockouts (Fig 3A). The disruption of well‐known motifs such as INR and TATA Box in CG8157 (Fig 3B) or Ohler6 in RpL5 (Fig 3C) reduces activity substantially. The only exception is the initiator for the ribosomal protein genes (TCT) that showed no significant effect when mutated in RpL5 (Fig 3C) or in any other tested ribosomal core promoter (Fig 3D). A similar absence of effect was observed for the RDPE motif, while a knockout of both motifs did cause a decrease in expression in RpL5 (~2.4‐fold reduction; Fig 3C). Disrupting all motifs in both promoters led to much weaker expressions (> 30‐fold decrease) (Fig 3B and C).

More generally, the knockout of all motifs resulted in a near complete loss of function for each tested core promoter sequences, regardless of its wild‐type strength (Appendix Fig S5A). Most of these all‐motif knockout configurations exhibited lower activity than wild‐type promoters containing no known motif. Compared to wild‐type expression, knocking out individual motifs typically resulted in a reduction (Fig 3D). These effects were consistent across the different promoters. An exception is the knockout of TTGTTrev that slightly increased expression in Thr and MED4 (the blue in the middle right of Fig 3D; > 2‐fold increase after disruption of the motif). Hence, this motif functioned as a weak repressor in these promoters. The core of TTGTTrev (AACAA) matches the central part of the binding site of an adult enhancer factor (AEF‐1) in *D. melanogaster* which is known to be a short‐range transcriptional repressor (Falb & Maniatis, 1992a, 1992b; Brodu *et al*, 2001). Finally, the ribosomal promoter motifs TCT and RDPE did not lead to a reduction of activity after their disruption in all the four investigated constitutive promoters (top right corner in Fig 3D).

### Pairwise knockouts of some motifs show synergistic (superadditive) effects

To investigate the role of motif interplay on regulating the expression, we compared the results obtained from pairwise knockouts with their individual knockout measurements in different core promoter configurations. Overall, the effect of most pairwise knockouts was additive (in log scale; Fig 3E and F and Appendix Fig S5B–G). However, in some cases, the expression levels were greater or less than the sum of the individual effects (super‐ and sub‐additive effects, respectively). For instance, the motif pairs DRE + Ohler7 and DRE + E‐Box1 in promoter CG17712 showed strong synergistic interactions (Fig 3E): the double knockouts yielded respectively a 2^2.4^‐fold and 2^2.9^‐fold lower expression than the repression expected from their independent, added effects on log2 expression. DRE is considered the most crucial motif in this housekeeping core promoter architecture as it directs a specific DREF binding TF (Hirose *et al*, 1993). The strong superadditivity we observed suggests the existence of a compensatory phenomenon for DREF binding involving Ohler7 and/or E‐Box1 against potential mutations of the DRE motif. Ohler7 could fully rescue the activity when E‐Box1 was disrupted, but not *vice versa* (CG17712 in Fig 3E). Nevertheless, core motifs in developmental promoters such as INR and MTEDPE in the pain promoter (Fig 3F), or INR2 and Ohler6 in the RpL23 promoter (Appendix Fig S5F) are so crucial for expression activity that a knockout of either resulted in almost the same effect as disrupting them both (subadditivity). For promoters GstO2, thoc6, and MED4, the pairwise effects showed exclusively linear additivity (Appendix Fig S5B–D).

Taken together, these results demonstrate that the disruption of some motif pairs in a given core promoter leads to synergistic effects. DRE is crucial for housekeeping promoter function, and the other three housekeeping motifs Ohler6, Ohler7, and INR2 also play essential roles in regulating ribosomal gene transcription.

### Most motif consensus sequences drive higher expression

**Figure 4:**
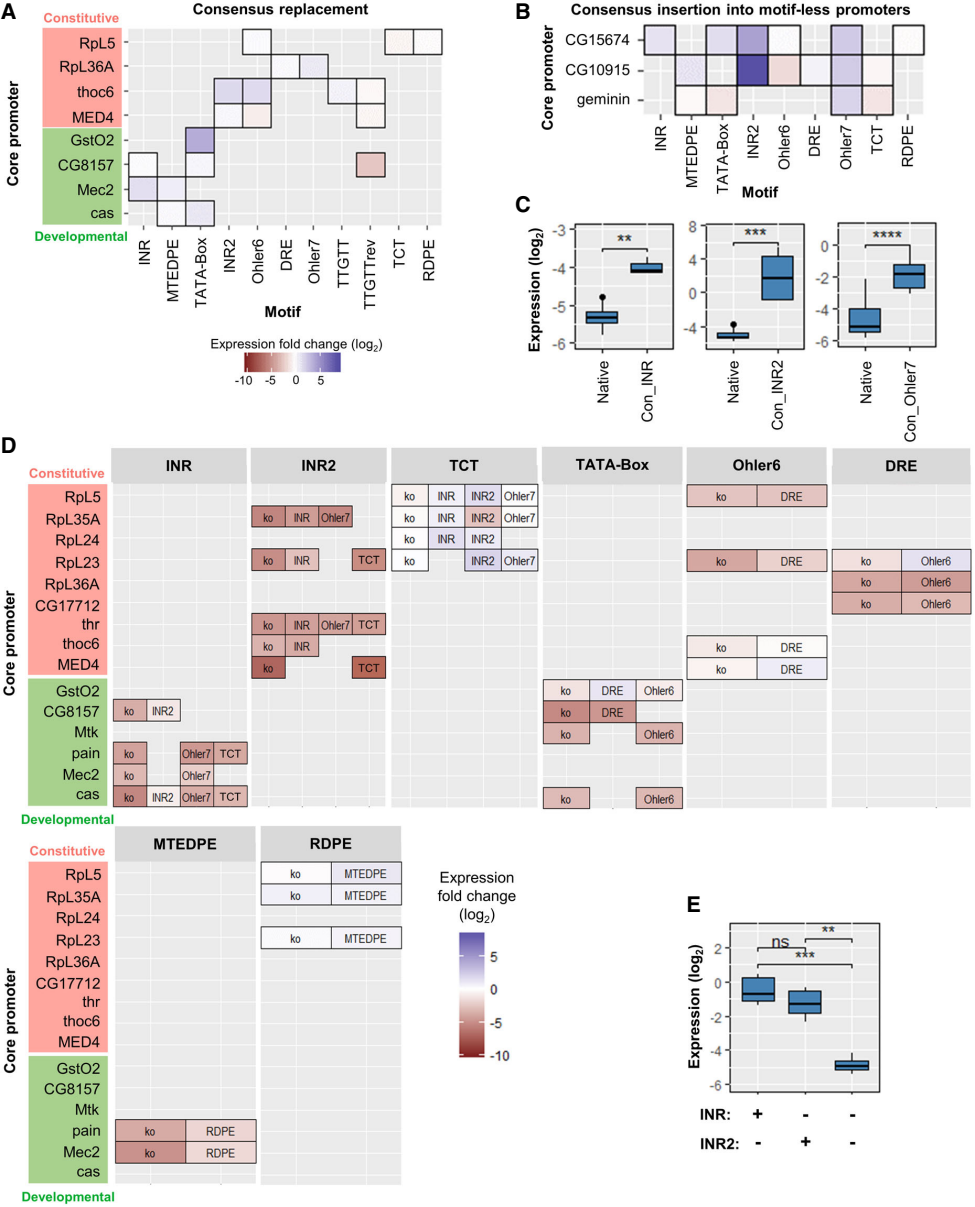
Consensus replacement and insertion into motif‐less promoters. Effect of motif substitutions Consensus replacement. Heatmap depicting the mean expression fold changes compared to wild‐type expressions after replacing with motif consensus sequences derived by *XXmotif*. Constitutive and developmental promoters are highlighted in red and green, respectively.Heatmap depicting the mean expression fold changes compared to wild‐type expressions after replacing consensus insertion into motif‐less core promoters.Boxplots depicting log expression change and significance level upon inserting consensus motifs of INR, INR2, and Ohler7 motifs (columns in A) into the core promoters (rows in A). Left panel: INR into CG15674 (two‐sample t‐test **P = 0.0033); middle panel: INR2 into CG10915 and CG15674 (Wilcoxon rank‐sum test ***P = 0.00018); right panel: Ohler7 into Geminin, CG10915, and CG15674 (Wilcoxon rank‐sum test ****P = 3.4 × 10^−5^). The middle hinge represents the median. The interquartile range the difference between the 75^th^ and 25^th^ percentiles. Individual points represent values over 1.5 times the interquartile range. 3–4 biological replicate measurements.Heatmap depicting the mean expression fold changes compared to wild‐type expressions for motif knockout and substitution with positionally or functionally equivalent motifs from other architectures. Constitutive and developmental promoters are highlighted in red and green, respectively.Boxplot depicting the effects of INR being substituted by INR2 in cas and CG8157 (all measurements in these two core promoter constructs were pooled together; Wilcoxon rank‐sum test **P = 0.0051 for comparing substitution with knockout (significant) and *P* = 0.17 for comparing substitution with wild‐type (not significant). The middle hinge represents the median. The interquartile range the difference between the 75^th^ and 25^th^ percentiles. 3–4 biological replicate measurements.

In addition to motif knockout, we tested if computationally derived consensus sequences that are preferred in the genome could increase expression (Fig 4A). Most consensus sequences drove higher promoter activity, especially the consensus of TATA‐Box in GstO2 (more than 15‐fold stronger expression; seen as dark blue square in Fig 4A). As an exception, replacing the TTGTTrev motifs with their consensus sequence in three promoters led to a signal reduction, again supporting its role as repressor (brown square in Fig 4A).

Because replacing most motifs with their consensus sequence increased expression levels, we asked whether these sequences could boost the activity of the motif‐less promoters (CG15674, CG10915, and geminin) (Fig 4B). Indeed, some motifs, particularly those containing a CA TSS site like INR, INR2, and Ohler7, were sufficient to significantly increase expression when inserted into these motif‐less promoters (Fig 4B and C; > 2‐fold increase for INR replacement, ~100‐fold increase for INR2 and ~5‐fold increase for Ohler7 on average). The other motifs did not affect or decreased the expression, maybe due to the disruption of sequences bound by unknown proteins. Overall, these results demonstrate positive effects on expression of most computationally derived motif consensus sequences (except the repressive TTGTTrev).

### The positionally or functionally equivalent core promoter motifs from other architectures can hardly function as endogenous sequences

While checking the features of core promoter motifs discovered by *XXmotif* (Appendix Table S1), we confirmed that certain motifs tend to locate in different core promoters within a similar region relative to TSS (like DRE and Ohler6 at around −100 to −7), or they share similar sequence features such as the “CA”s in INR, INR2 and Ohler7. We investigated whether positionally or functionally equivalent motifs (i.e., leading to similar decrease of expression after knockout) from other architectures could rescue the expression from knockouts. Three motif groups were tested: INR‐INR2‐Ohler7‐TCT; TATA‐Box‐Ohler6‐DRE; MTEDPE‐RDPE.

For most of the motifs, we found that substitution could not rescue the promoter activity, that is, substitution would yield the same or an only slightly higher expression than if the motif was knocked out (Fig 4D). An exception was the INR2, which could almost compensate for a INR knockout—showing a rescue effect (Fig 4D and E; Wilcoxon rank‐sum test *P = *0.17 between the native expression and the INR2‐substituted expression). Conversely, INR was not able to compensate for the loss of INR2 (Fig 4D). They both generally increased expression level compared to the native arrangement when substituting TCT. This is likely due to the low intrinsic expression levels of TCT‐containing promoters and matches the lack of TCT knockout effect.

### Systematic point mutations enable the generation of expression‐based PPMs and activity logos for core promoter motifs

**Figure 5:**
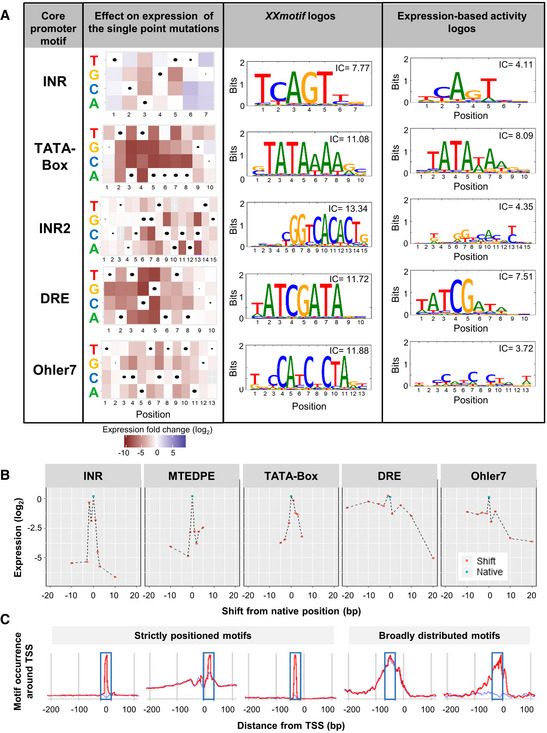
Point mutations and positional shift left panel: effect on expression of the single point mutation compared to the consensus sequence (indicated as dots whose size scales with the loss of expression after mutation). Middle and right panels: comparison of the *XXmotif* logos with the expression‐based activity logos for INR, TATA‐Box, INR2, DRE, and Ohler7. Expression‐based activity logos show an overall lower specificity. IC, information content.Effect of motif positional shifts. log_2_ expression of native promoters (cyan dots) and promoters with motifs shifted relative to their original locations (red dots), for INR, MTEDPE, TATA‐Box in cas, and DRE, Ohler7 in RpL36A.Motif occurrence around TSS (at position 0) discovered in the genome‐wide analysis by *XXmotif*. The blue rectangular boxes indicate the −20 to 20 bp region surrounding the original positions of the motifs in the tested core promoters (strictly positioned INR, MTEDPE, TATA‐Box in cas; broadly distributed DRE, Ohler7 in RpL36A).

We then systematically measured the influence on expression of all possible single base pair mutations of the motif consensus for various native promoters (details in Materials and Methods). We recovered nearly all of the variants for the motifs INR, TATA‐Box, INR2, DRE, and Ohler7. In most cases, the consensus sequences gave the highest expression levels (Fig 5A left panel). Based on these expression measurements, we generated PPMs, and thereby activity logos for these motifs, which we compared with their *XXmotif* sequence‐based logos (Fig 5A middle and right panels). For all motifs, the expression‐based consensus is identical to the computational one. All the expression‐based activity logos are less specific, as indicated by their lower information content IC (Fig 5A, upper right corners of the logos) compared to those found in silico by *XXmotif*. An exception were the CG nucleotides in the DRE motif that have higher information content than the equivalent positions in the motif generated by *XXmotif*, suggesting their function as the primary recognition site for DREF binding.

In summary, although the sequence computationally identified as over‐represented generally represents the best motif, the specificity of the individual nucleotides in the sequence tends to be overestimated. This is not surprising since computational motifs are derived from over representation in promoter subgroups, which induces a bias toward higher specificity to distinguish them. In addition, their specificities are tuned by how strict the method is in accepting weak–strength motifs as true binding sites.

### Precise positioning of motifs is an essential feature of core promoter function

The *XXmotif* analysis showed the strong positional preferences of some motifs (Appendix Table S1), as already reported by Ohler *et al* (2002), Ohler (2006), Rach *et al* (2009). To test the functional relevance, we shifted the motifs around their native positions and checked the consequences on expressions.

Overall, varying motif positions from their position in the examined native promoters decreased the expression level, regardless of the shift direction (Fig 5B). Additionally, the decrease in expression level correlated with the shift size. In the case of strongly positioned motifs (INR, MTEDPE, and TATA‐box), even small shifts (< 5 bp) led to a severe loss of expression, while less well‐positioned motifs (DRE, Ohler7) showed milder effects when shifted (Fig 5B). These position‐dependent expression patterns showed similar shapes as the genomic motif distribution within ± 20 bp region of the most enriched motif locations (Fig 5C).

In conclusion, the motif position is essential for core promoter function, because shifting affects the expression. Even single bp shifts can have strong effects. The genomic distributions of a motif reflect its measured expression pattern.

### A linear combination of individual motif features can largely explain the core promoter activity

Our results obtained from the pairwise knockout of motifs revealed the existence of superadditive or subadditive effects of individual motif features (Fig 3E and F, Appendix Fig S5E‐G). This prompted us to investigate how much of the expression level can be explained by the pure additive contributions of each motif feature. Therefore, we tested promoters combining all types of mutations (varying motif strength, shift, and replacement) given a core promoter architecture (termed intra‐architecture mutations; Fig 6A and B, Appendix Fig S6). We applied a linear regression analysis to predict log2 expression, assigning the covariate variables in the model as the qualitative indicators (0/1) of the individual mutation existence (Materials and Methods). We obtained an average correlation of 88% (6 promoters tested) between predicted and experimentally measured log2 expression levels (Fig 6B). The coefficients learned by the models also correlate with expression levels of single mutation promoter (average correlation *PCC r = *0.93; Appendix Fig S6A).

As a more direct test without any fitting procedure, we also built an additive model to predict the activity of a given promoter with the intra‐architectural combinatorial mutations based directly on the measurements of individual motif mutations (Appendix Fig S6B). The contribution of each feature (both motif strength and position) was assumed to be additive and was derived from the deviation between the corresponding motif‐mutated sample compared to the native expression. Except for one promoter (cas, for which multiple single mutation constructs were not recovered during the cloning procedure; Materials and Methods), we obtained a comparable mean correlation of 84%.

To conclude, our results suggest that the activity of a given synthetic core promoter is largely predicted from the linear combination of individual motif features. Both a linear regression model and a parameter‐free additive model can explain most of the variance in expression. However, deviations are still observed, revealing the complex interplay between the factors involved.

### Motif context in core promoters influences expression

In addition to mutations applied to sequence motifs, we also tested the influence of the motif context on the expression level, that is, the sequence environment surrounding the motifs in the core promoter region.

We first created promoter variants where either all motifs or motif contexts were shifted together, thus, maintaining the relative spacing of motifs while altering the sequence background in which they were located. In general, both cases led to reduced expression; the effects were comparable to, or weaker than those obtained from individual motif shifts (Appendix Fig S6C).

Besides the mutations applied within each native core promoter architecture, we also exchanged context sequences surrounding the motifs of a given promoter with foreign context sequences originating from other promoter architectures (Fig 6C). The analysis revealed that overall the motifs preferred their native contexts (Fig 6D). For instance, adding the motifs from RpL5 into any other promoter contexts resulted in on average more than 10‐fold reduction of the expression levels. When inserting motifs from any of the tested promoter architectures into motif‐less core promoters (CG10915 and CG15674), they drastically improved the expression with a maximum increase of more than 55‐folds (Fig 6D, blue squares). When comparing the obtained results with the wild‐type expressions of the motif‐origin promoters, the context from CG15674 could rescue or even increase the expression of developmental promoters with their native motifs (~25% expression increase for cas and > 2‐fold increase for CG8157). Similarly, the context from the motif‐less core promoter CG10915 could enhance promoter activity compared to the native Thoc6 (a constitutive promoter; with a ~2.5‐fold increase). Note that, although we checked whether the various context effects may be explained by the classification as narrow peak (NP) or broad peak (BP) promoters, we did not see a clear relationship.

Given the effects observed for motif contexts and the strong predictability of core promoter activity based on individual motifs, we wondered which role the context sequences surrounding the motifs play in defining core promoter function (inter‐architectural mutations, Fig 6E). We built constructs with random selections of the individual *blocks 3*, *4*, *5*, or *6*, respectively, for 5 different promoter architectures (Materials and Methods). Similarly to the analysis of the intra‐mutations, a linear regression model was learned directly from the expression measurements obtained from these block‐wise combinatorial mutations (Fig 6F; detailed mutation design in Materials and Methods). The predicted values also showed a high correlation with the measured expressions (*PCC r = *0.81, *P* < 2.2 × 10^−16^), supporting the additivity for sequence features even among various promoter architectures. The learned coefficients revealed the significance of the block features (Appendix Table S6), although some coefficients were not significant, probably due to too sparse data (not all inter‐architectural mutated promoter constructs were recovered during the cloning procedure; Materials and Methods). Surprisingly, *Block 5 sequence*s generally had a weak impact on the predictions (average *P* > 0.6; Appendix Table S6). As the tested *block 5* sequences always contain functionally similar motifs essential for transcription initiation (such as INR, INR2, CA‐INR, Ohler7, and R INR), our results indicate that binding to these motifs is retained, even by exchanging *block 5*. Ignoring *block 5*, the block variants with the strongest contributions to expression correlated with the influence on expressions of specific motifs inside these blocks. For instance, *block 4* in CG8157, *block 4* in RpL36AN, and *block 6* in Cas were the most significant features (*P* < 3×10^−7^) found in the model, in which TATA‐Box, DRE and MTEDPE motifs locate, respectively. They all increased the expression levels when replacing other blocks (average coefficient > 1.85). *Block 3* in RpL36AN, which contains DRE, gave a negative contribution (coefficient = −1.57, *P = *0.011). This indicates a possible positional preference for DRE to be located in *block 4*. The background sequences of *block 3* in CG8157 and Cpr47Eg (with a non‐functional CGpal) provided significantly negative effects (average coefficient < −2.4). *Block 6* in CG8157 with a TTGTTrev played a slightly negative role as well, which is again consistent with the repressive function of this motif (coefficient = −0.7, *P = *0.008). To summarize, our results show that the motifs do not contain all the information. The context sequences surrounding the motifs in core promoters also play an important role in defining the activity. These effects are however generally less prominent. The block sections, which contain motifs together with their surrounding context sequences, largely function linearly for setting expression levels.

### Ecdysone responsiveness correlates with the core promoter architecture

**Figure 7:**
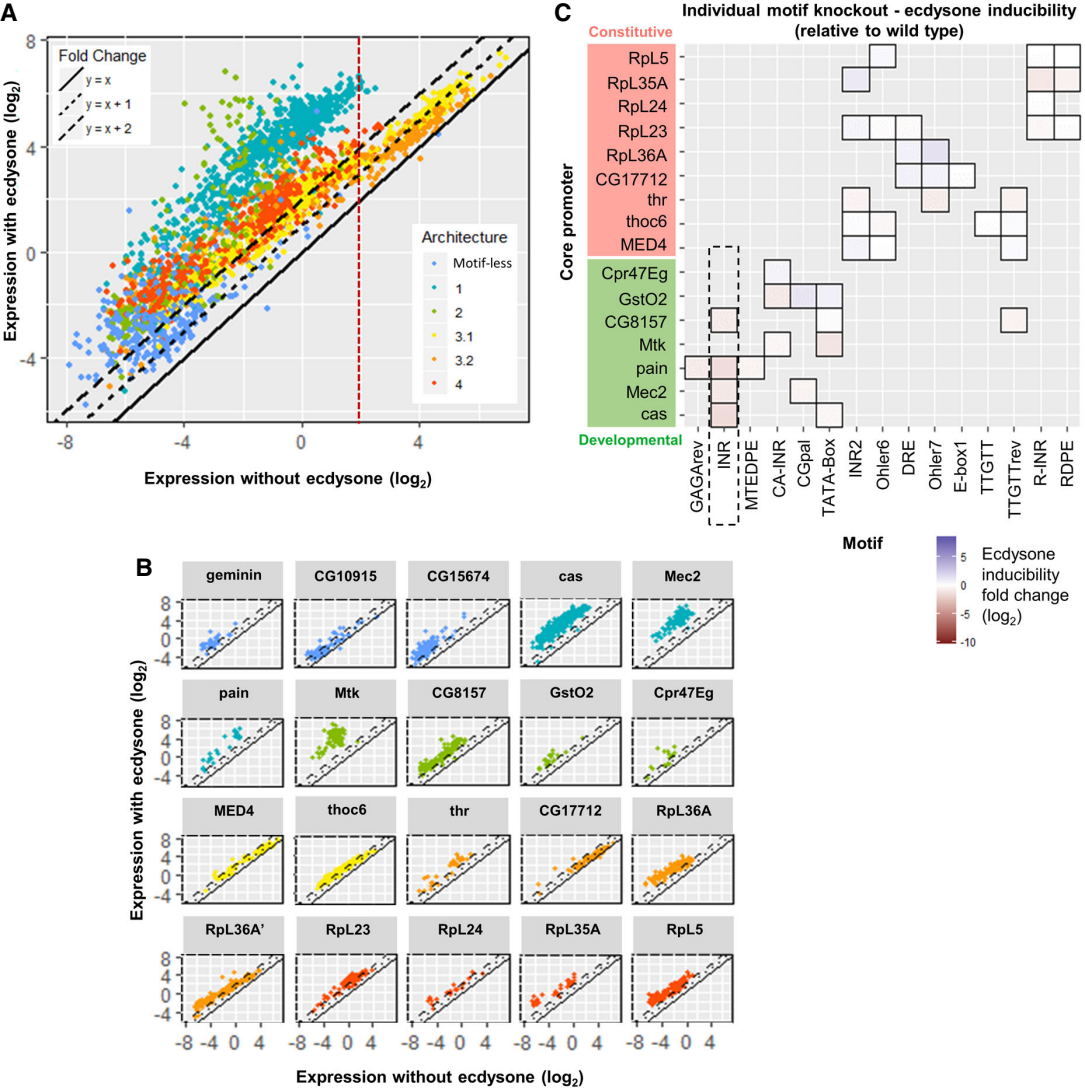
Ecdysone inducibility Scatterplot depicting the expression measurements with ecdysone induction versus measurements without ecdysone for all tested promoters separated by promoter architecture (Fig EV3c). Each color represents one architecture (color‐code indicated in the insert). Three types of line are used to indicate the expression fold change with no increase (y = x; solid line), 2‐fold increase (y = x + 1; dotted line), and 4‐fold increase (y = x + 2; dashed line). Red vertical dashed line: log_2_ basal expressions = 2. Log_2_ expressions > 2 on the right of the red dotted line.Expression fold changes (ecdysone inducibility) versus measurements without ecdysone and grouped by native core promoter sequences. The colors refer to different core promoter architectures. Three types of line are used to indicate the expression fold change with no increase (y = x; solid line), 2‐fold increase (y = x + 1; dotted line), and 4‐fold increase (y = x + 2; dashed line). Red vertical dashed line: log2 basal expressions = 2.Heatmap depicting the ecdysone inducibility fold changes caused by individual knockout of motifs in different core promoters. Disrupted INR (highlighted with the black dotted line rectangle) had a slightly negative effect on changing the core promoter responsiveness to ecdysone. (~2.3‐fold reduction on average, Wilcoxon rank‐sum test *P* = 2.1 × 10^−5^). Constitutive and developmental promoters highlighted in red and green, respectively.

**Figure EV5:**
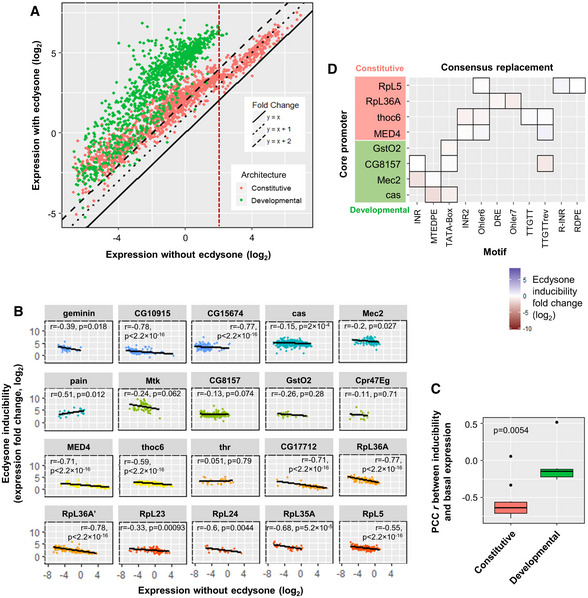
Ecdysone induction effect (log2 scale) grouped by promoter architectures Scatterplot depicting the expression measurements with ecdysone induction versus measurements without ecdysone for all tested promoters separated by core promoter architectures. Constitutive and developmental promoters are plotted in red and green, respectively. Three types of line are used to indicate the expression fold change with no increase (y = x; solid line), 2‐fold increase (y = x + 1; dotted line), and 4‐fold increase (y = x + 2; dashed line). Log_2_ expressions > 2 on the right of the red dotted line.Comparison of the expression fold changes versus measurement values without ecdysone for all native promoters and their mutated versions, grouped by native core promoter sequences. The colors refer to different core promoter architectures. Three types of line are used to indicate the expression fold change with no increase (y = x; solid line), 2‐fold increase (y = x + 1; dotted line), and 4‐fold increase (y = x + 2; dashed line).Comparison of the *PCC r*s obtained in A grouped by constitutive and developmental core promoters. Wilcoxon rank‐sum test *P* = 0.0054. The middle hinge represents the median. The interquartile range the difference between the 75^th^ and 25^th^ percentiles. Individual points represent values over 1.5 times the interquartile range. 3–4 biological replicate measurements.Heatmap depicting the ecdysone inducibility fold changes caused by consensus replacement of motifs in different core promoters. Constitutive and developmental promoters highlighted in red and green, respectively.

Finally, we checked the global ecdysone responsiveness for our entire synthetic promoter library. Ecdysone activation increased the expression level of almost all promoter candidates (both native and mutated) tested in our experiments (Fig 7A). The ecdysone responsiveness spanned a range of 1,000‐fold difference between the highest and lowest effect. We also found (Fig EV5A) that developmental core promoters (green dots in Fig EV5A) were highly induced with an average > 20‐fold activity increase, while constitutive core promoters (red dots in Fig EV5A) showed much weaker responses (around a 4‐fold increase on average). Given that ecdysone is a developmental stimulus, it should be expected to preferably activate developmental core promoters. Some housekeeping core promoters with already high basal expression levels without ecdysone stimulation (log_2_ expressions > 2; on the right of the red dotted line in Figs 7A and EV5A) exhibited much smaller activations, suggesting saturation of promoter expression level that cannot be further enhanced.

To gain deeper insight, we checked the ecdysone responsiveness of each promoter individually (Fig 7B). Here, the ecdysone responsiveness is defined as the ratio between the induced and uninduced expression level; also referred to as the ecdysone inducibility or the expression fold change caused by the ecdysone induction. We found a generally negative correlation between inducibility and expression level without ecdysone stimulation (Fig EV5B; the only exception was a group of sequences derived from pain core promoter, which had increased inducibility with higher expression; *r = *0.51; *P = *0.012). The higher the expression level, the lower the inducibility, which is consistent with the low activation measured for promoters with high basal expression level. The negative correlation was more significant for constitutive core promoters than developmental ones (Wilcoxon rank‐sum test *P = *0.0054; Fig EV5C).

The ecdysone inducibility was generally independent of nearly all single motif knockout mutations (Fig 7C) with the exception of INR (a slightly negative effect of ~2.3‐fold reduction on average, Wilcoxon rank‐sum test *P = *2.1 × 10^−5^). Similarly, the motif consensus sequences did not dramatically affect the ecdysone responsiveness (< 20% reduction on average; Fig EV5D).

Together, our results demonstrate a correlation between the ecdysone responsiveness and the core promoter architecture. Ecdysone can induce both developmental and constitutive core promoters but drives higher stimulations on developmental ones. The ecdysone inducibility generally decreases with the expression level for a given promoter: the higher the activity, the more difficult it seems to be to boost further expression level. Very strong promoters are barely inducible, probably due to promoter activity saturation. Finally, motif disruption has only minor influence on the ecdysone responsiveness of the core promoter.

## Discussion

Our results reinforce the conclusions drawn from other smaller scale studies for the roles of core promoter motifs in determining transcriptional output, also generalizing their effects to more promoter architectures. Nevertheless, the major contribution of this work is to bring new insights into *D. melanogaster* core promoter function.

First, based on the CPE classes identified by *XXmotif*, we define four core promoter architectures (Ar. 1–4), reflecting different modes of transcriptional regulation at the core promoter and different physical properties of the DNA. The co‐occurrence of CPEs within the classes indicates that each motif class recruits a specific transcription initiation complex utilizing several binding sites. One such example is the TFIID complex that assembles at the DNA due to interactions to the Class 1 elements INR bound by the subunits TAF1 and TAF2 (Ohler *et al*, 2002; FitzGerald *et al*, 2006) and the DPE element bound by the subunits TAF6 and TAF9 (Burke & Kadonaga, 1997). We propose that the remaining Class 1 elements also contribute to the binding of TFIID. Within Class 2, TATA‐boxes are known to be bound by TBP (Lifton *et al*, 1978), which is another part of the TFIID complex. Since TATA‐boxes are anti‐correlated to DPE, the novel ATGAA—positioned similarly to DPE—might replace it in Class 2 promoters. A similar hypothesis can be stated for Class 4 consisting of TCT and RDPE. As shown by Parry *et al* (2010), genes containing the TCT are not regulated by TFIID, but by a special RNA polymerase II system for ribosomal protein genes. The clustering also suggests two distinct preferred compositions in the 3rd class (INR2 + Ohler6 pair and DRE + Ohler7 pair).

We demonstrate that the well‐known functional motifs like INR, TATA‐Box, MTEDPE, INR2 (more widely known as Ohler1 or motif 1), DRE, and Ohler7 are necessary for gene expression. Their roles are unique and they cannot be replaced by positionally or functionally similar motifs from other architectures. Pairwise knockouts mostly elicit more significantly negative effects on transcription, and these effects show in some cases superadditivity. Conversely, most of the motif consensus sequences tend to increase core promoter activity. All these findings are consistent between different core promoters and emphasize again the importance of the sequence motifs for core promoter function.

However, not all well‐characterized motifs have a significant effect on expression in our measurements. This is especially the case with TCT, which stands in contrast with the strong loss of transcriptional activity observed by Parry *et al* (2010) in their mutational analysis. The differences may arise from transcription originating at another location on the reporter plasmid or differences in translation efficiency, as discussed above. However, TCT is the only CA less TSS‐motif and is part of a specialized TCT‐based Pol II transcription system, distinct from the INR‐based system (Parry *et al*, 2010). This might explain why this motif makes almost no contribution to promoter activity in our measurements, although it exists in nearly all ribosomal protein gene promoters in *D. melanogaster*. By contrast, housekeeping core promoter motifs like INR2 and Ohler6 that co‐occur in multiple promoters show stronger influence in our data. It is known that more than half of the ribosomal core promoters contain this INR2 motif (Ma *et al*, 2009). A recent study proposed that the INR2 binding protein M1BP can act as an intermediary factor to recruit TRF2 for proper transcription of ribosomal protein genes (Baumann & Gilmour, 2017). Our perturbation analysis of INR2 in various ribosomal promoter backgrounds supports their finding. The results we obtained with Ohler6 also suggest that the unknown TF(s) that bind to it may function similarly as M1BP.

Among the four tested novel motif candidates discovered by *XXmotif*, we identified TTGTTrev and RDPE as having measurable effects on expression after mutation, hereby confirming their biological relevance. TTGTTrev shares a similar function with a negative regulatory element for binding of a transcriptional repressor AEF‐1. The occurrence of RDPE is highly correlated with TCT and can partially replace the function of MTEDPE in developmental architectures. However, we note that the mutations in the two newly discovered motifs like TTGTT and CGpal show little effect on expression, suggesting that these two computationally derived over‐represented sequences lack functional importance as core promoter elements. They are therefore likely to represent binding sites of transcription factors that are not expressed in our experiments. Due to the similarity of TTGTT with TCT, this motif may act as a redundant version of the TCT motif.

Our highly sensitive assay can also accurately capture the partially subtle expression changes caused by single base‐pair variations of motifs. We confirm that the most over‐represented sequence of a given motif in the genome mainly stands for its best functional form, but we also saw differences with the computationally derived matrices: Our expression‐based activity logos are generally less specific. The two kinds of motifs are complementary since they reflect different phenomena: *In silico* discovered motifs are expected to reflect binding affinities, whereas the expression measurements capture the effect on transcription initiation, which could be buffered, for example, by alternative pathways/coactivator complexes.

Altering motif positions overall decreases expression. This phenomenon has been observed before by Schor *et al* (2017), who showed using CAGE measurements that changing the distance between motifs could have a major impact on transcriptional initiation and overall transcripts levels. More generally, Arnold *et al* (2017) demonstrated that the positional occurrence of specific 5‐mers relative to the TSS is predictive of the enhancer sequences’ responsiveness by a linear model, which is difficult to validate with our measurements due to too few data points for positioning compared to a deep sequencing method. Several studies have suggested that the exact spacing is essential for synergism between the core promoter motifs to function as active pairs to recruit GTFs along with Pol II for accurate transcription initiation (O'Shea‐Greenfield & Smale, 1992; Burke & Kadonaga, 1997; Emami *et al*, 1997; Gershenzon & Ioshikhes, 2005; Gershenzon *et al*, 2006). Our results are in line with these previous findings for strictly positioned motifs such as INR, MTEDPE, and TATA‐Box. Their locations and spacings are highly restricted for the effective binding of the TFIID to nucleate the PIC. Other motifs that can function over wide ranges and are not necessary for constituting the major machinery, for example, DRE, Ohler6, and Ohler7, show less stringent location requirement and smaller effects on expression, as long as they do not disrupt other sequence features.

Importantly, we also demonstrate that not only the core promoter motifs but also their context sequences determine expression output, giving insights into the debated role of motif flankings and context sequences of core promoters. Our results uncover that sequence motifs mostly prefer their native context. Remarkably, although only INR and INR‐like motifs including INR2 and Ohler7 can drive higher expression when their consensus sequences are inserted into motif‐less core promoters, the motif combinations from almost all the other defined architectures can result in a substantial increase of expression level, revealing the importance of motif synergism. We did not test motif activity in random sequence context. We nevertheless see an influence of the sequence context independent of the motifs, which may obey complicated rules. It is however beyond the scope of this study.

Considering that pairwise motif disruption already suggests certain levels of synergistic effects, the higher order combinatorial effect of mutant motifs and their context on expression may be more difficult to understand. To dissect this complexity of the mutant combinations, we used a linear regression model to check how much of the core promoter activity can be correlated with individual effects. To our surprise, we found that the expression changes caused by single mutations of sequence motifs joined in a linear fashion can predict to an important extent the output of the free mutant combinations. Hence, promoter expression levels of mixed and combined motifs can largely be explained by simple linear addition of their individual contributions. We also extended the sequence features from the motifs alone to larger sequence blocks that contain motifs together with their context. Here too, we found that a linear model describes the expression of these inter‐architectural block combinations well. A linear combination of individual sequence features like the motifs or wider sequence blocks including their context sequences can account for two‐thirds of the variance in expression levels, as regulated by the core promoter. To unravel the nonlinear interactions, more data and detailed models would however be necessary.

The ecdysone responsiveness highly depends on the core promoter architecture. This developmental stimulus functions more strongly on developmental core promoters. There is a generally negative correlation between the ecdysone responsiveness and the basal expression level. Our strongest promoters can barely be induced by ecdysone. The higher the expression level, the more difficult it is to further boost the signal, hinting at the saturation of the promoter expression. This effect is stronger for constitutive core promoters, showing their less efficient activation. The disruption of INR in developmental core promoters can lead to a reduction in the ecdysone responsiveness, which is consistent with what was reported in a previous study in *Spodoptera frugiperda* (Jones *et al*, 2012). Taken together, the different sequence motifs composing distinct core promoter architectures can predict their ecdysone responsiveness: Developmental core promoters exhibit a stronger inducibility.

Finally, by investigating the effect of potential nucleosome binding, we observe moderate effects on expression (compared to motif knockouts) driven by these different potential nucleosomal backgrounds. Note that although we checked nucleosomal presence on plasmid for one construct, it is not known if our promoters have native nucleosome occupancy. We however find greater expression variation for housekeeping and ribosomal core promoters than developmental core promoters when changing the TSS nucleosomal sequence downstream the TSS (block 7); this suggests the significance of the genomic +1 nucleosomal sequences for the function of constitutive core promoters.

Our method based on the luciferase assay for assessing promoter activity has however limitations: Different translation efficiency due to the varying 5′UTR between transcripts is not captured, and the assay is blind toward the TSS that is actually being used in the endogenous promoters. These phenomena could lead to promoter activity measurements that do not perfectly reflect the endogenous expression. Additional shortcomings of our technique are that the measurements were performed using episomal plasmids in transiently transfected cells. Although we inserted the genomic ± 1 nucleosome positioning sequences from different genes surrounding the tested core promoter region, they still lack the ability to represent the endogenous chromosomal context and the higher order genomic structure, which might change the basal expression levels as well as the ecdysone inducibilities. Furthermore, our method has a moderate throughput that is lower than most sequencing‐based approaches, and the cloning and colony picking procedures also limit our sequence recovery from the designed oligonucleotides (Materials and Methods).

## Materials and Methods

### *Drosophila melanogaster* core promoter clustering and *de novo* motif search using the *XXmotif* algorithm

In a previous work, we devised the *XXmotif* (eXhaustive evaluation of matriX motifs), a *P*‐value‐based regulatory motif discovery tool using position weight matrices (PWMs) (Luehr *et al*, 2012; Hartmann *et al*, 2013). In brief, we first grouped genes genome‐wide based on experimentally derived features, including expression strengths and variations throughout developmental stages (Graveley *et al*, 2011), Pol II stalling (Zeitlinger *et al*, 2007; Hendrix *et al*, 2008) and TSSs mapping from CAGE data (Ni *et al*, 2010; Hoskins *et al*, 2011). We then applied *XXmotif* for the *de novo* motif search in the core promoter regions of these genes and were able to identify widely known motifs as well as some novel motif candidates with optimized PWMs based on enrichment, localization, and conservation (Hartmann, 2012). The produced gene sets correlate with different core promoter elements (CPEs) architectures.

### TSS cluster width

We used TSS tag data to separate genes with a positionally defined transcription start from genes utilizing several TSSs distributed over a broader genomic region. In a first step, we assigned TSS tags to promoters, by smoothing the TSS tag counts with a rectangular kernel (of width 41) and defining regions above the genomic background frequency as clusters if they were close enough to an annotated promoter. Clusters were defined as continuous regions with a tag distribution higher than the genomic average. For each cluster, the TSS was declared at the position with the most assigned tags. For further analysis, we only used clusters with at least five annotated tags and no other TSS within a range of 150 bps. Furthermore, we only considered clusters with either an annotated gene start within 250 bps upstream of the TSS, have the TSS within an annotated 5′UTR, or contain an annotated *FlyBase* TSS within the cluster. The clustering resulted in 12,061 different TSS clusters for 8502 different genes. To quantify the peakedness of a cluster, we utilized a score calculating the mean absolute deviation from the median (TSS width):

(1) $$\text{TSS}\;\text{width}=\frac{1}{n}\sum_{i=1}^{n}|x_{i}‐\text{median}\left(X\right)|$$

(2) $$\text{MAD}\frac{1}{n}\sum_{i=1}^{n}|x_{i}‐m\left(X\right)|$$

where *n* is the number of tags within the cluster, *x_i_* represents the position of the *i* ^th^ tag, and median(X) is the median tag position within the cluster. In contrast to the SI score—which has a clear bias toward lower scores if the TSS cluster has many tags—the MAD score is independent of the cluster size.

### Gene sets

We selected overlapping gene sets depending on TSS cluster width (described above), inducibility of gene expression (MAD expression), minimum gene expression, maximum gene expression, gene expression in embryo, larva, or female, and gene expression in adult (Fig EV2B). If possible, the minimum of the distribution was chosen as thresholds; otherwise, the highest and lowest 10% quantiles were used to derive gene sets with special behaviors. As an exception, we divided the tail of the MAD expression distribution into two overlapping classes: the “high” class consists of the 10% genes with highest MAD expression, whereas the “medhigh” class consists of the top 40% of genes. In addition to these 18 sets, we adopted a set of genes classified as stalled by Hendrix *et al* (2008).

### Identification of core promoter elements

To examine whether specific CPEs are enriched within the gene sets (Fig EV2 and Appendix Table S1), we performed a *de novo* motif search for each set separately by applying the *XXmotif* (eXhaustive evaluation of matriX motifs) algorithm (Luehr *et al*, 2012; Hartmann *et al*, 2013). We searched in the core promoter region: −100 bp to +50 bp around the TSS. Furthermore, by aligning the sequences of the four most related *Drosophila* species, we exploited another feature of *XXmotif*. If CPE was found in more than one set, we selected the version with the lowest reported E‐value as the representative for further analysis.

To assign binding sites for every motif PWM in every gene set we used two criteria: (i) If *XXmotif* identifies a significant localization, the binding site has to lie within the region of enrichment. (ii) The match score (how well the PWM matches a binding site) has to exceed a score threshold specific for the motif. To determine this minimal threshold, we optimized the mutual information between the motif and each gene set, which corresponds to an optimization of the TF concentration. The gene set with the highest mutual information (and positive correlation) to the motif is given in column “*Gene set*” of Fig EV2A.

### Optimization of minimal score thresholds

To determine the minimal log‐odd score of a PWM indicating the presence of a motif, we calculated the mutual information between the motif and all gene sets to which the PWM has a positive correlation given all minimal score thresholds ranging from −15 to 30 with step size 0.1. For each motif, we chose the minimal score threshold leading to the highest mutual information in any gene set.

### Conservation scores

For each motif, conservation scores were calculated on the assigned binding sites for all 12,061 *D. melanogaster* core promoter sequences. Alignments were generated using the UCSC 14‐way multiple sequence alignments (dm3). The conservation score Scons(X) for each of the 11 *Drosophila* species X was calculated as follows:

(3) $$S_{\mathrm{cons}}\left(X\right)=\frac{1}{N}\sum_{i=1}^{N}\frac{B_{i}\left(X\right)‐S\left(X\right)}{B_{i}\left(X\right)}$$

where S(X) is the average log‐odds score difference between *D. melanogaster* and species X from the alignment, and B_i_(X) is the expected average log‐odds score difference from a null distribution based on the *i* ^th^ of *N* sets of sampled binding sites. Each binding site is sampled from a position‐specific substitution matrix learned on the alignment to species X at the respective position +/− 10 bps. We used *N* = 50 for the analysis.

### Core promoter elements allow for the prediction of gene properties

To analyze the influence of TSS cluster width, expression in developmental stages, and stalling index on the enrichment of CPEs, we ordered all genes depending on each property (Fig EV3 and Appendix Fig S2) and calculated *Z*‐scores for the enrichment of every CPE within bins of 50 genes. Groups of CPEs show transitions between correlation and anticorrelation at specific scores. Smaller CPE groups are enriched in stalled genes: INR, DPE, GAGA (B), genes with high expression in all developmental stages: Ohler6, Ohler7, INR3, RDPE (C), genes with high expression in one or more developmental stage: TATA‐box, ATGAA, INR3, RDPE (D), and the most regulated genes: TATA‐box, ATGAA (E). Correlating each CPE with each gene set provides an overview of core promoter architectures in *D. melanogaster* (F).

### Synthetic promoters design

#### Building blocks

We designed synthetic promoter constructs by dividing the promoter region into 7 building blocks (Fig 1A and B): *block 3‐6* (131 bp in length) was the motif‐rich core promoter region (−80 to +50 bp around the TSS) with native and mutated sequences from different core promoter architectures to investigate the effects of sequence motifs; *block 2* (73 bp) represented the EcREs, which contained the binding sites for the ecdysone receptors to recruit the steroid hormone ecdysone for transcriptional activation; *block 1* (239 bp) and *block 7* (240 bp) were used for testing the influence of nucleosomal sequence context. The entire lengths for the designed synthetic promoters inserted into the vector backbones were 703 bp with *block 7* and 459 bp without *block 7*.

#### Nucleosomal context (block 1 and block 7)

After MNase digestion of chromatin, genome‐wide nucleosome maps were generated. 12 gene promoters were selected according to their pattern of nucleosome positioning and occupancy relative to their TSS (especially ± 1 nucleosomes) and pairs of *block 1* and *block 7* sequences representing different potential ± 1 nucleosome patterns were selected (sequences in Appendix Table S4 and S5). The block 1 and 7 sequences were synthesized either by PCR amplification from the genomic DNA (isolated from sequenced fly strain, stock number 2057 in Bloomington *Drosophila* Stock Center) or by oligo synthesis from Life Technologies (for HindIII recognition sites mutated and ATGs mutated sequences). All synthesized sequences of *block 1*s and *block 7*s contained BsaI sites and assembly overhangs, and they were stored in TOPO vectors (Zero Blunt TOPO PCR Cloning Kit, Invitrogen). In the experiments, we tested *block 1* and *block 7* in pairs with all 19 native core promoter *blocks 3‐6*, five out of which were then selected to combine with all free combinations of *block 1* and *block 7* (one from each architecture with activities covering the entire dynamic range: CG15674 (motif‐less), Mec2 (Ar.1), Mtk (Ar.2), CG17712 (Ar.3), RpL23 (Ar.4). We also constructed synthetic promoters containing only *block 1* (without *block 7*) for these five wild‐type *blocks 3‐6*. One pair of *block 1*.11 and *block 7*.11 was selected based on its high expression level and used as the fixed nucleosomal sequence context for highly mutated blocks 3‐6.

#### Ecdysone receptor binding site (block 2)

The *block 2,* which contained three EcR/USP heterodimer binding sites with 17 bp spacers in between, was synthesized by oligo annealing (5′‐gcGGTCTCAATGAagttcattgacctagtgag aattcacagcgagttcattgacctactcaaggcatacatgaagttcattgacctGGATTGAGACCgc‐3′; lowercase with underline: EcR/USP binding sites from JASPAR database (Khan *et al*, 2018); italic: assembly overhangs; uppercase with underline: BsaI restriction sites).

#### Selection of the native core promoter set (blocks 3‐6)

From the four core promoter architectures (including two subclasses Ar.3.1 and Ar.3.2 of the housekeeping Ar.3; Fig EV3C) and one additional architecture without having any known motif termed motif‐less promoters, we chose 2–4 native core promoters each with high or intermediate to low expressions according to their maximum expression levels in *S2* cells (previous RNA‐seq data generated by our group; position −80 to +50 relative to TSS which was set to be position 0; block 3: −80 to −35, block 4: −34 to −10, block 5: −9 to +8, block 6: +9 to +50). In total, we thus selected 19 wild‐type core promoters, some of which have mixed architectures due to different motifs co‐occurrence (Fig EV4; their 131 nt sequences listed in Appendix Table S2). The annotation of core promoter motifs in these sequences was carried out by motif search using *XXmotif* according to previously defined motif features (summarized in Appendix Table S1). In addition, we mutated TSS downstream ATGs in the original sequences to TAGs to remove unwanted translation start sites.

#### Mutation with different strengths of motifs

Various kinds of mutations were designed for these native core promoters, including mutations for motifs within each core promoter (main mutations shown in Figs 3A and 6A) and block‐wise mutations between different core promoters (Figs 6C and E). We scanned every designed sequence with our PWMs to check if the mutants we created would lead to undesirable side mutational effects, for example, the creation of new motifs/TF binding sites or disruption of other motifs (as those unintended mutations would cause expression changes).

#### Knockout of motifs

For knocking out individual motifs in the 16 selected native core promoters (excluding three motif‐less promoter sequences), two versions of sequences were used as substitutions: random sequences and background sequences. Random sequences were generated by sampling sequences having the same length with the target motifs and checking with the *XXmotif* derived motif list to make sure no known core promoter motif inside (whose PWM scores lower than the threshold, threshold score of each motif listed in Appendix Table S1). These random sequences were not fixed for the same motif in different promoters (every random sequence was different). Background sequence was a fixed sequence from the identical position of the target motif in the motif‐less core promoter CG15674 (due to the various positions of a certain motif in different promoters, the background sequence might vary). Knockout of all motifs in a given promoter was designed in the same way, using both random and background sequences. Pairwise knockout of motifs only used random sequences for replacing two original motifs at the same time.

#### Consensus replacement of motifs

For the nine main motifs INR, MTEDPE, TATA‐Box, INR2, Ohler6, DRE, Ohler7, TCT, and RDPE, we replaced them in native core promoters with the consensus sequences derived from *XXmotif*. Additionally, these consensus sequences were also inserted into the three motif‐less core promoters with their start positions at the peaks of the native motif distribution (motif distribution shown in the column “Distribution” of Fig EV2A).

#### Replacing native motifs with their alternatives of various strengths

Alternatives with different PWM scores for the nine main motifs mentioned above were randomly generated, making sure that their scores either evenly covered several score bins below the threshold and the maximum.

#### Point mutation of motifs

For the 12 motifs INR, MTEDPE, CGpal, TATA‐Box, INR2, Ohler6, DRE, Ohler7, TCT, RDPE, TTGTT, and TTGTTrev, we designed all possible single base‐pair mutations around the motif’s consensus sequence. This was done for each motif within a selected native core promoter configuration: INR in Mec2; MTEDPE and CGpal in Cas; TATA‐Box in CG8157; INR2, Ohler6 and TTGTTrev in Thoc6; DRE, Ohler7 and TTGTT in RpL36A; TCT and RDPE in RpL5. Additionally, INR, DRE, Ohler7, and TCT were also checked in an motif‐less context sequence obtained from CG10915, with the insertion of each consensus sequence.

#### Substitution of motifs

The target motif was firstly knocked out with a random sequence. The motif sequence for substitution was also randomly sampled with a PWM score above the threshold and was always the same for each motif. Three combinations were tested here: INR (7 nt)‐INR2 (15 nt)‐Ohler7 (13 nt)‐TCT (11 nt); TATA‐Box (10 nt)‐Ohler6 (10 nt)‐DRE (10 nt); MTEDPE (17 nt)‐RDPE (17 nt). For INR‐like motifs with various lengths, the supposed position for TSS (3^rd^ position in INR, 10^th^ in INR2, 5th in Ohler7 and 6th in TCT; based on the motif start positions listed in Appendix Table S1) was aligned when replacing the sequence.

#### Positional shift of motifs

Positional shifts were designed for individual motifs and all motifs together in a given core promoter, as well as for sequence context surrounding motifs (motifs kept at the original positions). For strictly positioned motifs like INR, MTEDPE, and TATA‐Box, shifts of 1, 2, 3, 5, 10 bp either downstream or upstream were applied; for less well‐positioned housekeeping core promoter motifs like DRE and Ohler7, larger distances were chosen (±1, ±3, ±5, ±10, ±20 bp).

#### Other combinatorial mutations

Further combinatorial mutations were designed to the motif‐rich core region, including free combinations of mutations both within defined core promoter architectures and between them (termed as intra‐architectural motif‐wise and inter‐architectural block‐wise combinatorial mutations). In addition, context sequences surrounding the motifs were also tested by exchanging them between different core promoters.

For testing these combinatorial mutations, one representative core promoter sequence from each architecture with motifs located within distinct block regions was selected: Cas (Ar.1), CG8157 (Ar.2), Thoc6 (Ar.3.1), RpL36AN (Ar.3.2), and RpL5 (Ar.4). The synthetic promoter RpL36AN was derived from the native RpL36A (Ar.3.2) shifting the TSS position 16 nt upstream in order to shift all motifs into the blocks where they occur most frequently, based on the distributions generated by *XXmotif*. In addition to the five core promoter sequences tested systematically in all three types of combinatorial mutations, several other native sequences were also included (MED4 for intra‐architectural mutations; Mtk and Cpr47Eg for inter‐architectural mutations; CG10915 and CG15674 for context exchange).

#### Intra‐architectural motif‐wise combinatorial mutations

Multiple motif‐wise mutations for altering both motif strength and motif position within a core promoter sequence were performed here. The MED4 (Ar.3.1) was selected because of its strong native activity level, which ensures a relatively strong luminescence signal even after severe combinatorial mutations. Single mutations (knockouts, replacing by the consensus or alternatives with different PWM scores and positional shifts) for individual motifs in each core promoter were re‐designed in the same way as described before but kept the same in all intra‐architectural combinatorial mutations. Shifts of motifs were made within shorter ranges (±1 bp or ±5 bp).

#### Inter‐architectural block‐wise combinatorial mutations

We applied block‐wise swaps between different core promoter sequences here. Two additional sequences Mtk and Cpr47Eg were included to provide extra block patterns. In detail, block pieces from 7 native core promoters were selected and freely combined to construct the synthetic *block 3‐6* regions: four block 3s from CG8157 (background sequence of Ar.2), RpL36AN (background sequence of Ar.3.2, BP), RpL5 (Ohler6 existed), Cpr47Eg (CGpal existed); five block 4s from Cas, CG8157, Thoc6, RpL36AN, RpL5; four block 5s from CG8157, Thoc6, RpL36AN, RpL5; six block 6s from Cas, CG8157, Thoc6, RpL36AN, RpL5, Mtk (background sequences of Ar.2).

#### Context exchange

All motifs in a given core promoter were knocked out using the same sequences designed for single knockouts in intra‐architectural combinatorial mutations. All motifs from other core promoter sequences were inserted into this context at their native positions (Fig 6C). Two motif‐less core promoter contexts were also included: CG10915 and CG15674 (Adams *et al,* 2000).

### Experimental setup and procedures

#### Reporter and control plasmids for dual luciferase assay

A two‐vector system was used in the experiments. Firefly reporter vector backbone was derived from a commercial vector pGL4.13 with luc2 firefly gene (Promega). HindIII and BglII restriction enzymes (Khan *et al,* 2018) were used to cut out the SV40 early enhancer/promoter region in the original plasmid. To insert BsaI sites and 4 bp overhangs, two dsDNAs with HindIII and BglII sites were generated by oligo annealing: for the constructs containing a *block 7* (sequences listed in Appendix Table S5), the following sequence was used: gcagatctgcGAACTGAGACCgtcgacgcaaggcctgcaattaatgcagcggccgatcggcatatgGGTCTCA CCACcaaagcttcg (only forward sequence; BglII or HindIII restriction sites: lowercase with underline; overhangs: italic; BsaI restriction sites: uppercase with underline); the sequence used for the constructs without *block 7* was: gcagatctgcGAACTGAGACCgtcgacgcaaggcctgca attaatgcagcggccgatcggcatatgGGTCTCATCTGcaaagcttcg. After enzyme digestion and gel purification (QIAquick Gel Extraction Kit, Qiagen) of both vector and inserted DNAs, ligation (Rapid DNA Ligation Kit, Roche) was performed to obtain the two final vector backbones (4,299 bp), named as BB0 for the constructs without *block 7* and BB1 for the constructs containing a *block 7*.

Renilla control plasmid (3,630 bp) was derived from another commercial vector pGL4.70 with the hRluc renilla gene (Promega) by insertion of a moderate‐strength P transposase (pTran) promoter between NheI and XhoI sites. The pTran promoter was cloned from a vector created in the lab pKF1 (derived from a P‐element sequence, position 34–141 according to (O’Hare & Rubin, 1983) using primers: 5′‐GCGCTAGCAGCCGAAGCTTACCGAAGTATAC‐3′, 5′‐GCCTCGAGCCACGTAAGGGTTAATGTTTTC‐3′ (underlines: NheI and XhoI restriction sites).

Several inter‐plate controls were used in the experiments. The negative control was one commercial vector pUC19 (Khan *et al,* 2018). There were two positive controls: One was pGL4.10 vector (Promega, with luc2 firefly gene) with pTran promoter inserted between NheI and XhoI sites, termed as pUG9, whose signal was used in data normalization procedure (4,350 bp); the other one was a synthetic test plasmid pZQ3(4,691 bp) with moderate promoter activity which contains our firefly reporter backbone BB0 and blocks 1–6 for ecdysone inducibility check: *Block 1*.3 (all *block 1* sequences listed in Appendix Table S4) + *Block 2* (sequence indicated above) + *Block 3‐6* with INR and DPE motifs (sequence: GGCTCCGAATTCGCCCTTTTCCCAGGGCGGCAGAGGCAAAAATTTGCCGA TCCCAGAGCCAGCCGACTCATTCAAAGCTCCGACTTCGTTGCGTGCACACAGAGTCTCAAGGGCGACCCAGCTTT).

#### Cloning

For carrying out our large‐scale systematic analysis, we developed a high‐throughput experimental pipeline using automated robotic systems (Fig EV1). After preparation of each construct block (*block 1* and *block 7*: PCR amplification from the fly genome or oligo synthesis; *block 2*: oligo annealing; *block 3‐6*: PCR amplification from the synthetic library (Agilent Technologies) according to mutation families), Golden Gate cloning (BsaI cloning) was applied to join them with the vector backbones sequentially. Then, the newly synthesized reporter plasmids were transformed into electrocompetent *E. coli*, followed by plating bacteria on one‐well plates, this way facilitating automated colonies picking using the robotic workstation. After bacterial growth in 48‐well plates, we rearranged them into 96‐well LB plates and prepared the library for next‐generation sequencing with two‐step PCR using nested barcode primers. Based on the sequencing results, replicates and bad clones were screened out and DNAs from confirmed positive clones were isolated. These firefly reporter plasmids containing all the distinct promoters were then used for transient co‐transfection into *D. melanogaster* S2 cells together with the renilla control plasmid in 96‐well plates. After overnight incubation, cells were treated with ecdysone for another 2 h. Four cell culture 96‐well plates were pulled into 384‐well plates for the final dual luciferase assay readout in order to use less substrate for the luciferase assays.

#### Automation

We used two independent robot platforms with a similar basic configuration of pipettor systems (Biomek NXP automated workstations with Multichannel‐96 and Span‐8 pipetting model, Beckman Coulter). Additional instruments were integrated with the original workstations including incubators (Incubator Shaker DWP, Inheco), thermocyclers (Biometra TRobot, Analytik Jena), barcode printer (Microplate Print & Apply, Beckman Coulter), barcode reader (Compact Laser Barcode Scanner, Omron Microscan), plate reader (SpectraMax Paradigm Multi‐Mode Microplate Reader, Molecular Devices), and plate sealer (Wasp, Kbiosystems). They were designed for maximum flexibility to perform many different experiments. Specifically, one system is dedicated to bacterial experiments, mainly the cloning‐related work: colony picking, colony PCR, hitpicking for positive clones, DNA isolation, and concentration measurement. The colony picking is a customized feature of this robotic configuration. The other system is dedicated to *Drosophila* cell assays: transient co‐transfection, ecdysone treatment, and luciferase assay readout. In addition, an electronic multichannel pipette on an assistant robot (VIAFLO Electronic Multichannel Pipette + ASSIST Pipetting Robot, INTEGRA) was used for automated cell plating into 96‐well plates.

#### Synthetic library amplification

*Block 3‐6*s for the motif‐rich core regions of our synthetic promoter constructs were amplified from a library synthesized by Agilent Technologies (LeProust *et al*, 2010) together with BsaI sites, relevant overhangs and unique primer sequences referred to distinct mutation families, in total 3,826 fully designed oligonucleotides (in total ~200 nt long for each sequence). The entire oligo pool (lyophilized, 10 pmol) was dissolved in 100 μl elution buffer (Qiagen) and shaken at room temperature (RT) for 30 min at 450 rpm and 10 min at 950 rpm. 0.5 μl of library DNA was used to amplify the specific sequence family (native sequences or one of distinct mutation families) in a 20 μl PCR, which also included 1.25 μl of both forward and reverse 10 μM customized primers, 4 μl 5× Herculase II reaction buffer, 0.5 μl 10 mM dNTP mix, and 0.5 μl Herculase II fusion DNA polymerase (Agilent Technologies). PCR parameters were as follows: 98°C for 3 min; followed by 15 cycles of 98°C for 80 s, 54°C for 30 s, 72°C for 40 s; 72°C for 10 min. Each PCR was purified with the QIAquick PCR purification kit (Qiagen) according to the manufacturer’s instructions and eluted in 30 μl of nuclease‐free water (Qiagen).

#### Golden Gate cloning and transformation

BsaI restriction enzyme (10,000 U/ml, NEB) and T4 DNA ligase (3 U/μl, Promega) were applied to assemble all of the synthetic promoter blocks sequentially and simultaneously into the firefly reporter vector backbone in a one‐pot reaction. For each 20 μl reaction, DNA master mix contained equimolar amount (80 fmol) of each part: *block 1* in TOPO vector (3,784 bp), *block 2* (99 bp), *block 3‐6* (200 bp), *block 7* in TOPO vector (3,785 bp, if needed) and backbone (4,299 bp) together with 2 μl BsaI, 2 μl T4 DNA ligase and 2 μl 10× ligase buffer. The cloning protocol included 3 steps: (1) 20 cycles of 37°C for 2 min, 16°C for 3 min; followed by 50°C for 5 min and 80°C for 5 min; (2) After adding 1 μl BsaI, 1 μl T4 DNA ligase, 1 μl 10 mM ATP: 16°C for 20 min; 15 cycles of 37°C for 2 min, 16°C for 3 min; followed by 50°C for 5 min and 80°C for 5 min; (3) After adding again 1 μl BsaI: 37°C for 10 min, 50°C for 20 min, 80°C for 10 min and ramp down to 25°C by 0.1°C/s. After BsaI cloning, 2 µl of the reaction mix was transformed into 40 µl of electrocompetent TOP10 *E. coli* cells (homemade). After electroporation (1.8 kV for 0.1 cm cuvettes, Gene Pulser, Bio‐Rad) and 1 ml SOC medium (homemade) addition, cells were incubated for 1 h at 37°C (shaking at 450 rpm) and plated 100 µl onto prewarmed 1‐well LB‐agar plates supplemented with 100 µg/ml ampicillin.

#### Colony picking

After overnight incubation at 37°C, the 1‐well plates were ready for colony picking. Span‐8 pipetting system on the robot was used to automatically pick individual colonies (customized protocol) into two 48‐well plates (Riplate SW 48, 5 ml, Riplate) with 2.4 ml LB‐ampicillin medium (ampicillin concentration: 120 µg/ml). The plates were incubated for 16 h at 37°C (horizontally shaking at 180 rpm) and rearranged into one 96‐well plate (MegaBlock 96 Well, 2.2 ml, Sarstedt). 110 µl/well of bacteria was used to create glycerol stock plate (Round 96 Well Storage Plates, U‐bottom, 330 µl, 4titude) and 30 µl/well for PCR plate (FrameStar 96 Well Skirted PCR Plate, 4titude) ready for sequencing library preparation. Since in the previous cloning step, the sequences from the same mutation family were all mixed together, it is necessary to recover the individual sequences, but is unfortunately technically impossible to recover all of them. Sequence separation occurs at the colony picking step and the number of different recovered sequences will increase exponentially with the number of picked colonies required. In addition, we observed that about 40% of the recovered sequences presented defects, arising from the synthesis procedure, which thus increase the number of picked colonies necessary accordingly. Thus, it would become pricely and timely prohibitive to fully recover all the designed sequences. However, as our analysis method can accommodate missing mutational sequences, we decided that picking and preparing glycerol stocks for 4–5 times more colonies than the number of designed sequences leading to 80% of recovered sequence constitutes a satisfying compromise without scarifying data quality. This way, we were able to recover in total more than 3000 of the designed sequences.

#### Next‐generation sequencing of the picked clones

Two‐step PCR with nested barcode primers was implemented for library preparation. The forward and reverse primers for 1st PCR targeted the sequences in *block 2* and vector backbone respectively with specific barcodes (*block 1* was always known in the BsaI cloning procedure). 2 µl/well of bacteria were used to set up a 25 µl PCR containing 1 µl homemade Taq/Pfu polymerase mix, 2.5 µl primer mix (forward and reverse each 500 nM), 1 µl 25 mM MgCl2, 2.5 µl 10× buffer, 1 µl 2.5 mM dNTP. 96‐well plate PCRs were performed in the thermocyclers integrated on the robot (96°C for 7 min; 3 cycles of 94°C for 30 s, 68°C for 30 s, 72°C for 2 min; followed by 3 cycles of 94°C for 30 s, 64°C for 30 s, 72°C for 2 min; 17 cycles of 94°C for 30 s, 56°C for 30 s, 72°C for 2 min). 5 µl/well of the product from each 1st PCR plate was pooled into one specific well of the collection plate (Deepwell plate 96/500 µl, Eppendorf; each well containing all 96 samples from one 1st PCR plate). 3.5 µl/well was then used as template for 2^nd^ PCR in a 50 µl reaction together with 0.5 µl Herculase II fusion DNA polymerase (Agilent Technologies), 10 µl 5× Herculase II reaction buffer, 1.25 μl 10 mM dNTP mix and 5 μl each of Illumina index primers (Nextera XT Index Kit v2, Index 1 (i7) Adapters and Index 2 (i5) Adapters, Illumina). So each well of 2nd PCR plate (each 1st PCR plate samples) got a unique pair of index adapters. PCR was performed as the same protocol for 1st PCR. The final products were pooled and purified using Agencourt AMPure XP magnetic beads (Beckman Coulter) according to the manufacturer’s instructions. Next‐generation sequencing (Illumina HiSeq1500) was performed by the LAFUGA sequencing facility at the Gene Center LMU Munich.

#### Hitpicking and DNA isolation

Automated hitpicking of positive clones from glycerol stock plates was carried out using our robotic system. 75 μl of the samples in the original plates were reformatted into the final 96‐well glycerol stock plates (Round 96 Well Storage Plates, U‐bottom, 330 µl, 4titude) and 20 μl were used for reinoculation in 48‐well plates (Riplate SW 48, 5 ml, Riplate) with 2.4 ml LB‐ampicillin medium (ampicillin concentration: 120 µg/ml). The plates were incubated for 17 h at 37°C (horizontally shaking at 180 rpm) and rearranged into one 96‐well plate (1.2 ml/well; MegaBlock 96 Well, 2.2 ml, Sarstedt). After centrifugation at 5,000 *g* for 15 min, the supernatant was discarded and cell pellets were stored at −20°C ready for DNA isolation. Minipreps in 96‐well plate format was performed with Wizard MagneSil TfxTM System (Promega) on the robotic workstation according to the manufacturer’s instructions. DNA concentrations were measured using the SpectraMax Microplate Reader integrated on the robot (5 μl DNA samples on the SpectraDrop Micro‐Volume Microplates, Molecular Devices).

#### Cell culture

*Drosophila melanogaster* S2 cells were firstly thawed at passage 12 with Schneider's *Drosophila* Medium (Bio&Sell, supplemented with 10% FBS (Fetal Bovine Serum, Biochrom)) and later cultivated in Express Five SFM medium (protein‐free and serum‐free, Invitrogen). One bottle of the Express Five medium (1 l) was supplemented with 90 ml of L‐Glutamine (200 mM, Invitrogen). During cultivation, cells were grown at 25°C without CO2 in tissue culture flasks (75 cm^2^, Corning) and were split into fresh flasks when 90% confluent. The cells in passage 18 were seeded into 96‐well plates (Falcon 96 Well Tissue Culture Plates, Corning) with 40,000 cells per well in 100 µl using an electronic multichannel pipette VIAFLO (1,250 µl, INTEGRA) on a pipetting robot ASSIST (INTEGRA). The cells 24 h growth rate and viability were monitored in the culture dishes (in duplicate; 100 mm, Corning) with 12 × 106 cells in 14 ml medium. Cell counting and assessment of cell viability were performed using the Cell Counter and Analyzer System (CASY, Roche).

#### Transient co‐transfection

24 h after cell plating, transient co‐transfection on the robot system was performed using FuGENE® HD Transfection Reagent (Promega) according to the manufacturer’s protocol. To avoid multiple freeze–thaw processes, the renilla control plasmid and three inter‐plate control plasmids (pUC19, pUG9, pZQ3) were aliquoted in PCR strips sufficient for one transfection experiment. The isolated reporter plasmids and inter‐plate control plasmids were transferred into 96‐well master mix plates according to the transfection plate layout together with renilla control plasmids (except for untreated cells (UTCs), reporter plasmid or inter‐plate control plasmid renilla control plasmid ratio = 8:1, total DNA amount 0.945 µg per well). Wells indicated with green shadows were filled with various reporter plasmids containing synthetic promoter constructs to be tested. 2.3 µl/well FuGENE® HD Transfection Reagent was added and the FuGENE® HD‐DNA mixture was incubated for 5 min at RT (FuGENE® HD : DNA ratio ~2.4:1). 10 µl FuGENE® HD‐DNA mixture was then added per well into 96‐well cell culture plates. The transient co‐transfections were performed in duplicates for cells with and without ecdysone treatment.

#### Ecdysone treatment

Cells were incubated for 22 h after transfection, followed by 2 h of ecdysone treatment (final ecdysone concentration: 10 µM; 20‐Hydroxyecdysone, Sigma‐Aldrich). The other replicate transfected cell plate was treated with the same volume (10 µl/well) of cell culture medium (Express Five medium supplemented with l‐glutamine) and incubated for 2 h.

#### Dual luciferase assay

40 µl/well of the mediums was removed from each cell culture plate and 20 µl/well of cells were transferred into the final readout plates. For each measurement, samples from four 96‐well cell culture plates were joined into two 384‐well plates (one for firefly luminescence measurement, the other for renilla luminescence measurement; AlphaPlate‐384, PerkinElmer). ONE‐GloTM Luciferase Assay System (Promega) and Renilla‐Glo® Luciferase Assay System (Promega) were used, respectively (reagent amount: 20 µl/well). There was a common crosstalk issue between two adjacent wells caused by the bleed‐through of the stronger luminescence signal to the other. In the optimized protocol, firefly luminescence signal was measured twice with strong signals (> 2 × 10^5^ RLU, relative light unit) identified in the first measurement and removed before the second measurement (samples were pipetted out and a highly concentrated dye (1 mM Nile Blue A, Sigma‐Aldrich) that quenches the luminescence signal was added instead). This experimental procedure was designed to solve the crosstalk issue between adjacent wells that we observed for strong promoters (Fig EV1). Bioluminescence signals were measured using a SpectraMax Microplate Reader (Molecular Devices).

#### Validation experiments in developing *Drosophila melanogaster* embryos

Cloning, generation of transgenic fly stocks and embryo imaging was performed as previously described in (Ceolin *et al*, 2020). Briefly, the 9 constructs created in this study were generated by replacing the DSCP promoter in the plasmid *hb_ant‐mNeonRep* from (Ceolin *et al*, 2020). This plasmid contains a codon optimized version of the mNeonGreen fluorescent protein, fused to three nuclear localization signal and also includes the IVS + Syn21 translational enhancer sequences at the 5′UTR and the p10 terminator sequence at the 3′UTR (Pfeiffer *et al*, 2012).

All reporter plasmids were integrated in the same genomic site using PhiC31 integrase (Pfeiffer *et al*, 2010). Imaging has been performed on embryos obtained from homozygous fly stocks, verified by fly PCR and sequencing of the PCR products. Prior to imaging, embryos were collected, dechorionated in 50% bleach, immersed in halocarbon oil, and mounted between a microscope coverslip and a semipermeable membrane. Imaging was performed using a Zeiss LSM710 confocal microscope equipped with a 40× 1.2NA water immersion objective. mNeonGreen fluorescence was excited with a 514 nm laser, with a power of 50 µW at the back focal plane of the objective. Each acquisition consisted of a time series of two tiled stacks of three images each, with a z‐resolution of 7.5 µm and a pixel size of 1.1 µm, acquired around the middle section of the embryo. The time resolution was set to 60s per stack. For the final analysis, only one time point was used (5 ± 1 min before the embryo gastrulation) and the temporal information was only used to stage the embryos.

Confocal stacks of embryos were processed using the *Definiens XD 2.0* software package (Munich, Germany) to segment the images and quantify the fluorescence intensity in the cortical region of the embryo, where the nuclei are located, as described in (Ceolin *et al*, 2020). Finally, the fluorescence intensity from multiple nuclei was pooled in bins corresponding to 2% of the egg length and averaged.

### Data analysis

#### Reads mapping for the sequencing results of the picked clones

Sequencing reads were demultiplexed based on the Illumina indexes and the designed barcodes in our customized primers. The most enriched sequence (at least 3‐fold enrichment against the second most frequent sequence) for each sample was used and trimmed to match the target region of our synthetic promoter construct (part of *block 2*, blocks 3‐6 and *block 7*). The trimmed reads were mapped to our designed library using the pairwise alignment method.

#### Data preprocessing and normalization

For each plate, firefly luciferase expression values (*FF*) of each tested samples were normalized to their renilla luciferase values (*REN*) as well as *FF* values of the inter‐plate controls. The 1^st^ firefly measurements (*FF1*) were used as the readout values for samples with strong promoters (*FF1* > 2 × 10^5^ RLU) and the 2^nd^ firefly measurements (*FF2*, signal degradation corrected) were used for other weaker samples.

Background value (*BG*) was calculated as the arithmetic mean of negative control signals (pUC19 and UTCs) got from 2^nd^ firefly measurements (avoiding the potential crosstalk issue; Equation 1). Normalized value of positive control pUG9 (*Norm_pUG9_*) was defined as the arithmetic mean of its *FF1* signals with *BG* subtracted divided by its *REN* signals (Equation 2).

(1) $$BG_{FF2}=mean\left(pUC19_{FF2}+UTC_{FF2}\right)$$

(2) $$Norm_{pUG9}=mean\left(\frac{pUG9_{FF1}‐BG_{FF2}}{pUG9_{\mathrm{REN}}}\right)$$

The final normalized luciferase expression value for each tested sample (*x_i_*) was calculated as Equation 3: its *FF_i_* signal (*FF1* for strong promoters and *FF2* for others) with *BG* subtracted was firstly normalized to its *REN_i_* signal and then to the normalized control *Norm_pUG9_*; the value was then log_2_‐transformed. This value was used as the estimate of the corresponding synthetic promoter activity.

(3) $$\begin{array}{c} FF_{i}=\left\{\begin{array}{c} FF1_{i},ifFF1_{i}\leq 2\times 10^{5}RLU\\ FF2_{i},ifFF1_{i}>2\times 10^{5}RLU \end{array}\right.\\ x_{i}=\log_{2}\left[\frac{1}{Norm_{pUG9}}\times \left(\frac{FF_{i}‐BG_{FF2}}{REN_{i}}\right)\right] \end{array}$$

#### Outlier identification and filtering

We firstly filtered out samples with outlier renilla signals that we found out to be too high or too low to provide an accurate data normalization (*REN* > 10,000 RLU or *REN* < 300 RLU, respectively) and then calculated the median and standard deviation (SD) for normalized luciferase signals of each promoter construct *x* (> 88% with at least three replicates for both with and without ecdysone stimulation). The score used for defining outliers was calculated as:

(4) $$\text{score}=\frac{x_{i}‐\text{median}\left(x\right)}{\text{median}(SD(X))}$$

Here, *x_i_*, as described above, represented the normalized expression value of *i* ^th^ replicate for construct *x*. *SD(X)* denoted all SDs for entire synthetic promoter construct library *X*. The scores with an absolute value of no < 3 were labeled as outliers and were excluded from further analysis.

## Author contributions

Zhan Qi: Data curation; Formal analysis; Investigation; Visualization; Methodology; Writing—original draft. **Christophe Jung:** Data curation; Formal analysis; Visualization; Methodology; Writing—original draft; Project administration; Writing—review & editing. **Peter Bandilla:** Investigation; Methodology. **Claudia Ludwig:** Investigation. **Mark Heron:** Data curation; Formal analysis. **Anja, Sophie Kiesel:** Formal analysis; Visualization. **Julia Philippou‐Massier:** Investigation; Methodology. **Miroslav Nikolov:** Investigation. **Alessio Renna:** Investigation. **Max Schnepf:** Investigation. **Ulrich Unnerstall:** Conceptualization. **Johannes Söding:** Conceptualization; Project administration; Writing—review & editing. **Ulrike Gaul:** Conceptualization; Funding acquisition; Project administration. **Mariam Museridze:** Investigation. **Stefano Ceolin:** Investigation. **Bettina Mühling:** Investigation. **Nicolas Gompel:** Project administration; Writing—review & editing.

In addition to the CRediT author contributions listed above, the contributions in detail are:

ZQ carried out most of the experimental work and analyzed the data with the help of PB, CL, MN, AR, JP‐M, and MS; MH and ASK carried out the computational analysis and designed the promoter sequences; CJ designed the experiments and analyzed the data; UG, UU, and JS designed the study; MM, SC, BM performed the experimental work for with the *D. melanogaster* embryos. UG, CG, UU, NG, and JS supervised the work; ZQ and CJ wrote the paper with contributions from the other authors.

## Supporting information



Appendix

Expanded View Figures PDF

Dataset EV1

## Data Availability

The DNA sequences and their raw expression measurements are provided for all tested synthetic promoter sequences (Dataset EV1). This study includes no data deposited in external repositories.
