# Supplementary material for: Large‐scale analysis of Drosophila core promoter function using synthetic promoters
Source: Mol Syst Biol. 2022 Feb 14;18(2):e9816. doi: 10.15252/msb.20209816 (PMC8842121; doi:10.15252/msb.20209816)
Supplement: Supplementary file 1 — Appendix [file MSB-18-e9816-s004.docx]

Appendix for:

**LARGE-SCALE ANALYSIS OF DROSOPHILA CORE PROMOTER FUNCTION USING SYNTHETIC PROMOTERS**

Zhan Qi, Christophe Jung, Peter Bandilla, Claudia Ludwig, Mark Heron, Anja Sophie Kiesel, Museridze Mariam, Julia Philippou-Massier, Miroslav Nikolov, Alessio Renna, Max Schnepf, Ulrich Unnerstall, Ceolin Stefano, Bettina Mühlidatang, Nicolas Gompel, Johannes Soeding and Ulrike Gaul

**Appendix Figures**

**Appendix Figure S1**. Conservation of exemplary novel motifs............................................................2

**Appendix Figure S2**. Core promoter motifs correlate to distinct gene properties................................3

**Appendix Figure S3**. The four promoter architectures have distinct dinucleotide/physical property patterns and nucleosome occupancies..................................................................................................4

**Appendix Figure S4.** The effect of nucleosomal context on expression...............................................5

**Appendix Figure S5**. All-motifs and pairwise knockouts, consensus replacement, all-motifs shifts, and motif context exchange....................................................................................................................6

**Appendix Figure S6**. Linear and additive models applied to predict the synthetic promoter activity based on individual motif features, and effects of all-motif shifts and context sequence shifts..............8

**Appendix Tables**

**Appendix Table S1.** Summary of *XXmotif*-annotated core promoter motif features.............................9

**Appendix Table S2.** The sequences for the 19 native core promoters investigated...........................12

**Appendix Table S3**. Number of synthetic promoters designed for the different mutation types.........12

**Appendix Table S4.** The sequences of block 1s.................................................................................14

**Appendix Table S5.** The sequences of block 7s.................................................................................16

**Appendix Table S6.** Coefficients of the linear regression model for the inter-architectural block-wise combinatorial mutations........................................................................................................................17


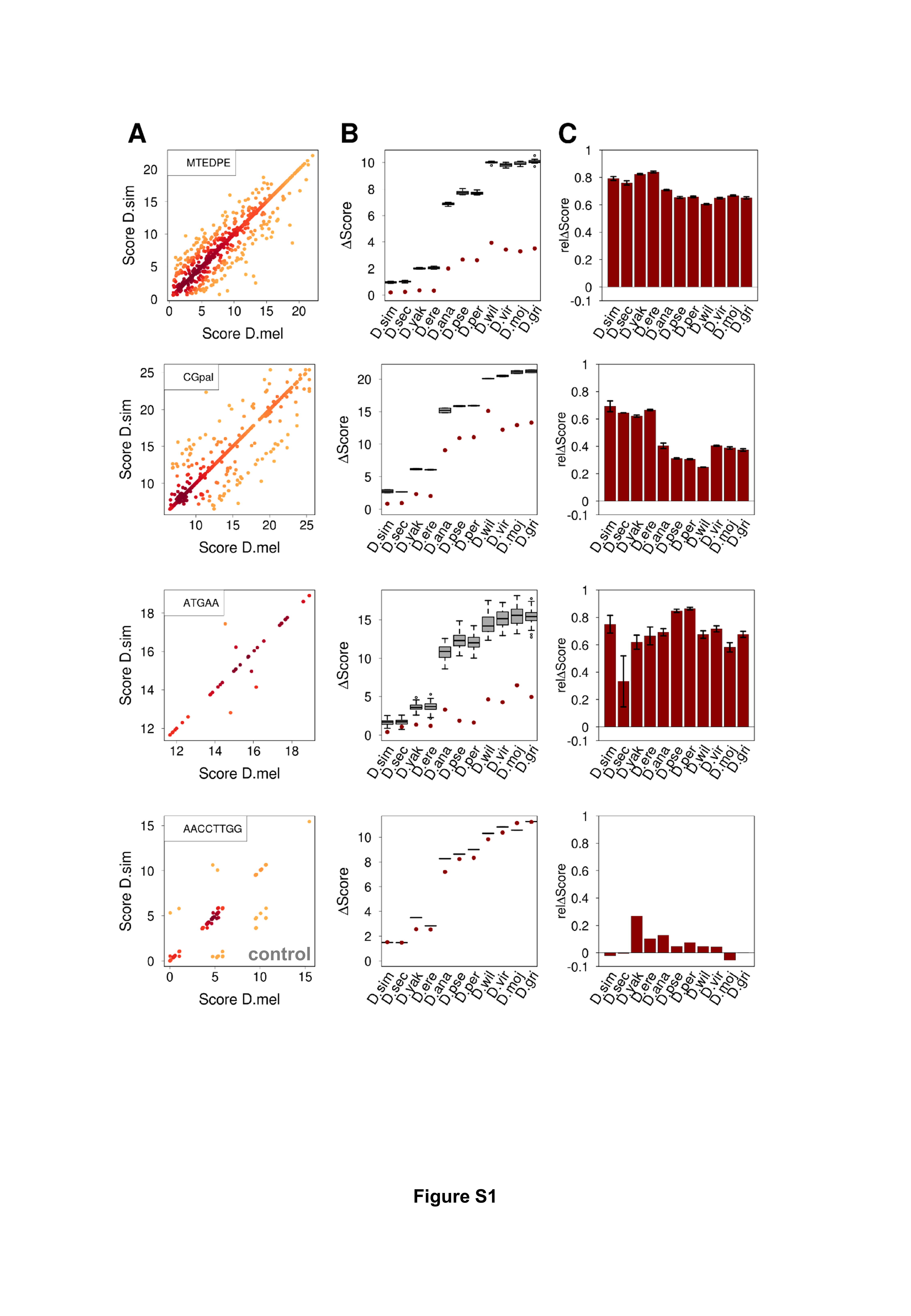


**Appendix Figure S1 (related to Fig EV2)**. Conservation of exemplary novel motifs (CGpal and ATGAA) compared to DPE and a negative control.

Conservation of two exemplary novel motifs (CGpal and ATGAA, middle panels) compared to MTEDPE (higher panel) and a negative control with a sequence chosen randomly (AATTCCGG, lower panel). (**A**) average sequence conservation of the motifs between *D. mel*. and related *Drosophila* species. (**B**) scores of the motif PWM for each site in *D. mel*. and the aligned site in *D. simulans.* The average PWM score difference between *D. mel.* and related species (ordered by evolutionary distance) for all assigned binding sites is shown in **B** as red circles. The boxes correspond to the expected score difference computed on aligned binding sites from sampled sequences. (**C**) depicts a scaled measure of the sampled and biological score distances, giving a one for perfect conservation of the PWM score and a zero for conservation as expected from background (conservation scores shown in **Fig EV2** for all CPEs). Error bars indicate the standard deviation over all sampled conservation scores.


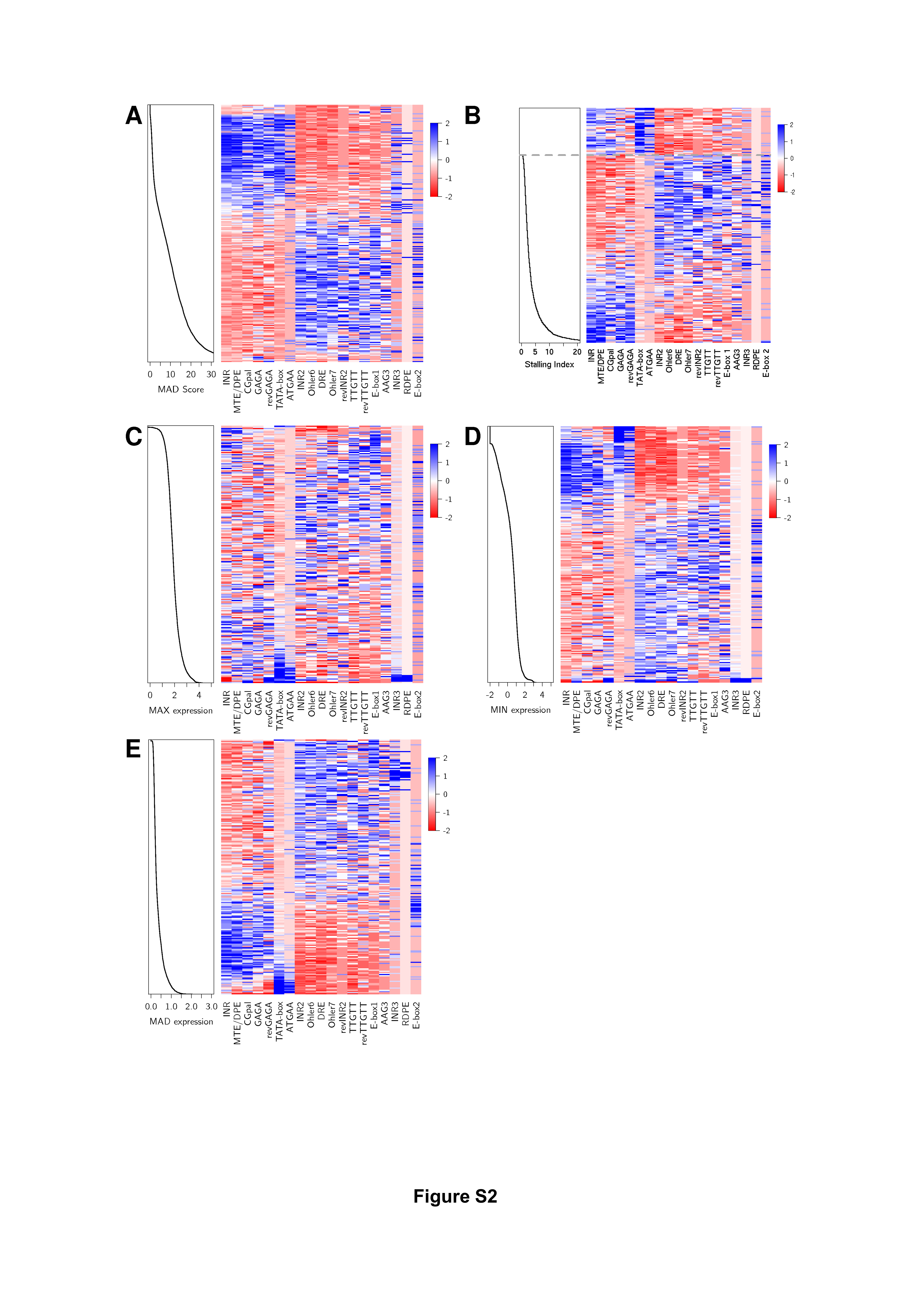


**Appendix Figure S2 (related to Fig EV3)**. Core promoter motifs correlate to distinct gene properties.

Genes are sorted by five exemplary scores (**A-E**, y-axis), while the Z-score – motif (x-axis) frequency within bins of 50 genes - is indicated from red (depleted) to blue (enriched). (**A**) TSS cluster width derived from TSS tag data, (**B**) Stalling index, (**C**) minimum expression, (**D**) maximum expression, (**E**) strength of regulation over 30 developmental time points.


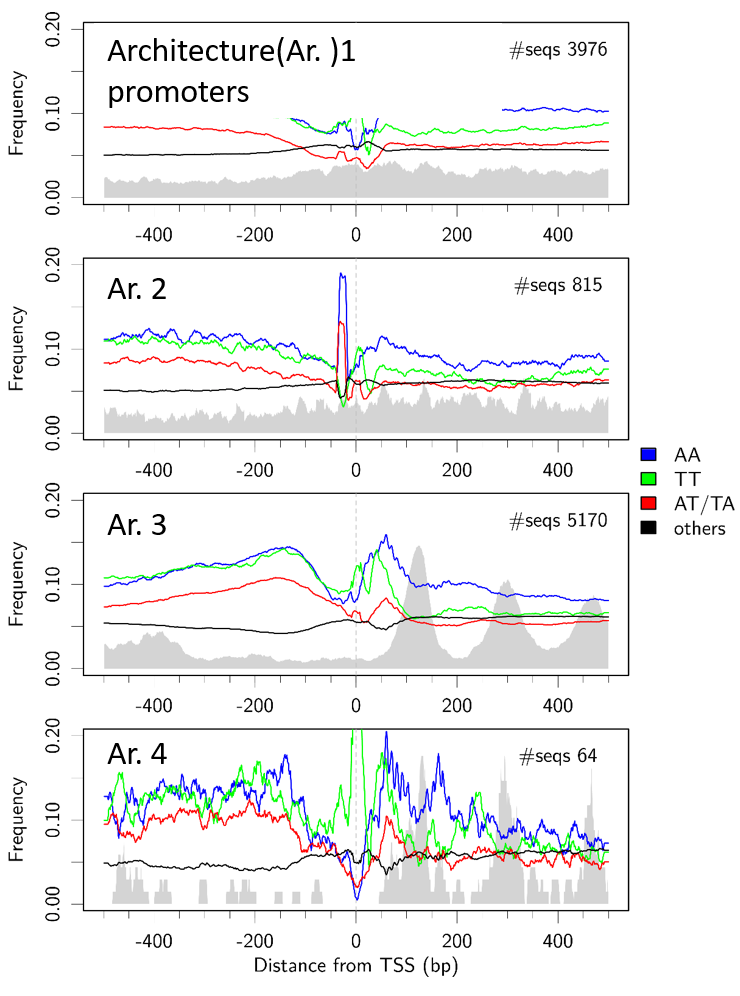


**Appendix Figure S3 (related to Fig EV3)**. The four promoter architectures have distinct dinucleotide/physical property patterns and nucleosome occupancies.

Each line corresponds to the average frequency of a dinucleotide over all promoters within the respective architecture, smoothed over 15 nucleotides. The gray background depicts the nucleosome occupancy (no scale shown). From top to bottom: Architecture 1 (Ar. 1) promoters (regulated genes), Ar. 2 promoters (highly regulated genes), Ar. 3 promoters (housekeeping genes), and Ar. 4 promoters (ribosomal genes). The number of promoters of the different architectures are indicated in the right upper corner.

All architectures show a strong composition bias for A and T containing dinucleotides, preferentially for ‘AA’ and ‘TT’, adjacent to the core promoter region located between -100 to +50 bps with respect to the TSS. However, the classes vary strongly in the shape of A/T enrichment and the most frequently occurring dinucleotides. Ar. 1 promoters show a strong ‘AA’ vs ‘TT’ bias within 500 bps down-stream upstream of the TSS that is reduced to around 150 bps for Ar. 2 promoters. Ar. 3 promoters possess two peaks of A/T enrichment, one ~200 bp upstream and the other ~75 bp downstream of the TSS. The downstream peak consists preferentially of ‘AA’ and ‘TT’ dinucleotides, whereas the ‘TT’ peak is located around 20 bps upstream of the ‘AA’ peak. In addition, Ar. 4 promoters show a second ‘AA’ peak located at around 150 bps downstream of the TSS.

**
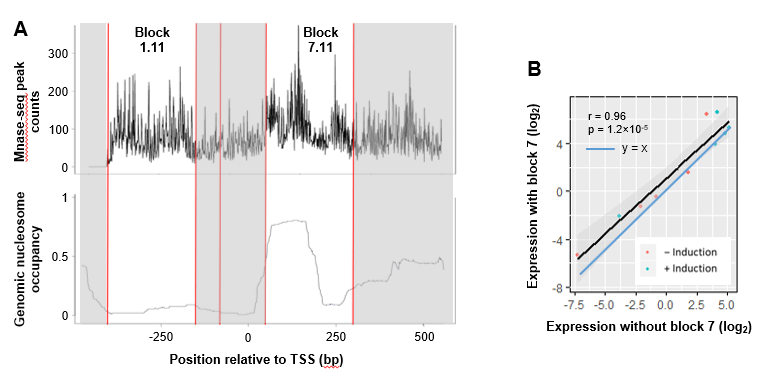
**

**Appendix Figure S4 (related to Fig 2)**. The effect of nucleosomal context on expression.

(**A**) Upper panel: *MNase-seq* measurement of the nucleosome occupancy for one synthetic promoter construct (*block 1.11* + *block 2* + *MED4 (native)* + *block 7.11*). The peak in *block 7.11* (highlighted in white on the right) suggests the presence of a +1 nucleosome with high occupancy; lower panel: nucleosome occupancy for sequences at similar genomic locations of the gene CG8613, from which *block 1.11* and *block 7.11* sequences were derived. (**B**) Comparison of the expression measurements for promoter variants with *block 7.11* versus without *block 7*. Expressions with and without ecdysone induction are labeled cyan and red, respectively. Black line: linear regression (with 95% confidence interval shown in gray, PCC r = 0.96, p = 1.2×10^-5^). Blue line: y = x.

**
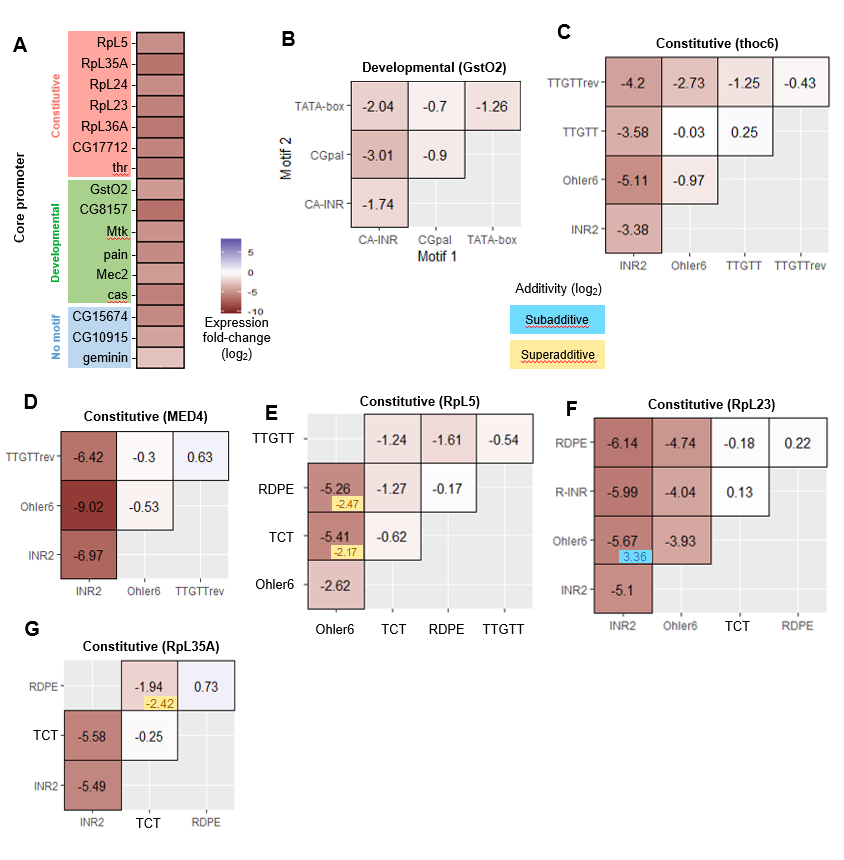
**

**Appendix Figure S5 (related to Fig 3)**. All-motifs and pairwise knockouts, consensus replacement, all-motifs shifts, and motif context exchange.

(**A**) Heatmap depicting the mean expression levels of promoter constructs with all-motif knockouts (log_2_ scale) for constitutive (in red), developmental (in green), and motif-less (in blue) promoters. The expression levels dramatically decrease for all investigated promoters. (**B-G**) The effect of pairwise motif knockout in different core promoters (log_2_ scale). Heatmaps of the mean expression fold changes compared to wild-type expressions for pairwise knockout of motifs compared to individual knockouts (diagonals) in GstO2 (**B**), thoc6 (**C**), MED4 (**D**), RpL5 (**E**), RpL23 (**F**), and RpL35A (**G**), respectively. Additivity was calculated as the difference between the pairwise effect and the sum of two individual effects. Subadditive (blue): > 0; superadditive (yellow): < 0; effects > 3×SD shown in the corner of each pairwise effect.

**
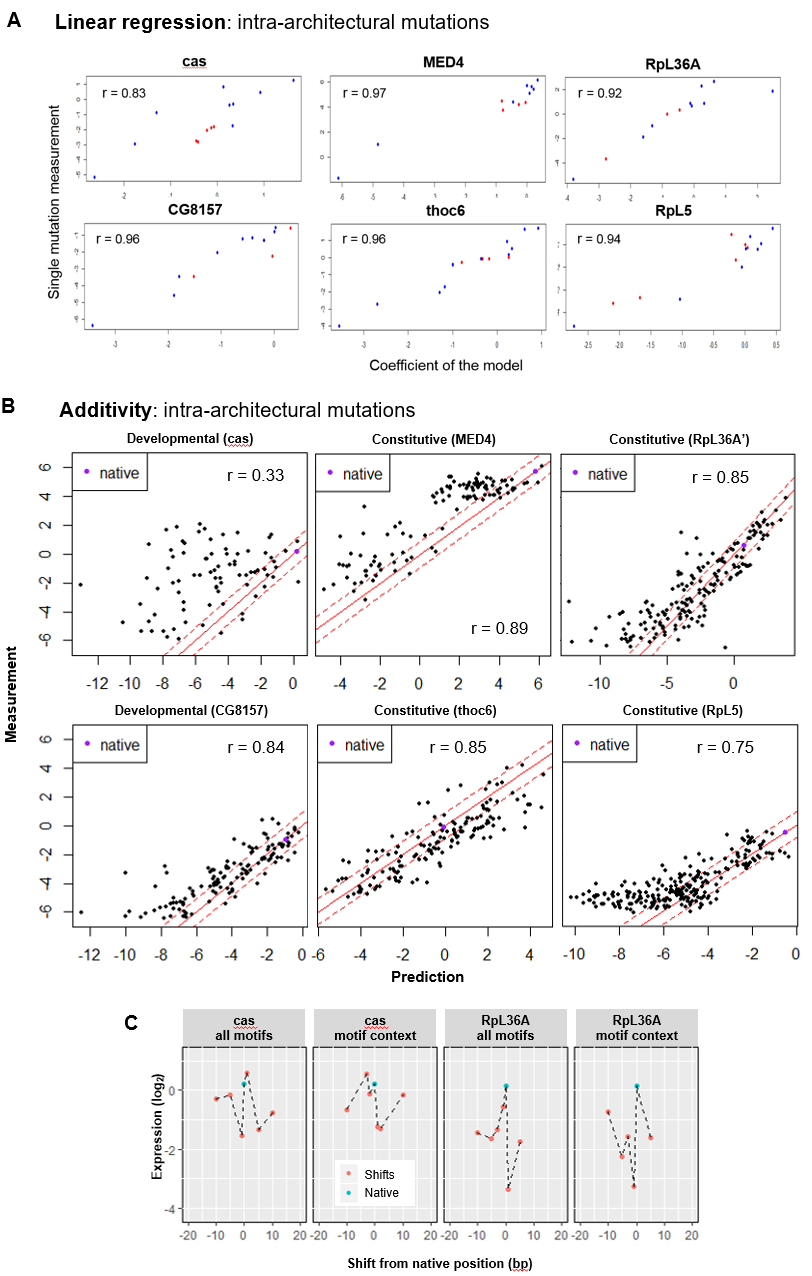
Appendix Figure S6 (related to Fig 6)**. Linear and additive models applied to predict the synthetic promoter activity based on individual motif features, and effects of all-motif shifts and context sequence shifts.

(**A**) The measured expressions of single mutations (on the y-axis) compared to the coefficients of the linear regression models for the intra-architectural combinatorial mutations (on the x-axis) in 6 tested core promoter sequences (log_2_ scale). Single mutations in motif strength and position are labeled blue and red, respectively. The average correlation PCC r = 0.93. (**B**) Additive models applied to the same intra-architectural mutations for the 6 tested core promoters to predict synthetic promoter activity based on individual measured motif features. The measured expressions (on the y-axis) of the sequences with combinatorial motif mutations compared to the predicted expressions (on the x-axis) from the additive model (log_2_ scale), which consists of adding directly the measured expressions of the individual features. Red solid line: y = x; red dashed lines: y = x ± 3×SD_noise_. Purple dot: the native expression of each promoter. (**C**) Effect of all-motif shifts and context sequence shifts. The expression measurements of natives (cyan dots) and positional shifts (red dots) of either all motifs or motif context sequence in cas and RpL36A (the two core promoter configurations also used for testing the individual motif shifts).

| **Motif Name** | **Start Position** | **End Position** | **Max Score** | **Threshold Score** |
| --- | --- | --- | --- | --- |
| CA-INR | -3 | -3 | 16.28 | 6.5 |
| CGpal | -100 | -20 | 26.46 | 3.7 |
| DRE | -100 | -7 | 15.78 | 7.3 |
| E-Box1 | -59 | 32 | 16.66 | 11.6 |
| GAGA | -100 | -33 | 31.27 | 0 |
| GAGArev | -100 | 1 | 29.40 | 2 |
| INR | -2 | -1 | 11.15 | 4.6 |
| INR2 | -60 | 20 | 22.42 | 8.1 |
| MTEDPE | 16 | 18 | 24.03 | 0.6 |
| Ohler6 | -100 | -10 | 15.94 | 7.5 |
| Ohler7 | -72 | 14 | 19.30 | 7.3 |
| RDPE | 8 | 12 | 28.38 | 14 |
| R-INR | -5 | -5 | 16.77 | 5 |
| TATA-Box | -35 | -29 | 14.73 | 5.5 |
| TTGTT | -32 | 40 | 15.94 | 1.6 |
| TTGTTrev | -14 | 38 | 18.56 | 2.2 |

**Appendix Table S1.** Summary of *XXmotif*-annotated core promoter motif features used in this work**.**

Motif 1^st^ nucleotide locates within the range between “*Start Position*” and “*End Position*” relative to the defined TSS. “*Max Score*” is the PWM score of the motif consensus. “*Threshold Score*” is the minimal score that maximizes the mutual information between a certain motif and all positively correlated gene sets.

| **Native Promoter** | **Sequence (5’-3’)** | **TSS Distribution** |
| --- | --- | --- |
| FBgn0003701  thr | TGTAGTATTTGTGTTCTGATATGAAAACTAGAGATATCGATGTTATCGTTAAAGGATTTCCAGCTTTAGCATGGACGGTCACACTGGATCTCAAAATCTGGCGCAAAGCAACAAAAAAGGAAGCGTCGCAG | BP |
| FBgn0004878  cas | CGGTGGCGAGAGGGTTGCACTTGGGCATGGATTTGCGCAATTTTTGCTATATTAGCCGGAGGCCGCGGAAGAGTTGAAGCAGTTTGAGCCTCGCAGCCGAACTTTGAGGATCGCTGAGACGAGACGCCGTG | NP |
| FBgn0010078  RpL23 | CTTGGTTATAATTAGGTTATTTTTTCGATATTTTGAGGTATATTTCTACGATAGATCGGCGGTCACATCGTATTTCCCTCCTTTTCGTTTTCGTTTCCGGCGAAGTAAGTATAATAAAATCTCCACGTTTT | NP |
| FBgn0014865  Mtk | CTCAAATAAAAAGTCCCCAATCTGCGACTCGTTTGTCTGGGACTGAGCTATAAAAGCCTCACCATCTCAACGCTCAAAGCATCAATCAATTCCCGCCACCGAGCTAAGtaGCAACTTAATCTTGGAGCGAT | NP |
| FBgn0027597  CG17712 | TTTAAATAGATTTAGCTAGAAAATAGCTGACAGACACATATCGATATATCGCTGCGATAGCCACAGCTGTTCACGCCCGCAGTTTAAGCGtaGTGGCAGCCCTGGTCGGCCACCAAAAAATAAACATTGGA | BP |
| FBgn0030993  Mec2 | GAGAGAACCAGTGCGCTCTTATCACGTGAGAACGCTTTTGGGCATTCAGTTTGGCTTTTGCGGCGCTGACCGCTGGCGACAGTTTCGAATCCATAGCCGATCGGAGAGCAACGAACGTAGGCCAGAACGGA | NP |
| FBgn0031980  RpL36A | CATATCAAGTCACAACAATGAAACGAAAACCTATCGATAGCGCATGGCTTGACGGCACGCTGCCATCGCTATGTGATTTCCTTCTTTTTTCGCCTTCACGAAATCAAGtaGGTAAGCGTTTCCCGAAATCG | BP |
| FBgn0032518  RpL24 | GTATTTTTTAGGTTTTTTCGTCTGCCCGTGGCAGCCACACTAATTTGGCTCAGCTTTTCGTTTCCACTTCCGTTTTCTTTTCTTTTCGTGTTTCTACGCCAGCAAGtaGAAGTACGTTAtaGAAAAGCGTT | NP |
| FBgn0033081  geminin | GACACGAAATCGAGGGATGAAATTGCATGTGATGCAGCCCCTTGAGCAAACAGTGTTGGACAACAGCGCGCGGCATCGGCATTTTTTGGCGGCCATTACAACGAtaGGAAAACTGTGAAGCGTTGCGCACA | BP |
| FBgn0034010  CG8157 | AGGTATCTGAAAGTCGAGACATAGTTAAGTCCACGCTTACAGATCGGGTATATAAAGAGGCCACTTTCAGAGCGGATTTCAGTTTAATAATTTAAAGCAAAtaGAAACTACTGGTAAGCTAGCTGGGTTAT | NP |
| FBgn0034308  CG10915 | CGCCATGCGCAGCACTATCCTGCGACTAAGCCAGATCTGACGGGAATAAAGCTGAATCGGCAGCACTGCCGCAAGTATCCACTTTTTCACGGGCAACAAATTGACAAAGAAATTGTAAGATAATTTCCTGT | BP |
| FBgn0034642  CG15674 | TGGTGCCGATTATCTTATCGCCAAGTGTGGACTGCAAGTTGGGAAAACGAATACATTCATCACCCCGGTCGTTGCTCACTAACTGGGTTTTCGGTGACGCTATTACGGACACGGACCGGCTCTCACCGAAA | NP |
| FBgn0035754  MED4 | AGTCTGGCAACCTCTCTGTTACGGTATTTTTACAACGTGGTATTAACAGCGCTCCGGAATACTATACGGTATATTTCAGCAATCGAAGAACGGCCACATTGCGGTGTGGAAAATAAACAAATTGCAATTAT | BP |
| FBgn0035906  GstO2 | ACAATCGAAATATATTCGATAAATTCACTCGTCCGGCGACTGCGACCACTTTATAAGGTACCGGAATCCCTCTATTTGTTGTCAGTCGATCGGAACTACTTTGCCACCAACATTTCACGTCTTGGGAATTA | NP |
| FBgn0036263  thoc6 | CTACGTAATATACTAACGCACTTTTAGGTATATTTTTCAAAAATAATATACTGTTCTTGGTATATTGCTCAGGAACGGTCACTCTAGAGAGCCGGCGTAAACAAAGCGATACAATTTGGTTAAATTAATTA | BP |
| FBgn0037328  RpL35A | ACACTTTCGAGCAACGGCGCGCTGTTTCACTTAACATATCGCGTTTTTGTGGCGTCTAGAGGCAGCCACACTATTTCCTTCTTTTCGCTTTCGTTTCCGGCGAAGTAAGTAAATTAAATTTCTCTGTATAT | NP |
| FBgn0060296  pain | GTGTGGCCCCTGTTAGCTTTCTGTTAAATTTAAATTTCTGTAAAGTGCCCGCCACTGCGGTCGCTTTCACGGATCAGATTAGTCGTTGTCTGGATATTAACGAGGAAGGTAGTGATCGCGCATTAGTGTCA | NP |
| FBgn0064225  RpL5 | TTGGCATTTATTATTTTTATTGTAAGGTATTTTTTAGTACATTTGTTTCTTGGATCCAATAAGGCCGCACTATTTTCCTTCTTTTTGCTAGCAATTTCCGGCGAGGTtaGTGTAAAATATTTCTACTCCAC | NP |
| FBgn0086519  Cpr47Eg | GACTTGAACTTGGGCGCCACCGGCAGAGGTAAGCTGAGCATCAGTATCATATAAAAAGCAGGCAGAAGTTCCAGTTCGATATCAGTTAGCCTTCTCAAGTCTTTCAACAATCAACtaGAAGTTCTTCGTAA | NP |

**Appendix Table S2.** The sequences for the 19 native core promoters investigated (with TSS downstream ATGs mutated). The TSS distributions for these promoters are indicated as narrow peak (NP) or broad peak (BP).

| **Mutation type** | **Number of synthetic promoters designed** |
| --- | --- |
| knockout of motifs (individual or pairwise knockout of motifs, and knockout of all motifs) | 260 |
| replacing the original motif with sequences with different PWM scores or insertion of the consensus into the motif-less promoter sequences | 170 |
| point mutation of motifs | 596 |
| substitution with functionally or positionally equivalent motifs from other architectures | 78 |
| shift of motif positions | 164 |
| intra-architectural mutations | 2023 |
| inter-architectural mutations | 478 |
| context swap | 30 |
| native sequences | 19 |
| native sequences with ATGs | 8 |
| **TOTAL:** | **3826** |

**Appendix Table S3**. Number of synthetic promoters designed for the different mutation types. Among the total number of synthetic promoters designed, ~3000 could be recovered and tested during the experimental procedure (see **Material and Methods**).

| **Block 1** | **Sequence (5’-3’)** |
| --- | --- |
| 1.1  **+** | CGCAGTGCAGTGAATCATCCGTGGTGACCCATGGCTCTCGACTTACAGAGCGGCTCTTGGTGTTTCCCCGGTCGTAGATACACTACACTGAACGAAAATTTACGAGCCGATGCATTTACATTCCATTCCATTACATTCTCTTATATGGCATGTGCTCAATTGCTGTGGAGGATGTACGGACTAGAGATCGCCTCTTTCAGTGGCGGCACACTTGGTTCGTGGTGTTTCAGTGCAGTGCT |
| 1.2  **−** | TTTCCAGTGTAGAAACGCGTTTATAGATGTTAAATATTAACCACTGATAAGTACAAGCTAATAACAACAATAATCGTAACAACGCGCTCTGGTTTTCTCCGTGTGCACTCAAGAGCGTTGCTGATTGAAGCGGATGAAGCCGAAGCCGATCGGAGTTGGTCAATTTTCTAAACTCTCTCACGGTCTTCAATTGAACGGCACTTCCTCGACTTCCTCCCGTCGCCCCCGCCCTTTCACAC |
| 1.3  **−** | CGCTCCAAGCTAGACTCAAGAGAGATACGCACCGGAGATACGCAAACCGGTCGTTGGCTGGCTAGCCGTAGCCAAATATTATGCTAATTCGACATTTTTGACAAAAATAATTCGAGAATTAATTATATCTTTACAATGTGTCTGCTAGTTGACACATAGTTAGTTAATGTCTCCGCGTCAAACTCGTCTTCCGTCTGTTTCGGGCATTATTATGGGATTCAATGCGAACTTTAACTGAA |
| 1.4  **+** | TGTAAAAATTTTATTTTTGGAAAATCAAAAAACTAGTGATAGGGATAGTTAGGTATGTTTATTAGCAGTACAAAACAGTCTTTATTTTCGCTGTGCCACCTTTTTTGGCCAAGTTTTGGAGAAAACCCCGTACGGGCATATCAGATTTTTAGCATCATCTTTTGCAGCGCTGGAGGAAGCCAGAGTTTTCACTCACCAATAAAAAATGTAACTTAGTAACTTTAGCCAGATTTTCCGTT |
| 1.5  **−** | TGCTGCAAGTTGCTTGCTGGTTTGTATGTTTTAAGAGTGAAACTGACGGGAGACGCGAACGCGAGGATGCAACAGAGTATTGTAATCTGCCATATTTGAGAAGGTTTGTTAGGTTTGATTTGGCTAATCAACAGAAGAGTTTTATGCTAAAATTGTAACTGCAATTGTAACACACGAGAAATAAACAAGCAAATAATACCTACTAGAGATAACGCTGCGAATTTGTTTTATTTTTGAACT |
| 1.6  **−** | CAGTTGGCGCGATAGCAGGACTTATCAATTGAATAACAGGACCTTATTATCGACAAAACCTTAGAGCTGCCACGCAATTTAATAAAGTTATCGTTCGTAAAGTTATGTCGAATTTAGATTTAAATTGAAATTGATGAGGTAATATATTTTTAAAATAAAATCCTATCACTTATTGTTGCTTAAACTAAAATTTGTTTCAAGAAAGACTATTATGAGATAGATCTTCGACTAAAAATAAC |
| 1.7  **+** | TCCCCTCTGCACCATCGTAAATATACGACTTTTATTTATTACTTCATTTTATTTTCATTATTACTATCTTTGAATAAAGAAATTTCAGAATACCAAACAAAATGATTTTCGTTTCATTTTGAATTAAAATTTCACCAGTGGGGGAAAATAACGGTATTTGAACAATAATGGCGTTGCGAATGTAACGTGATTGGCGTAAAATCGCAAATTCCGTTTTATATAGTTGAATGATTTTCAAA |
| 1.8  **−** | GCTGAAATTCCGATTTTAGGCCGCTGTCCAGTTTACAATTAATGAGATTAATCGTAAATAAACATTTACGGAGAGCACTCTTACTTCTGACACACACGATAATTTGTGGCAGACACATGGAGAATGAAATTTTTTATGAGGAAATCGAATTGCAATCTCCGGCGAACGATTTTGCTCATGCAATTGCAATTACAATTGGTTTTCAATTGTATTTCCTCAAAATCAAAAGAATGTCGAAT |
| 1.9  **−** | ACCGCAGTTTCCAGCTGGCTTGAAATTTTGGCCGGCGACTCCTCCTCGTTGGCAGTTTTTAGAAAACGAATCGCCTGTGCAGAGGCCAAGGCTCCGGCCGTAAAAGAGGTCAGCAACTGAGCAATCGAAATCATTTGAAATTTGATATTTTTAAATGATTGGAAAGCGAATTTAGATCATGCTTTGAAAGTTCGTTACGATTGCTATGAATTGAGTTTAAATATTTCAAGGCTACATAA |
| 1.10  **−** | GTGGGCGTGGTGCCCGGCAAGGTGTTGTACTGCCAGTGCGGGGCCCCCAATTGCCGCCTTCGTCTGCTCTAAGTTCTAGCTTAAGTTAGAGATCCATACAGGAAATATACTCATTAAAGAAGAATTAGAATTAAAAATTTAAACTTTAAACATTATCTGTTCCGTTAGGGTAACGGAAACATTGCATTTTTATAAGCTACTGCTGTTCTGCACCGTCCGTTTCAAAGTACACAATTTTC |
| 1.11  **−** | TTGGGGGAACAGCCTGAAAGTAGGCTACAAAACTCTTGTCTTCAGTACTCTTTATCGTGATTCCCACGACGACTTTCTTGCTTTTACATCATAAACTCAATGTGGTAATAAATTTAAAAATAGTTATACTTTTCTGTCATATTCACCAAAAGCTGGAAATTTGTATTAATTTTAATGTTGATATGAGGTCAAACGCATAAAATAAATGTATAAAAGATGTTTTGCTTACTCCGAAATCA |
| 1.12  **+** | AATTCGGGCCTGCCCATTCAGACAGCCAGTGACTTGGATGATGCCGCCCACAAGGCTGTGGCAGCCCTTAATTAGGGGAACGATTGAGGAGAGCATGTCTTCCAGAATGAAACGACGCTCATTAGCATTTACAACGGTTGGGCCTTTTAAGTATAAGTTTTTATCGACAATATAACCAAAATATGTTATATTCTATATAAAAACCTTTTTATTTGATTTAAGAAGTACCTTAGCCATCT |

**Appendix Table S4.** The sequences of block 1s used in this work.

“+” in column “Block 1” represents well-positioned -1 nucleosome pattern found in the genome-wide *MNase* digestion of chromatin; “**−**” represents not well-positioned -1 nucleosome pattern.

| **Block 7** | **Sequence (5’-3’)** |
| --- | --- |
| 7.1  **−** | AGCATAGGAGCCGCACCAGGATTCGCCCGTGTACGCCAACTACGAAGATTAGCGCAACTCTGGGCCGGCCAGCTCCACGGCGTACTAGGTCAACTAGGGCGCCGCCGGCTAGGCACCCGAGCCGGCACTTCGAGTGCCCGGCACCACGCAGCAGTATCGTGGATTCAAAACTTGTAAGCGGGCGGAAACGGTGTATACTATAGTAGCAGCAAGTAGTAGACCCATCCCGGCCAATATTAG |
| 7.2  **−** | GTGAAGTGCATTGAAAGCAGAGCGAATCAGAACGAAATTCAAAACGTATCACGCATACGCCCGGTAGTACAGCCGAATATCCCCAAATAGCCGAATTAGTCGCCGAGAAGGAGCTTGCTGTGCTGCCTGCTGGTGAGCAGCATCCTAGTGCTGCACCAGGCTCAGGCCAACATCGAGACAAACGTAGTGATAGACCCCAGTTACTACAGTAAGTGTGCGTAGTGGATCAGGCGGCCATAG |
| 7.3  **+** | CGCAAATTGATCCTTTATAGATTCTAGCTGATTTGTTATTTACAATCCTTGTAGATTGCTTTCGCCTGCCTGTTGGCCGTCGCCCTCGCCAACGAAGTAGCCATAGTTCTCCGTGCCGAGCAGCAAGTGATAGTTGACGGCTTTGCTTACGCTGTTGAGCTGGACAACTCTGTCATAGTGCAACAGAAGGGTGACCTTAACGGCGAAGAGTGGGTGGTGAAGGGAAGCCAGTCGTGGACA |
| 7.4  **+** | GGATTGATAGGCGTTATCAGTCGAAATTGAAAGGGTATAGGACCAGGGCAACTGCCTGTAGCCCGACATCAATATCTGCCAAAGCGACTTGGCCAATCCCACCGAGCCCATTGTCACCAAGATCTAGGTGCACTATCTGCGGAGTTTCGGCTTTCGCCTGGAGCCGCCCTATAAGATTGGCACCGAACTCGGTCACTCGTCGCGGGAGGCGCGCGTCTTTCTTATCCGAGTGTGCCGCCA |
| 7.5  **+** | AAACGATTTGCCGTGATATAGTCGTTTCATTTGAGCACAAAAGAGCGCCTTCTGCTCCTGATCGACGACATTGAGTAGATTGCCAAGGAGTTGATCGAGCAGGCGCACCAGAAGATCTCCAGCACCGAATTGGTGGACCTGTTGGATTTGCTGGTGGCCAAAGTAGAGGAATTTCGCAAATAGCTGGAACTGGCGGAGGAGCAGGCGAAAGTGGAGGAGGCGTAGGACCAACTGCGCGCT |
| 7.6  **+** | AAAAAATAATAAAATAGTTCACTTAAAATCCATAGCCACCTAGAATTTAGCTTGCGGGAAGCCTGCATAGGAGTTTTGCCTCCGCCGCCAAGGCCGCAAAAGCAAAGCCCGTCAAAACCAGCACCATTTCAGCTGTCGGCGAGTCCATCGCCGCCAAGGGGTAAGCACTTTCTTTAATTTATTTACATAAAAACCAAGTAAATATTCTCCTGATTGTAGCTTCTTGCGTCCCCATAAGCC |
| 7.7  **+** | CTAAAACACCGACTAAGGACCTCTGAATAGGACTCTACTGCCACTCGCACGCTGCCCTTTGTGTACAAGTATAAAAATCATAAAGGCCAAGACTGCGAATCCCGGCTGGACATCACTTTTCCCTTTGACCAGGAAAATCTGGAGGAGTGTATCACCCAGCTGTAGGCTCCCAACCAGTAGGACCCTTAGTAGCGGTACCTGGACGAGAATATCAGTGAGTATCAGTTAGTGCGGTAGGAG |
| 7.8  **+** | AAATTAGAAACAAAAGGATCTGAAACGCGCTTACAACATAGTCCTAGCACAGGCTATATCCGGCTCTAAACAGTACCTATTTGCGGGAAATCTCTTTGGCGACATTTTCGTACTTAGGTGAGAATCCTTAATTTGGCGCAATTTCCCAACTAATTGTTTTTCTATTTTGAAGAATAAAAGAACTGGACAAGGGTTCCGAGGAGCCACCTGGCAAACTGAAGATCTTCCCGCAGGGCAGCG |
| 7.9  **+** | AACTTTATAGAGTTTTATCCAATTTGAGCGTAGCTGCCGGGCGTTGGAGTGTTCGGAACGGGAGAGATAGCCATAGTGCTGGTGCCGCTGCTGCGGGAGAAAGGATTCGAGGTGCGGGCCATTTGGGGCAGGACCCTGAAAGAGGCGAAAGAGACTGCGACCACGCAGATAGTACAATTCCATACGAACGTAATCGACGTAGTCCTGCTGCGGAAGGTAGTGGATCTGGTGTTCATCGTG |
| 7.10  **+** | ATTCGAGAGTAGCTAAATTAGGGGCCGCATAGCGCCAAGCTCACCTGTAAGTGGCTCCAGCAAGTGTCTCGTCGCAGGTGCACAGGTGCTAGGCAGCAGGACTCCGCCGACGGCTGACTAAGAGCAGCTTATCCGCCCCTTGGGAAGAAAAAATCGATTTTGTGATAACGGGCTTACGCTTACGCGTTTTTGCACTGCTAGTCGGAACCAATTGCCAGTAGACTCCACACCTAGATAGTG |
| 7.11  **+** | CGATTGCACACGTTGCACTTTTGTTGGCATTGGACGAGGTCGAGTGAAAGCACAGCGCAAGCCCCGAGAATCCCGATTCGTTTGCTTAGTTCAGGGTCAGGCCGCTCCTCCCCAGGATACGAAGCTCCCTGCAAACGACCTTGAAACTCCAAGCAGATACATCGCAATCCGAATCCGAATCGTTCCGTCGGAACCTACGACTCCTACACAACAGTTGTCGTAGTCGCCCTGTGAGCAAGT |
| 7.12  **+** | TAGAATTTCCCACTCAAAGGTAGCAAAAACCATCCCACTTCGCACGCGAGGATTCCCAGGAGCACCTGCCCACCAGCCACACGCAAAACAGTCACACAGTGAACTAGAAGAGACGCACACTATCCGGAAGTTGTGGCGTTGGCGTCTTCGTGTTCGCCTTTGCCTTCATCGTGATTGCGTTTGCAACGCCCAGTTGGTTGGTCAGTGATTACCGCATCACGGGCGCCAAGCTGGATCGCC |

**Appendix Table S5.** The sequences of block 7s used in this work**.**

“+” in column “Block 7” represents well-positioned +1 nucleosome pattern found in the genome-wide *MNase* digestion of chromatin. “**−**” represents not well-positioned +1 nucleosome pattern.

| **Block** | **Coefficient** | **Standard Error** | **t-statistic** | ***p*-value** | **Significance** |
| --- | --- | --- | --- | --- | --- |
| (Intercept) | -2.65885 | 1.36776 | -1.944 | 0.053117 | . |
| Block3_ RpL36A | -1.57372 | 0.61621 | -2.554 | 0.011297 | * |
| Block3_ CG8157 | -2.73639 | 0.62579 | -4.373 | 1.86×10^-5^ | *** |
| Block3_ RpL5 | 0.15807 | 0.61497 | 0.257 | 0.797373 |  |
| Block3_ Cpr47Eg | -2.20657 | 0.61994 | -3.559 | 0.000451 | *** |
| Block4_ cas | 0.09943 | 0.26785 | 0.371 | 0.710835 |  |
| Block4_ RpL36A | 1.75593 | 0.27639 | 6.353 | 1.11×10^-9^ | *** |
| Block4_ CG8157 | 1.88443 | 0.27366 | 6.886 | 5.37×10-^11^ | *** |
| Block4_ thoc6 | 0.19417 | 0.26369 | 0.736 | 0.462259 |  |
| Block4_ RpL5 | NA | NA | NA | NA |  |
| Block5_ RpL36A | 0.35498 | 1.4742 | 0.241 | 0.809927 |  |
| Block5_ CG8157 | 0.38067 | 1.47562 | 0.258 | 0.796657 |  |
| Block5_ thoc6 | 2.07272 | 1.47939 | 1.401 | 0.162536 |  |
| Block5_ RpL5 | -0.35306 | 1.48039 | -0.238 | 0.81171 |  |
| Block6_ cas | 1.97382 | 0.37046 | 5.328 | 2.35×10^-7^ | *** |
| Block6_ Mtk | NA | NA | NA | NA |  |
| Block6_ RpL36A | -0.05506 | 0.25839 | -0.213 | 0.831442 |  |
| Block6_ CG8157 | -0.70079 | 0.26107 | -2.684 | 0.007793 | ** |
| Block6_ thoc6 | -0.07401 | 0.29249 | -0.253 | 0.800463 |  |
| Block6_ RpL5 | -0.14806 | 0.26584 | -0.557 | 0.578106 |  |

**Appendix Table S6.** Coefficients of the linear regression model for the inter-architectural block-wise combinatorial mutations.

Significance level codes: p ≤ 0.1; *p ≤ 0.05; **p ≤ 0.01; ***p ≤ 0.001. NA: not defined because of singularities.
