# Supplementary material for: Large‐scale analysis of Drosophila core promoter function using synthetic promoters
Source: Mol Syst Biol. 2022 Feb 14;18(2):e9816. doi: 10.15252/msb.20209816 (PMC8842121; doi:10.15252/msb.20209816)
Supplement: Supplementary file 2 — Expanded View Figures PDF [file MSB-18-e9816-s001.pdf]

Expanded View Figures

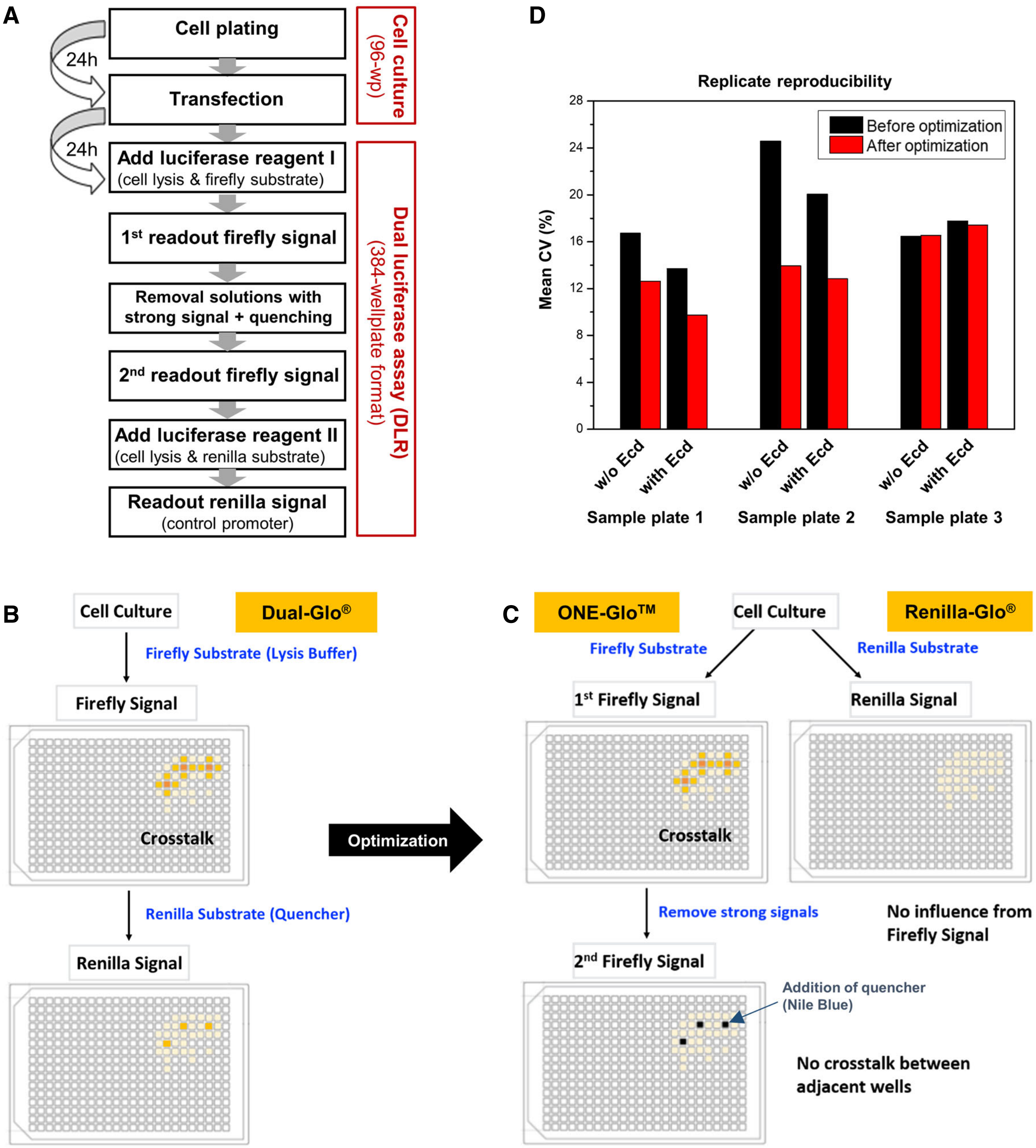

Figure EV1. Assay development and reproducibility.

A Workflow for automated transfection in 96-well plate format, followed by dual luciferase (DLR) assay in 384-well plate format (Materials and Methods). The transfection, lysis, and cell detachment occur in four cell culture 96-wellplates, followed by their splitting into two 384-well plates for separate readout of the Firefly and Renilla luminescence signals. This enabled to gain 4-fold higher throughput and to save 2/3 of the luciferase assay reagent.

B, C Experimental strategy to eliminate *crosstalk* artifacts. Separating Firefly and Renilla readouts (the two upper panels) avoids potential *crosstalk* between the Firefly and Renilla luminescence light within the same well. A second readout of the Firefly signal after removal of the solution from wells with very strong signal eliminates the *crosstalk* between neighboring wells.

D Comparison of expression level measurements for three 96-well plates containing the same promoter construct samples measured on different days with and without ecdysone induction. Standard normalization (before optimization) uses only the ratios of Firefly and Renilla signals. After optimization of data normalization procedure of the luciferase assay readout (Materials and Methods), the mean reproducibility of the measurements improved from a mean coefficient of variation ~13% after optimizations, versus ~18% before.

© 2022 The Authors

Figure EV2. Core promoter motifs detected by *XXmotif* and subsequent analysis to validate the motifs.

A Core promoter motifs of *D. melanogaster* we detected using *XXmotif*. Motifs underlined in blue are the ones used in our experimental pipeline. The first twelve motifs were previously described in literature, while the seven below the gap are novel. Column "*E-values*" indicates the significance level obtained with *XXmotif*. Column "*Distr*" depicts a smoothed distribution (over five nucleotides) of all identified binding sites within the gene set having the highest mutual information (and positive correlation), indicated in column "*Gene set*" (details in B and in Appendix Table S2). *XXmotif* reports the region (relative to the defined TSS) with the highest enrichment of binding sites (Column "*Range*"). Column "*Conserv.*" indicates the average conservation of binding sites, where 1 represents a perfect conservation, and 0 the background conservation. The bars correspond to 11 related *Drosophila* species, ordered by ascending evolutionary distance (details in Appendix Fig S1). The novel motifs are highly conserved. Column "*Occ [%]*" gives the frequency of motif sites within the whole sequence set (the gene set of highest mutual information).

B Legend for the abbreviations used in the column "*Gene Set*" and in Fig EV3.

 © 2022 The Authors

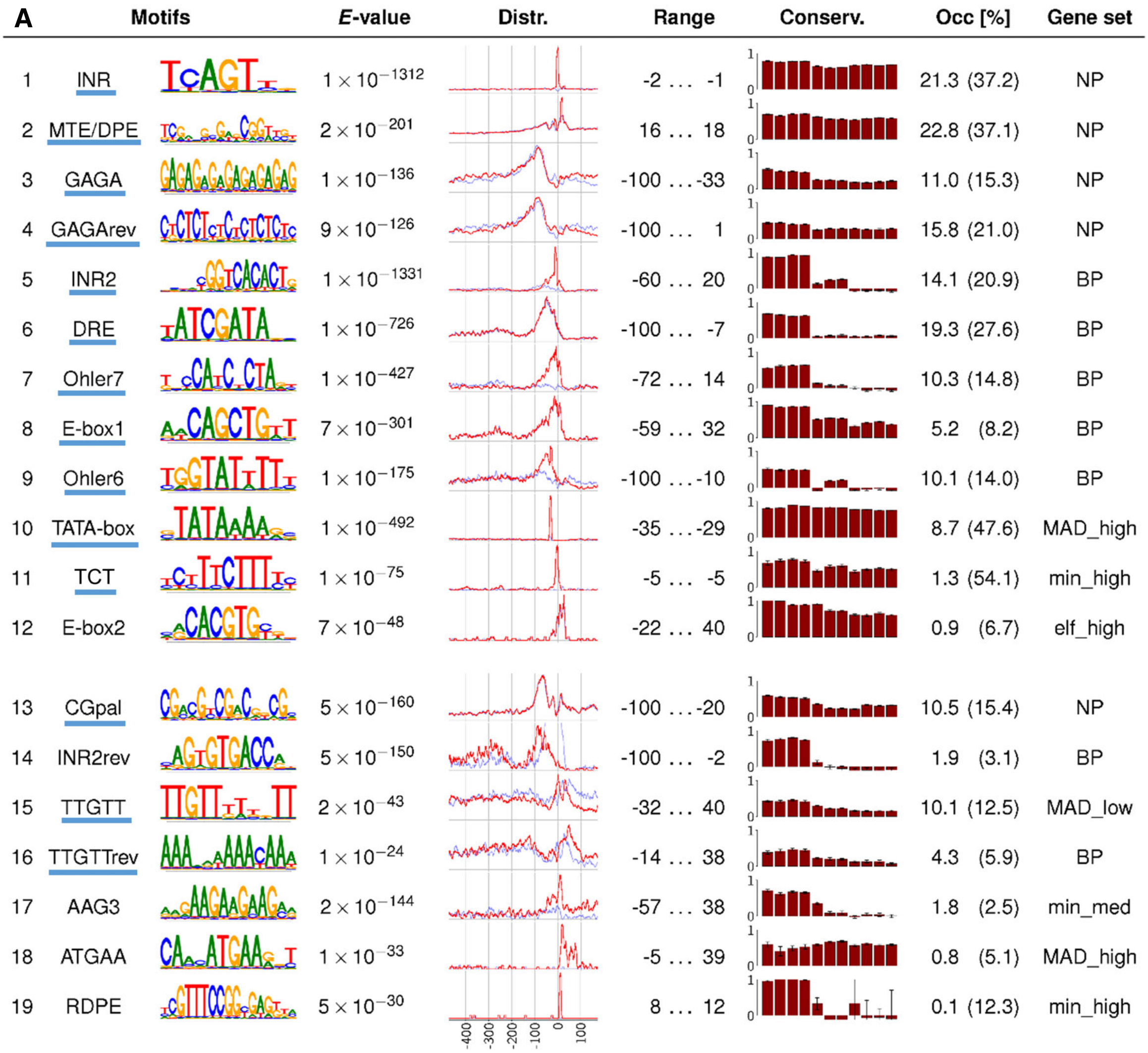

B

Gene Set	Definition
NP	TSS cluster width: narrow peak
BP	TSS cluster width: broad peak
MAD low	Mean absolute deviation (MAD) expression: low
MAD medhigh	MAD expression: medium to high (top 40% of genes)
MAD high	MAD expression: high (top 10% of genes)
min off	Minimum gene expression: nearly no expression
min low	Minimum gene expression: low
min med	Minimum gene expression: medium
min high	Minimum gene expression: high
max low	Maximum gene expression: low
max med	Maximum gene expression: medium
max high	Maximum gene expression: high
elf low	Gene expression in embryo, larva or female: low
elf med	Gene expression in embryo, larva or female: medium
elf high	Gene expression in embryo, larva or female: high
adult low	Gene expression in adult: low
adult med	Gene expression in adult: medium
adult high	Gene expression in adult: high
stalledPol	All genes classified as stalled by (Hendrix et al., 2008)

Figure EV2.

© 2022 The Authors

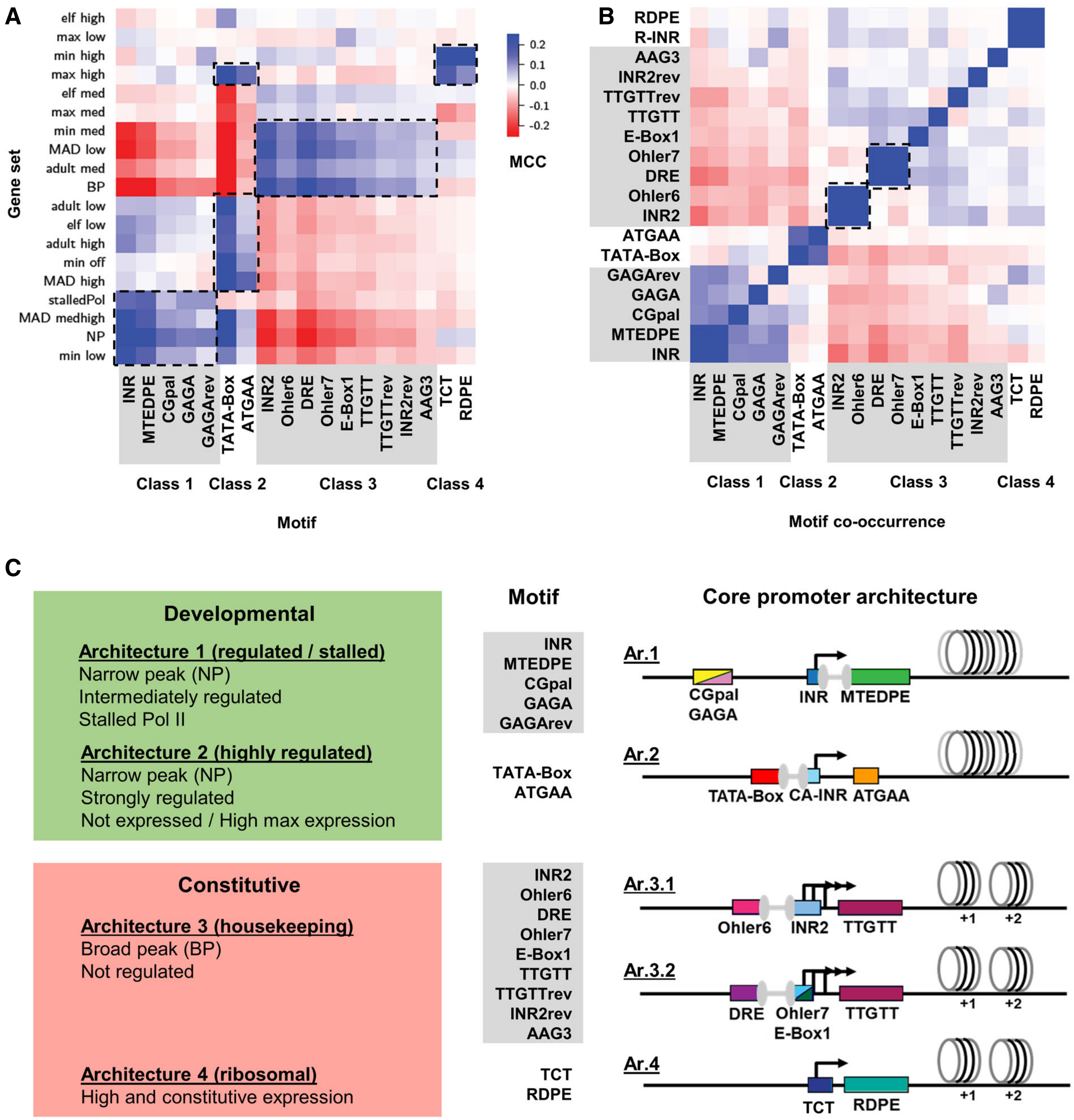

Figure EV3.

© 2022 The Authors

◀ **Figure EV3. Genome-wide analysis of promoter features.**

A Correlation of core promoter motifs with different features (motifs and meaning of the different features listed in Fig EV2B) reveals four distinct motif classes: class 1 motifs enriched in the gene sets of stalledPol, MAD medhigh, NP, and min low; class 2 motifs enriched in the gene sets of max high, adult low, elf low, adult high, min off and MAD high; class 3 motifs enriched in the gene sets of min med, MAD low, adult med and BP; class 4 motifs enriched in the gene sets of min high and max high (details on the motifs in Fig EV2A and on the different gene sets in Appendix Table S1). MCC: Matthews correlation coefficient. Groups of core promoter motifs that correlate strongly positively with particular features are highlighted with black dashed boxes.

B Core promoter elements co-occur in architectures. Correlation of all core promoter elements to each other reveals elements that occur preferentially within the same promoter (positive correlation, blue, examples highlighted with black dashed boxes) or avoid each other (red). With the exception of the housekeeping class (class3)—which consists of two architectures (see C)—each promoter classes matches one architecture. In agreement with the four identified classes, most CPEs are positively correlated to all elements within their class and negatively correlated to CPEs belonging to other classes. Only the Class 4 elements are positively correlated to some motifs of especially Class 3. Negative correlations between elements within the same class are only found for elements located on both strands (e.g., GAGA versus revGAGA, TTGTT versus revTTGTT) and for two groups of elements within Class 3 (highlighted with black dotted squares): Class 3A (DRE and Ohler7) and Class 3B (INR2 and Ohler6). The two groups are correlated internally and anticorrelated with each other, indicating that the elements of each group bind a complex together. The remaining elements of Class 3, TTGTT, revTTGTT, revINR2, and E-box1 show weak positive correlations to all other elements of their class suggesting that both transcription initiating complexes acting in this class have overlapping subunits.

C The four core promoter architectures identified. Class 1 motifs (INR, MTEDPE, CGpal, GAGA, GAGArev) occur in genes with NP core promoters (Architecture 1, 3,976 genes). The enriched genes are intermediately regulated and show strong correlations to stalled Pol II. Class 2 motifs including TATA-Box and ATGAA also present in NP promoter genes; however, the enriched genes are strongly regulated ones that are either not expressed or most highly expressed in at least one developmental stage (Ar.2, 815 genes). Class 3 motifs (INR2, Ohler6, DRE, Ohler7, E-Box1, TTGTT, TTGTTrev, INR2rev, AAG3) are the ones only found in genes with BP core promoters (Ar.3, 5,170 genes). The enriched genes are not regulated and similarly expressed in all developmental stages (housekeeping function). Ar.3 can be further subdivided in two additional sub-architectures Ar.3.1 and Ar.3.2, as discussed in B. Class 4 motifs (TCT, RDPE) correlate with strongly expressed genes which mainly encode the ribosomal proteins (Ar.4, 64 genes).

© 2022 The Authors

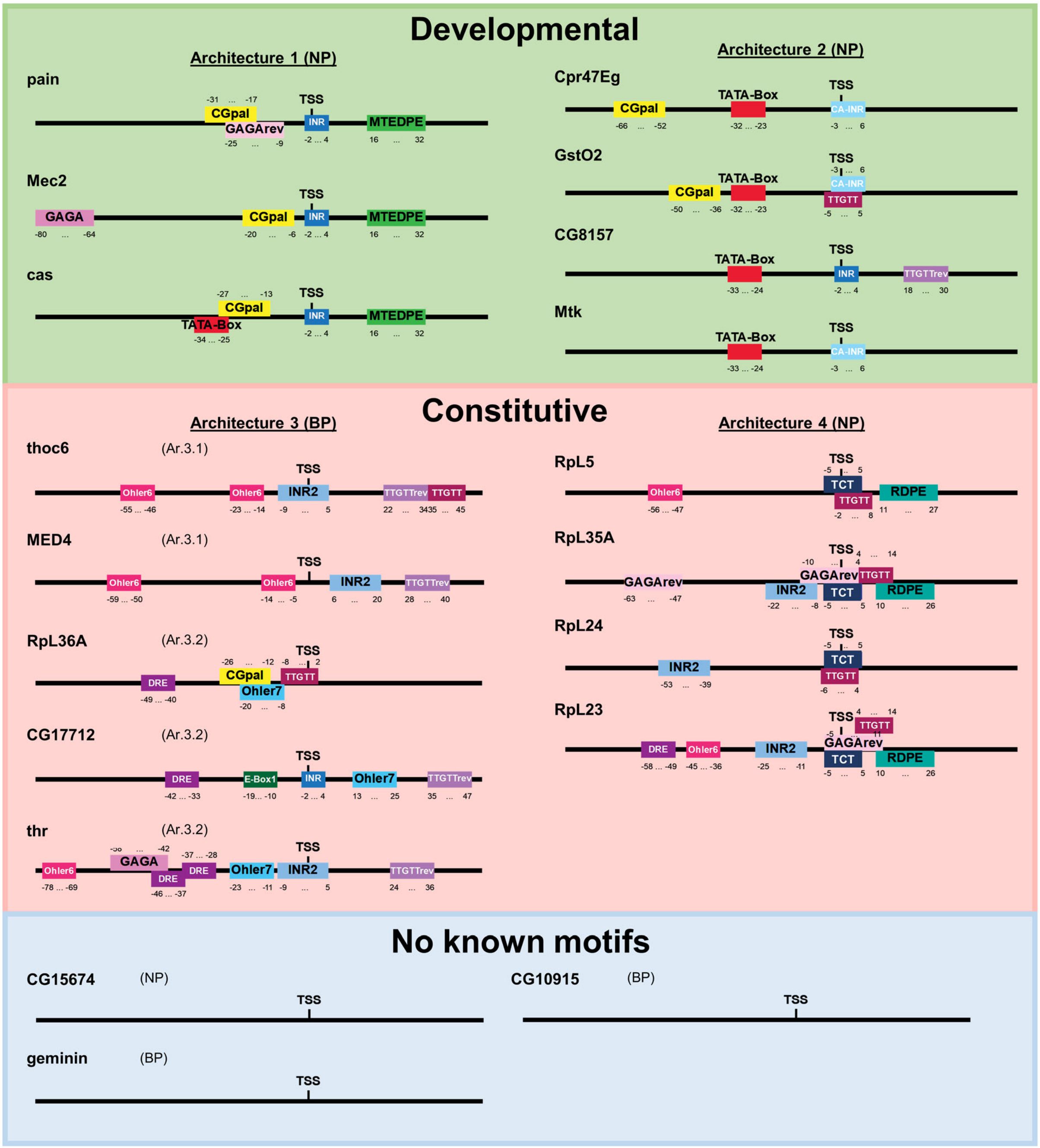

Figure EV4. The wild-type core promoters selected in this study and their motif composition.

Two-to-four native sequences were chosen (position −80 to +50 relative to TSS; TSS itself at position 0) from each of the four core promoter architectures Ar.1, Ar.2, Ar.3 (Ar.3.1, Ar.3.2), Ar.4 defined in Fig EV3, and one additional architecture with no known motif (termed motif-less promoters). In total, 19 wild-type core promoters with annotated motif positions are shown here. NP, narrow peak; BP, broad peak. Their sequences are listed in Appendix Table S2. Developmental and constitutive promoters are highlighted in green and red, respectively. Motif-less promoters in blue.

 © 2022 The Authors

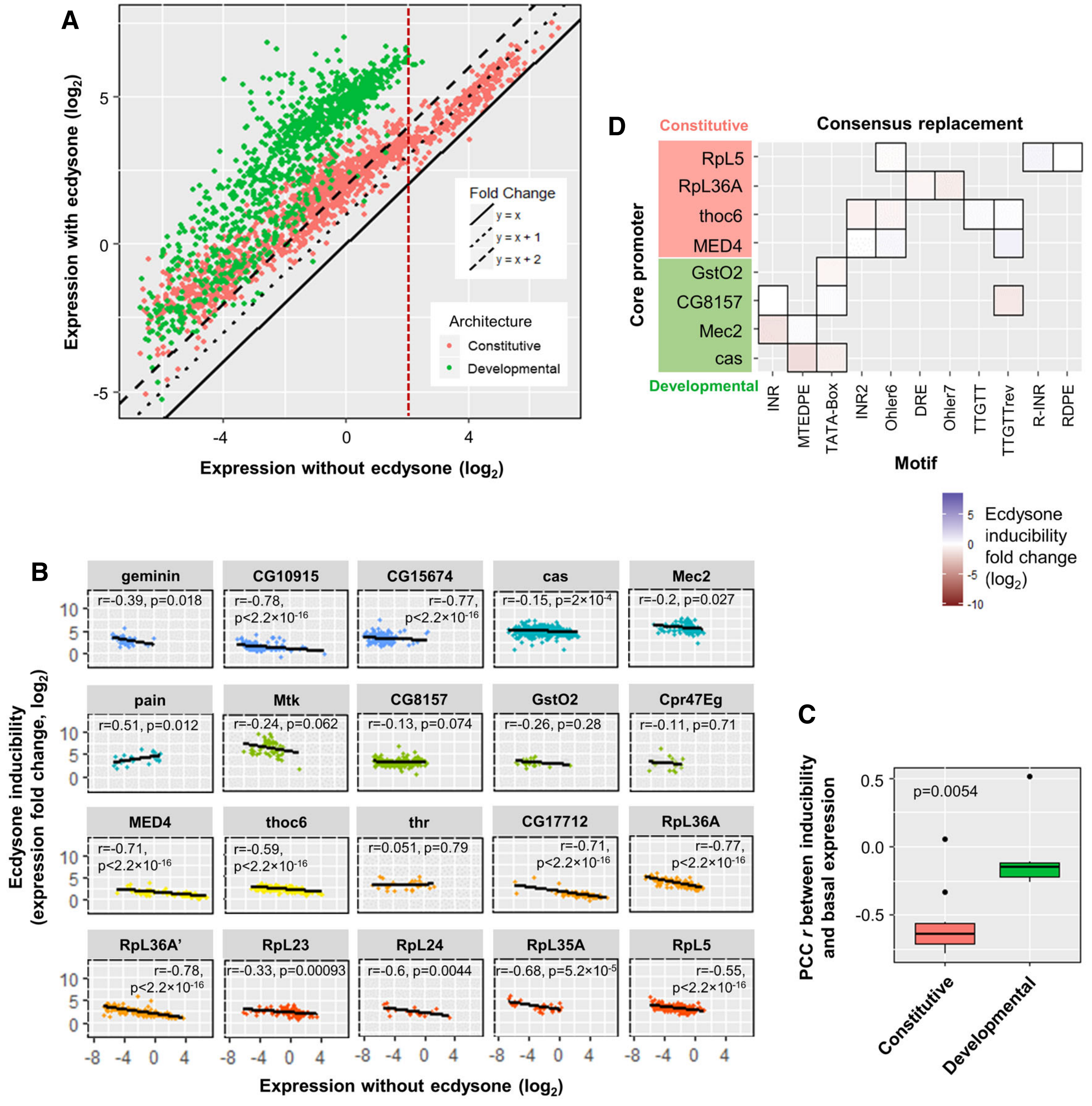

Figure EV5. Ecdysone induction effect (log2 scale) grouped by promoter architectures.

A Scatterplot depicting the expression measurements with ecdysone induction versus measurements without ecdysone for all tested promoters separated by core promoter architectures. Constitutive and developmental promoters are plotted in red and green, respectively. Three types of line are used to indicate the expression fold change with no increase (y = x; solid line), 2-fold increase (y = x + 1; dotted line), and 4-fold increase (y = x + 2; dashed line). Log_2 expressions > 2 on the right of the red dotted line.

B Comparison of the expression fold changes versus measurement values without ecdysone for all native promoters and their mutated versions, grouped by native core promoter sequences. The colors refer to different core promoter architectures. Three types of line are used to indicate the expression fold change with no increase (y = x; solid line), 2-fold increase (y = x + 1; dotted line), and 4-fold increase (y = x + 2; dashed line).

C Comparison of the *PCC rs* obtained in A grouped by constitutive and developmental core promoters. Wilcoxon rank-sum test $P = 0.0054$. The middle hinge represents the median. The interquartile range the difference between the 75th and 25th percentiles. Individual points represent values over 1.5 times the interquartile range. 3–4 biological replicate measurements.

D Heatmap depicting the ecdysone inducibility fold changes caused by consensus replacement of motifs in different core promoters. Constitutive and developmental promoters highlighted in red and green, respectively.

© 2022 The Authors
