# Supplementary material for: Large‐scale analysis of Drosophila core promoter function using synthetic promoters
Source: Mol Syst Biol. 2022 Feb 14;18(2):e9816. doi: 10.15252/msb.20209816 (PMC8842121; doi:10.15252/msb.20209816)
Supplement: Supplementary file 4 [file MSB-18-e9816-s002.pdf]

molecular systems biology

Large-scale analysis of Drosophila core promoter function using synthetic promoters

Zhan Qi, Christophe Jung, Peter Bandilla, Claudia Ludwig, Mark Heron, Anja Kiesel, Mariam Museridze, Julia Philippou-Massier, Miroslav Nikolov, Alessio Renna, Max Schnepf, Ulrich Unnerstall, Stefano Ceolin, Bettina Mühling, Nicolas Gompel, Johannes Söding, and Ulrike Gaul
DOI: 10.15252/msb.20209816

Corresponding author(s): Christophe Jung (jung@genzentrum.lmu.de)

Review Timeline:

Submission Date:	26th Jun 20
Editorial Decision:	14th Aug 20
Revision Received:	25th Aug 21
Editorial Decision:	14th Dec 21
Revision Received:	11th Jan 22
Accepted:	13th Jan 22

Editor: Jingyi Hou

Transaction Report:

(Note: With the exception of the correction of typographical or spelling errors that could be a source of ambiguity, letters and reports are not edited. Depending on transfer agreements, referee reports obtained elsewhere may or may not be included in this compilation. Referee reports are anonymous unless the Referee chooses to sign their reports.)

14th Aug 2020

Manuscript Number: MSB-20-9816
Title: Large-scale analysis of Drosophila core promoter function using synthetic promoters
Author: Zhan Jung
Christophe Jung
Peter Bandilla
Claudia Ludwig
Mark Heron
Anja Kiesel
Julia Philippou-Massier
Miroslav Nikolov
Alessio Renna
Max Schnepf
Ulrich Unnerstall1
Johannes Söding
Ulrike Gaul

Dear Dr Jung,

Thank you for submitting your work to Molecular Systems Biology. We have now heard back from two of the three reviewers who agreed to evaluate your manuscript. Unfortunately, after a series of reminders we did not manage to obtain a report from reviewer #3. In the interest of time, and since the recommendations of the other two reviewers are quite similar, I prefer to make a decision now rather than further delaying the process. As you will see from the reports below, the reviewers acknowledge the potential interest of the study. They raise however a series of concerns, which we would ask you to address in a major revision.

Since the reviewers' recommendations are rather clear, there is no need to reiterate all the points listed below. Some of the key issues that would need to be addressed are the following:

- Attention should be given to placing the findings in the context of existing literature.
- The introduction with regard to the comparison of the presented method to NGS-based methods should be more balanced. The limitations of luciferase-based assay need to be discussed.
- Reviewer #1 is concerned about the in vivo relevance of the presented findings, which we would ask you to address by providing certain levels of validation for at least a couple of examples in an in vivo context.

All other issues raised by the reviewers need to be satisfactorily addressed as well. As you may already know, our editorial policy allows in principle a single round of major revision and it is therefore essential to provide responses to the reviewers' comments that are as complete as possible.

On a more editorial level, we would ask you to address the following issues:

- Please provide a .docx formatted version of the manuscript text (including legends for main figures, EV figures and tables). Please make sure that the changes are highlighted to be clearly visible.

- Please provide individual production quality figure files as .eps, .tif, .jpg (one file per figure).

-Please provide a .docx formatted letter INCLUDING the reviewers' reports and your detailed point-by-point responses to their comments. As part of the EMBO Press transparent editorial process, the point-by-point response is part of the Review Process File (RPF), which will be published alongside your paper.

-Please note that all corresponding authors are required to supply an ORCID ID for their name upon submission of a revised manuscript.

-We replaced Supplementary Information with Expanded View (EV) Figures and Tables that are collapsible/expandable online (see examples in http://msb.embopress.org/content/11/6/812). A maximum of 5 EV Figures can be typeset. EV Figures should be cited as 'Figure EV1, Figure EV2" etc... in the text and their respective legends should be included in the main text after the legends of regular figures.

Additional Tables/Datasets should be labeled and referred to as Table EV1, Dataset EV1, etc. Legends have to be provided in a separate tab in case of .xls files. Alternatively, the legend can be supplied as a separate text file (README) and zipped together with the Table/Dataset file.

For the figures and tables that you do NOT wish to display as Expanded View figures, they should be bundled together with their legends in a single PDF file called *Appendix*, which should start with a short Table of Content. Each legend should be below the corresponding Figure/Table in the Appendix. Appendix figures and tables should be referred to in the main text as: "Appendix Figure S1, Appendix Figure S2, Appendix Table S1" etc. See detailed instructions regarding expanded view here: https://www.embopress.org/page/journal/17444292/authorguide#expandedview.

-Before submitting your revision, primary datasets (and computer code, where appropriate) produced in this study need to be deposited in an appropriate public database (see https://www.embopress.org/page/journal/17444292/authorguide#dataavailability).

Please remember to provide a reviewer password if the datasets are not yet public.

The accession numbers and database should be listed in a formal "Data Availability " section (placed after Materials & Method) that follows the model below (see also https://www.embopress.org/page/journal/17444292/authorguide#dataavailability). Please note that the Data Availability Section is restricted to new primary data that are part of this study.

Data availability

The datasets (and computer code) produced in this study are available in the following databases:

- RNA-Seq data: Gene Expression Omnibus GSE46843 (https://www.ncbi.nlm.nih.gov/geo/query/acc.cgi?acc=GSE46843)
- [data type]: [name of the resource] [accession number/identifier/doi] ([URL or identifiers.org/DATABASE:ACCESSION])

*** Note - All links should resolve to a page where the data can be accessed. ***

- We would encourage you to include the source data for figure panels that show essential quantitative information. Additional information on source data and instruction on how to label the files are available at <
https://www.embopress.org/page/journal/17444292/authorguide#sourcedata
>.

- All Materials and Methods need to be described in the main text. We would encourage you to use 'Structured Methods', our new Materials and Methods format. According to this format, the Material and Methods section should include a Reagents and Tools Table (listing key reagents, experimental models, software and relevant equipment and including their sources and relevant identifiers) followed by a Methods and Protocols section in which we encourage the authors to describe their methods using a step-by-step protocol format with bullet points, to facilitate the adoption of the methodologies across labs. More information on how to adhere to this format as well as downloadable templates (.doc or .xls) for the Reagents and Tools Table can be found in our author guidelines: < https://www.embopress.org/page/journal/17444292/authorguide#researcharticleguide>. An example of a Method paper with Structured Methods can be found here: .

-Regarding data quantification:
Please ensure to specify the name of the statistical test used to generate error bars and P values, the number (n) of independent experiments (please specify technical or biological replicates) underlying each data point and the test used to calculate p-values in each figure legend. Discussion of statistical methodology can be reported in the materials and methods section, but figure legends should contain a basic description of n, P and the test applied.
Graphs must include a description of the bars and the error bars (s.d., s.e.m.).
Please also include scale bars in all microscopy images.

- Please provide a "standfirst text" summarizing the study in one or two sentences (approximately 250 characters, including space), three to four "bullet points" highlighting the main findings and a "synopsis image" (550px width and max 400px height, jpeg format) to highlight the paper on our homepage.
Here are a couple of examples:
https://www.embopress.org/doi/10.15252/msb.20199356
https://www.embopress.org/doi/10.15252/msb.20209475
https://www.embopress.org/doi/10.15252/msb.209495

When you resubmit your manuscript, please download our CHECKLIST (http://bit.ly/EMBOPressAuthorChecklist) and include the completed form in your submission.
Please note that the Author Checklist will be published alongside the paper as part of the transparent process (https://www.embopress.org/page/journal/17444292/authorguide#transparentprocess).

If you feel you can satisfactorily deal with these points and those listed by the referees, you may wish to submit a revised version of your manuscript. Please attach a covering letter giving details of the way in which you have handled each of the points raised by the referees. A revised manuscript will be once again subject to review and you probably understand that we can give you no

guarantee at this stage that the eventual outcome will be favorable.

Yours sincerely,

Jingyi Hou
Editor
Molecular Systems Biology

If you do choose to resubmit, please click on the link below to submit the revision online *within 90 days*.

https://msb.msubmit.net/cgi-bin/main.plex

IMPORTANT: When you send your revision, we will require the following items:
1. the manuscript text in LaTeX, RTF or MS Word format
2. a letter with a detailed description of the changes made in response to the referees. Please specify clearly the exact places in the text (pages and paragraphs) where each change has been made in response to each specific comment given
3. three to four 'bullet points' highlighting the main findings of your study
4. a short 'blurb' text summarizing in two sentences the study (max. 250 characters)
5. a 'thumbnail image' (550px width and max 400px height, Illustrator, PowerPoint or jpeg format), which can be used as 'visual title' for the synopsis section of your paper.
6. Please include an author contributions statement after the Acknowledgements section (see https://www.embopress.org/page/journal/17444292/authorguide)
7. Please complete the CHECKLIST available at (http://bit.ly/EMBOPressAuthorChecklist).
Please note that the Author Checklist will be published alongside the paper as part of the transparent process (https://www.embopress.org/page/journal/17444292/authorguide#transparentprocess).
8. Please note that corresponding authors are required to supply an ORCID ID for their name upon submission of a revised manuscript (EMBO Press signed a joint statement to encourage ORCID adoption).
(https://www.embopress.org/page/journal/17444292/authorguide#editorialprocess)

Currently, our records indicate that there is no ORCID associated with your account.

Please click the link below to provide an ORCID:
Link Not Available

The system will prompt you to fill in your funding and payment information. This will allow Wiley to send you a quote for the article processing charge (APC) in case of acceptance. This quote takes into account any reduction or fee waivers that you may be eligible for. Authors do not need to pay any fees before their manuscript is accepted and transferred to the publisher.

As a matter of course, please make sure that you have correctly followed the instructions for authors as given on the submission website.

*** PLEASE NOTE *** As part of the EMBO Press transparent editorial process initiative (see our Editorial at http://dx.doi.org/10.1038/msb.2010.72), Molecular Systems Biology publishes online a Review Process File with each accepted manuscripts. This file will be published in conjunction with your paper and will include the anonymous referee reports, your point-by-point response and all pertinent correspondence relating to the manuscript. If you do NOT want this File to be published, please inform the editorial office at msb@embo.org within 14 days upon receipt of the present letter.

Reviewer #1:

Review MSB-20-9816

The manuscript by Zhan Qi et al., entitled « Large-scale analysis of Drosophila core promoter function using synthetic promoters » aims at dissecting core promoter function in Drosophila by performing structure-function analysis using synthetic promoter activities. To this end, around 3000 mutational variants, which represent 19 different Drosophila promoter architectures, were

tested using a dual-luciferase reporter assay and robotics. One of the main findings of the manuscript is that a linear combination of motif features accounts for the combinatorial effect on promoter activity.
The study uses an elegant approach, a systematic testing of promoter architectures in cell culture using a standardized pipeline, which allowed them to measure promoter activity at large scale and with high reproducibility. Thus, the study provides a lot of insight into promoter functions, but the main concern is the novelty of the findings and some confirmation of their in cellulo results in the in vivo context. In addition, the manuscript is not always clearly structured, important information is only shown in supplementary figures, while main figures could be moved to the supplement. Thus, the authors need to substantially improve their manuscript before it can be considered for publication in MSB.

Specific comments:

- The authors build their whole study on naturally occurring sequences to construct synthetic promoters; do these synthetic promoters give the same activity in vivo, in the organismal context? do such promoters change their activity in vivo when the findings of this study are applied to them, for example when certain motifs are deleted do the same effects occur in vivo as in cellulo? The cell culture system is a useful screening tool but the situation could be different in the living organism

- p.5, l.106: the authors defined genes (better promoters) based on experimentally genome-wide derived features; could the authors explain why exactly these features were chosen

- the authors state that they designed their approach in a way to test three promoter features, core promoter sequence features (motifs), transcriptional response to external stimulus (Ecdysone) and influence of genomic +/- nucleosomal sequence; while these selections might be obvious, in particular for the motifs, the author should state clearly why they made these selections; for example, why did they use the Ecdysone response element?

- p.5, l.114-115: the authors identified four principal architectures of core promoters; does this mean that these are all the core promoters (based on motif composition) that exist?

- how common are these core promoter architectures when you look into the Drosophila genome?

- It is a bit unusual that the first data of the paper is provided in Fig. 4, all main figures before are just descriptive; I would suggest that most of the data from Fig 1 to 3 should into the Supplement; all Figures should be adapted and connected data should go into one Figure; right now this is a mixture, which makes it hard to read the paper

- the authors state that they designed 3862 synthetic promoter sequences but could test experimentally only about 3000; why is that?

- Figure S1 is not described in the manuscript

- obviously, one of their more important findings was that constitutive core promoters were more sensitive to the influence of nucleosomal sequences downstream of the TSS, but this data is only shown in the supplement; this should be moved to the main figures

- p.7, l.187: why is it expected that the core promoters with no motif show the lowest activity; how common are these promoters? They are located close to which genes? Do they show activity in vivo?

- p.7 l.189-p.8l.198: part of the figure legend from Fig. 4 is pasted in the main text, please correct

- the mutational analysis of motifs is interesting, however my question is what is the relevance in the in vivo context (see above)?

- figure legends are too minimalistic and thus it is sometimes hard to understand what the figures show; for example the blots used to show that combinations of motif knockouts are additive, super- or subadditive (for example in Fig. 4) should be explained more properly

- p. 9,l-256ff: in addition to motif knockout, the authors state that they tested if computationally derived consensus sequences that are preferred in the genome could increase the expression and they found that consensus sequences drove higher expression; this important piece of information is also only shown in the supplement, should however go into the main figures in my opinion; related to this: does this mean that the sequences that the authors tested do not occurred frequently in the genome? How often occur consensus and deviation of the consensus sequences in the genome? Do they have the same effects in vivo?
-
- p.10,l-281: the authors state that they systematically measure the influence on expression under all possible single base pair mutations of the motif consensus for various native promoters, and find that the consensus gave the highest expression; where is this data shown? The authors show expression-based activity logos, some "real" data would be useful

- One major finding is that the activity of any given synthetic core promoter is largely predicted from the linear combination of individual motif features; to test for this, the author could construct a completely artificial promoter and predict, based on their findings, the expression strength and confirm it; in the same line: the authors have the pipeline to test for this, what happens if motifs are randomly mixed? This could answer the question why certain promoter architectures have evolved

- how frequent are the novel motifs, like TTGTTrev in the genome? To test their biological relevance, one

Reviewer #2:

Summary
In this work, the authors set out to functionally characterize Drosophila melanogaster core-promoters and test the necessity and sufficiency of core-promoter motifs to promoter activity. Testing thousands of constructs containing motif deletions, mutations, and positional shifts the authors were able to quantify the contribution of such mutational variants to basal, and ecdysone induced promoter activity. Overall, motif mutations reduced promoter activity and indicated a linear, additive contribution to promoter activity.

The authors present a very large-scale effort in a well-structured and accessible manuscript. Although there are no major insights into core-promoter function or motif importance beyond what is known, the high-quality dataset could be useful for the field. Importantly however, the current version contains several unsubstantiated and incorrect claims and frequently lacks references to prior work and discussions thereof (e.g. regarding similar or contrary findings). We strongly recommend that these shortcomings are corrected in a revised version.

We also recommend including some introduction and discussion of the context in which the various core promoters' function in vivo. For example, for highly regulated genes it makes much sense that basal promoter activities are low or off in the absence of an activator.

Major concerns and recommendations
The authors put forward several incorrect claims about NGS-based methods to measure regulatory element activity, which furthermore are not substantiated by appropriate references or analyses. On page 3, lines 46-47, the authors claim that the "dynamic range of the RNA-seq based MPRAs is usually small (around two orders of magnitude) and they suffer from low accuracy for weak regulatory elements that have a low read coverage", which is incorrect. MPRA studies typically report very highly concordant replicates and low read-coverages correctly indicates weak enhancer or promoter activities. They also have the advantage of assessing transcription more directly at the transcript (i.e. RNA) level (see also below). On the same page, lines 59-61, the authors incorrectly claim that "the measurements of the STAP-seq method were unfortunately not sensitive enough to study the basal activity of the putative promoters in D. mel., but only their enhancer responsiveness". This is incorrect as the publication in question (Arnold 2017) reported both basal and induced core-promoter activities and - most importantly - assessed reporter transcription at single molecule resolution by the use of UMIs.

In addition, technical shortcomings of luciferase assays to assess basal promoter activity are not discussed. Measuring enzymatic activities as a proxy for translation in order to assess transcriptional output in luciferase assays may have confounding variables, such as translation efficiency that likely differs due to the variable 5' UTRs between transcripts arising from the various tested promoters. Additionally, luciferase assays are blind towards the transcription start sites that are actually being used, which is important as they can change between wildtype and mutated core promoters and between basal and ecdysone-induced conditions and may not even lie within the tested promoter fragments (see e.g. Muerdter, F. et al. Nature Methods 2018). While it seems unreasonable to map the transcription starting sites by 5'RACE or similar, this shortcoming needs to be acknowledged and potential implications need to be discussed as they can directly affect the authors' conclusions.

Importantly, having studied core promoters, motifs, and mutational variants that have been described before, the authors fail to reference and discuss earlier work that reached similar but also partly conflicting conclusions. These include but are not limited to
- the relationship between motif quality & position and promoter activity and the ability to predict promoter activity from such sequence features (pages 3, 9-12) have been performed previously in Arnold, et al. Nature Biotech. 2017, which should be noted
- In line 206-208 the authors test mutational variants of the previously described TCT motif without citing Parry et al. Genes & Development 2010. The absence of an effect in mutating the TCT is in contrast to the findings of Parry et al, which should be discussed. The discussion could mention that the differences may arise from transcription originating at another location on the

reporter plasmid or differences in translation efficiency as discussed above. We also recommend staying with the original terminology used to describe the initiator motif in ribosomal protein promoters, i.e. the TCT motif (Parry TJ. et al. Genes & Development). Using a different new terminology (R-INR) may confuse the readers.

- Confirmation of positional motif preferences and co-occurrences on page 11 did not cite similar work which has been previously published (see Ohler U. et al. Genome biology 2002, Ohler W. Nucleic Acid Research 2006 and subsequent work from the Ohler group).
- Functional differences between developmental and constitutive core-promoters have been introduced by Zabidi. et al. Nature 2015, which is not referenced

Minor comments

- Half of Figure 4 legend is misplaced to page 7

Reviewer #1

The manuscript by Zhan Qi et al., entitled « Large-scale analysis of Drosophila core promoter function using synthetic promoters » aims at dissecting core promoter function in Drosophila by performing structure-function analysis using synthetic promoter activities. To this end, around 3000 mutational variants, which represent 19 different Drosophila promoter architectures, were tested using a dual-luciferase reporter assay and robotics. One of the main findings of the manuscript is that a linear combination of motif features accounts for the combinatorial effect on promoter activity.
The study uses an elegant approach, a systematic testing of promoter architectures in cell culture using a standardized pipeline, which allowed them to measure promoter activity at large scale and with high reproducibility. Thus, the study provides a lot of insight into promoter functions, but the main concern is the novelty of the findings and some confirmation of their in cellulo results in the in vivo context.

We thank the reviewer for acknowledging the merits of our method. In our opinion, the novelty lies with the following aspects:

- Technically, the use of purely synthetic promoter sequences to systematically tackle (for the first time to our knowledge) specific questions about the effect of promoter architecture on expression, and the accuracy of our measurement obtained with the Luciferase assay pipeline. As such, we provide the community with comprehensive and accurate measurements of core promoter expression.
- As the reviewer mentioned, we demonstrate that promoter activity can largely be modelled by a linear combination of motif features, which was also never reported before in a study of designed core promoters and with such an amount of data.

We, however, agree that our study would benefit from a validation in a *in vivo* context. We therefore performed additional experiments in living organisms, as described below.

In addition, the manuscript is not always clearly structured, important information is only shown in supplementary figures, while main figures could be moved to the supplement. Thus, the authors need to substantially improve their manuscript before it can be considered for publication in MSB.

We realized that the structure of the manuscript could be improved by swapping different elements between main and supplementary figures. As a consequence, we reorganized the structure of the manuscript by moving the original Fig 2 to Expanded Views and splitting Fig 3 to other main figures in which results are presented. We also changed the former supplementary figure on the effect of nucleosomal sequences on expression to a main panel (the new Fig 2) and expanded its description in the main text. In the revised version, results are presented already in Fig 1 in which we described the new experiments.

Specific comments:

- The authors build their whole study on naturally occurring sequences to construct synthetic promoters; do these synthetic promoters give the same activity in vivo, in the organismal con-

text? do such promoters change their activity in vivo when the findings of this study are applied to them, for example when certain motifs are deleted do the same effects occur in vivo as in cellulo? The cell culture system is a useful screening tool but the situation could be different in the living organism

To directly address this concern, we tested some of our synthetic promoters with reporter constructs in developing *Drosophila* embryos. We created 9 fly lines with different promoters upstream of an optimized reporter system for gene expression quantification that we recently developed (Ceolin et al. 2020, *Commun. Biol*. 3, 663). The tested core promoters included different mutations as well as replacement with motif consensus sequences, for a constitutive (MED4), and a developmental gene (pain). The results, based on triplicate measurements of each embryo, are remarkably consistent with what we observed for the same promoters in cell culture (data shown in the new Fig 1F-I): not only did we validate the similar behaviours *in cellulo and in vivo* by showing a high correlation (Pearson Coefficient of 0.91) between the expression measurements in *S2* cells and in living embryos, but we could also distinguish between different behaviours for the constitutive and the developmental genes selected *in vivo* (more details in the legend of Fig 1F-I and in the main text). We feel that these experiments in an *in vivo* context will constitute an important additional aspect of this study and thank both reviewers for rising this point.

- p.5, l.106: the authors defined genes (better promoters) based on experimentally genome-wide derived features; could the authors explain why exactly these features were chosen

We realized that generally, we described our bioinformatic analysis of the *Drosophila* Core Promoters too briefly in the manuscript. We thus substantially expanded this part as it is also an important contribution to this work:

(1) we now describe the characterization of the four core promoter architectures in much more details (in the Results part on p. 5 l. 124 in the revised version and in the Discussion on p.18 l.564)
(2) to further validate our new sequence motifs, we now present a new analysis of conservation of binding sites for known and the novel motifs among *Drosophila* species (Appendix Fig S1 and column “Conserv.” in Fig EV2).
(3) we show in more details in an additional figure how core promoter motifs correlate with distinct genome-wide derived features (Appendix Fig S2).
(4) we present a new analysis showing that the four promoter architectures have distinct dinucleotide/physical property patterns and nucleosome occupancies (Appendix Fig S3).
(5) we substantially expanded the Material and Methods part on p. 23-25 for the description of the TSS clustering, the selection of the gene sets, the identification of the core promoter elements, and the prediction of gene properties. In particular, the description of the selection of these features asked by the reviewer can be found in Material and Methods on p.24 l. 759.

We hope that these improvements will strengthen the computational analysis of the core promoters.

- the authors state that they designed their approach in a way to test three promoter features, core promoter sequence features (motifs), transcriptional response to external stimulus (Ecdy-

sone) and influence of genomic +/- nucleosomal sequence; while these selections might be obvious, in particular for the motifs, the author should state clearly why they made these selections; for example, why did they use the Ecdysone response element?

We added a sentence in the Introduction on p. 4 l. 117, to explain why the ecdysone pathway is a powerful readout to examine the activities of core promoters. Namely, the steroid hormone ecdysone is important for metamorphosis, molting, and development of the eye and the nerveous system in insects. Its active form (20-hydroxyecdysone) constitutes together with its receptor (the ecdysone receptor EcR) a well-studied activator system for gene expression.
In the revised manuscript, we also better explained the rationale for the promoter features we tested. In particular, we developed the description of the influence of genomic nucleosomal sequence on p.6 l.191-203

- p.5, l.114-115: the authors identified four principal architectures of core promoters; does this mean that these are all the core promoters (based on motif composition) that exist?

We applied our *XXmotif* algorithm (Luehr et al., 2012) for a *genome-wide* motif search in annotated core promoter regions and performed also the subsequent motif composition analysis for all promoters. We then selected for our mutational analysis 19 core promoters representative of the four architectures.

- how common are these core promoter architectures when you look into the Drosophila genome?

The numbers of core promoters for the different architectures are 3076 (Ar 1), 815 (Ar 2), 5170 (Ar 3) and 64 (Ar 4), respectively. We indicated these numbers in the manuscript in the legend of Fig EV3C and in Fig S3 in the Appendix.

- It is a bit unusual that the first data of the paper is provided in Fig. 4, all main figures before are just descriptive; I would suggest that most of the data from Fig 1 to 3 should into the Supplement; all Figures should be adapted and connected data should go into one Figure; right now this is a mixture, which makes it hard to read the paper

We agree with the Reviewer that the first main figures were too descriptive and followed his suggestions: as mentioned above, in the revised figures the first data are presented already in Fig 1, Fig 2 displays only data and the more conceptual figures have been changed into Fig EV4.

- the authors state that they designed 3862 synthetic promoter sequences but could test experimentally only about 3000; why is that?

The lower number of experimentally tested sequences compared to the number of designed sequences is due to the limitation of the synthesis process of the high number of DNA sequences (in our case the manufacturer is Agilent Technologies). During this process, synthesized oligomers are pooled and provided as such. We recovered the individual sequences during the cloning procedure followed by high-throughput sequencing (see Materials and Methods). Se-

quence separation occurs at the colony picking step and the number of picked colonies required increases exponentially with the declining amount of missed sequences. In addition, we observed that about 40% of the recovered sequences presented defects arising from the synthesis procedure, which thus increased the number of picked colonies necessary accordingly.
Hence, it would have become pricely and timely prohibitive to fully recover all the designed sequences, which is why we designed this study and its analysis methods with that in mind. We estimated that picking and preparing glycerol stocks for 4-5 times more colonies than the number of designed sequences leading to recovering 80% sequences constitutes a satisfying compromise without scarifying data quality.
We have now written more details on this point in the manuscript in the Materials and Methods on p32 l.1064.

- Figure S1 is not described in the manuscript

We thank the reviewer for noticing it and added the figure legend.

- obviously, one of their more important findings was that constitutive core promoters were more sensitive to the influence of nucleosomal sequences downstream of the TSS, but this data is only shown in the supplement; this should be moved to the main figures

The reviewer is right that this constitutes an important finding of this work and we moved most of the presentation of these results into the new Fig 2 B-E accordingly. We also expanded the description in the main part of the manuscript by adding on p.9 l.283 a whole paragraph describing in more details this part of the experiments.

- p.7, l.187: why is it expected that the core promoters with no motif show the lowest activity; how common are these promoters? They are located close to which genes? Do they show activity in vivo?

We screened literature for hints whether core promoter with no motif show a particularly low activity, but couldn't find any evidence. We apologize for this undocumented statement that seemed to us to be intuitively obvious, but is not, and we thank the Reviewer for pointing it out. It is however striking that our three motif-less promoters show the lowest activity. As the low number of promoters doesn't allow us to do any statistical analysis, we would like to simply keep an observation in the manuscript. We thus replaced on p. 8 l. 245:

"As expected, the core promoters with no known motif showed the lowest activity."

with

"Strikingly, the core promoters with no known motif showed the lowest activity (in blue in Fig 2A). The low number of selected promoters doesn't however allow for a relevance analysis."

These core promoters with no motifs account a large fraction of the core promoters (about one fourth), as can be seen in the figure bellow in which we display the motif composition for all narrow and broad peak promoters, as determined by our *XXmotif* tool.

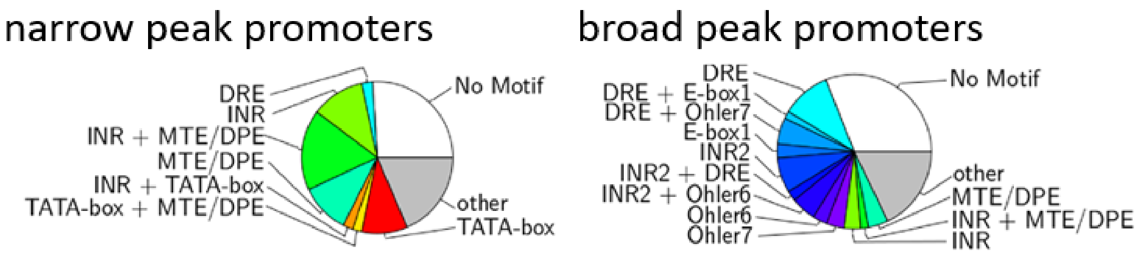

Concerning the location of motif-less promoters, we didn't notice a proximity to particular genes. The motif-less core promoters can however show acivity *in vivo*, as shown in a study (Cannavo et al., 2017 *Nature* 541: 402-406) in which the authors measured gene expression during embryonic development in *Drosophila*. Two out of our three motif-less promoters exhibited low expression during embryogenesis, the third one (geminin) showed moderate expression.

- p.7 l.189-p.8l.198: part of the figure legend from Fig. 4 is pasted in the main text, please correct x

We corrected the mistake.

- the mutational analysis of motifs is interesting, however my question is what is the relevance in the in vivo context (see above)?

As shown above in our new set of validation experiments (new Fig 1F-I), we measured similar promoter activities *in vitro* in living cells and *in vivo* in *Drosophila* embryo. This confirms the relevance of our data in an *in vivo* context.

- figure legends are too minimalistic and thus it is sometimes hard to understand what the figures show; for example the blots used to show that combinations of motif knockouts are additive, super- or subadditive (for example in Fig. 4) should be explained more properly

We apologize for the incompleteness and expanded multiple figure legends.

- p. 9,l-256ff: in addition to motif knockout, the authors state that they tested if computationally derived consensus sequences that are preferred in the genome could increase the expression and they found that consensus sequences drove higher expression; this important piece of information is also only shown in the supplement, should however go into the main figures in my opinion; related to this: does this mean that the sequences that the authors tested do not occurred frequently in the genome? How often occur consensus and deviation of the consensus sequences in the genome? Do they have the same effects in vivo?

We followed the suggestion and moved the figures about consensus replacement as the new Fig 4A-C.

The PWMs of all known and new motifs used in this study are shown in Fig EV2. All motifs sequences are very specific, as shown by the high information content of the logos. As the relative frequency of variations versus consensus decreases with the information content, this indicates that consensus occurs very frequently (although the computationally derived logos tend to be overspecific, as discussed in the manuscript on p. 13 l. 421-438).

One of the constructs tested in in *Drosophila* embryos in the validation experiments described above included the consensus replacement of all motifs for the developmental gene pain. We observe for this construct higher expression than for the *native* version (Fig 1H) similarly to the *in cellulo* measurements. As the expression levels in embryo are consistent with the values we measured with our Luciferase Assay (Fig 1I), one observes the same effects *in cellulo* than *in vivo*.

- p.10,l-281: the authors state that they systematically measure the influence on expression under all possible single base pair mutations of the motif consensus for various native promoters, and find that the consensus gave the highest expression; where is this data shown? The authors show expression-based activity logos, some "real" data would be useful

We show the “real” data in this revised version in Fig 5A, in which the loss of expression after mutation is visualized as dots of different sizes for all measured single point mutations (left panel).

- One major finding is that the activity of any given synthetic core promoter is largely predicted from the linear combination of individual motif features; to test for this, the author could construct a completely artificial promoter and predict, based on their findings, the expression strength and confirm it; in the same line: the authors have the pipeline to test for this, what happens if motifs are randomly mixed? This could answer the question why certain promoter architectures have evolved

To the best of our knowledge, the design of a completely artificial promoter exhibiting experimentally strong activity has not yet been successful. One of the reasons might be the important contribution of the context sequences, as shown herein in Fig 6C-D. Creating such an artificial promoter would thus require extensive experimental testing, which is beyond the scope of this work.
Here, we however tested the combinatorial swap between different architectures of motifs as “blocks” containing their close sequence context information (Fig6E-6F). Each of these constructs (>340 in total) can be considered as a new “artificial” promoter. In this case too, a linear combination largely predicts the experimental expression levels (Fig 6F).

- how frequent are the novel motifs, like TTGTTrev in the genome?

The frequencies of all core promoter motifs detected by XXmotif are indicated in Fig EV2A (column “Occ [%]”) as percentage in which the motif is present in all core promoters. TTGTTrev for example appears in 4.3% of the core promoters, thus relatively frequently.

Reviewer #2:

Major concerns and recommendations
The authors put forward several incorrect claims about NGS-based methods to measure regulatory element activity, which furthermore are not substantiated by appropriate references or analyses. On page 3, lines 46-47, the authors claim that the "dynamic range of the RNA-seq based MPRAs is usually small (around two orders of magnitude) and they suffer from low accuracy for weak regulatory elements that have a low read coverage", which is incorrect. MPRA studies typically report very highly concordant replicates and low read-coverages correctly indicates weak enhancer or promoter activities.

We agree with the Reviewer that MPRAs show highly reproducible measurements. We commented on their relatively small dynamical range based the examination of validation data reported by Arnold et al. 2017. The graph below shows STAP-seq enhancer-responsiveness versus fold change measured by the authors with a Luciferase Reporter Assay. Whereas the Luciferase fold changes continuously cover values ranging from 1 to 643 (a.u.), most of the STAP-seq values are confined ~5 - 200 (a.u.), the lowest 16 values having all 1 as value (red arrow), and the highest value 435 (black arrow). We thus estimated from these data a dynamical range of two orders of magnitude. We didn’t explain this in the text since comparing expression assays is beyond the focus of this work.

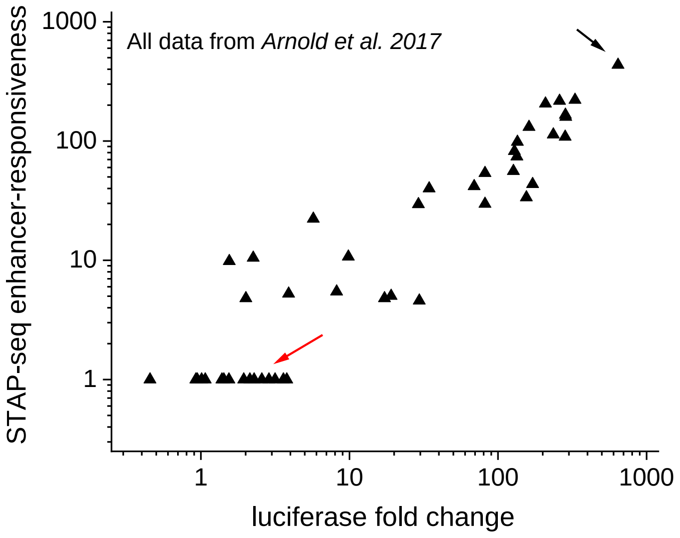

We however acknowledge that MPRAs still allow a relatively large dynamical range, which would be sufficient for most applications. Thus, we toned down our comment by removing the incorrect sentence:
“... dynamic range of the RNA-seq based MPRAs is usually small (around two orders of magnitude) and they suffer from low accuracy for weak regulatory elements that have a low read coverage....”

They also have the advantage of assessing transcription more directly at the transcript (i.e. RNA) level (see also below). On the same page, lines 59-61, the authors incorrectly claim that "the

measurements of the STAP-seq method were unfortunately not sensitive enough to study the basal activity of the putative promoters in D. mel., but only their enhancer responsiveness". This is incorrect as the publication in question (Arnold 2017) reported both basal and induced core-promoter activities and - most importantly - assessed reporter transcription at single molecule resolution by the use of UMIs.

The Reviewer is correct that STAP-seq can measure transcription at single molecule resolution and that they also measured basal core-promoter activities. We meant that the with STAP-seq, the measurement of basal expression may be inaccurate for weak promoters (as can be seen in the figure above). Our formulation was however wrong and we also remove this sentence from the manuscript. We thank the reviewer for noticing the wrong statement.

In addition, technical shortcomings of luciferase assays to assess basal promoter activity are not discussed. Measuring enzymatic activities as a proxy for translation in order to assess transcriptional output in luciferase assays may have confounding variables, such as translation efficiency that likely differs due to the variable 5' UTRs between transcripts arising from the various tested promoters. Additionally, luciferase assays are blind towards the transcription start sites that are actually being used, which is important as they can change between wildtype and mutated core promoters and between basal and ecdysone-induced conditions and may not even lie within the tested promoter fragments (see e.g. Muerdter, F. et al. Nature Methods 2018). While it seems unreasonable to map the transcription starting sites by 5'RACE or similar, this shortcoming needs to be acknowledged and potential implications need to be discussed as they can directly affect the authors' conclusions.

These are indeed limitations of the Luciferase Assays. We now explicitly referred to them together with other shortcomings of our method in the Discussion by adding on p 21 l. 691:

“Our method based on the Luciferase assay for assessing promoter activity has however limitations: different translation efficiency due to the varying 5’UTR between transcripts are not captured, and the assay is blind toward the TSS that is actually being used in the endogenous promoters. These phenomena could lead to promoter activity measurements that don’t perfectly reflect the endogenous expression. Additional shortcomings of our technique are that the measurements were performed using episomal plasmids in transiently transfected cells. Although we inserted the genomic ±1 nucleosome positioning sequences from different genes surrounding the tested core promoter region, they still lack the ability to represent the endogenous chromosomal context and the higher-order genomic structure, which might change the basal expression levels as well as the ecdysone inducibilities. Furthermore, our method has a moderate throughput that is lower than most sequencing-based approaches, and the cloning and colony picking procedures also limit our sequence recovery from the designed oligonucleotides (Materials and Methods). “

We thank the Reviewer for pointing out these shortcomings.

Importantly, having studied core promoters, motifs, and mutational variants that have been described before, the authors fail to reference and discuss earlier work that reached similar but also partly conflicting conclusions. These include but are not limited to

- the relationship between motif quality & position and promoter activity and the ability to predict promoter activity from such sequence features (pages 3, 9-12) have been performed previously in Arnold, et al. Nature Biotech. 2017, which should be noted

We now discuss the findings of Arnold et al. in the Introduction on p.3. l. 68 by adding:

" ... Noteworthy, Arnold et al. (Arnold et al., 2017) showed for the main motifs Inr, TATA and DPE that the resemblance with the canonical sequences correlates with the responsiveness of the enhancer targeting the promoter (i.e., how much expression changes when an enhancer is active), with an increasing responsiveness observed for higher position weight matrix (PWM) match scores. Interestingly, they also found that the correlation is higher for strongly responsive sequences than for weaker ones......"

And in the Discussion on p. 19 l. 631:

"... Altering motif positions overall decreases expression. This phenomenon has been observed before by Schor et al., who showed using CAGE measurements that changing the distance between motifs could have a major impact on transcriptional initiation and overall transcripts levels (Schor et al., 2017). More generally, Arnold et al. (Arnold et al., 2017) demonstrated that the positional occurrence of specific 5-mers relative to the TSS is predictive of the enhancer sequences' responsiveness by a linear model, which is difficult to validate with our measurements due to too few data points for positioning compared to a deep sequencing method."

- In line 206-208 the authors test mutational variants of the previously described TCT motif without citing Parry et al. Genes & Development 2010. The absence of an effect in mutating the TCT is in contrast to the findings of Parry et al, which should be discussed. The discussion could mention that the differences may arise from transcription originating at another location on the reporter plasmid or differences in translation efficiency as discussed above. We also recommend staying with the original terminology used to describe the initiator motif in ribosomal protein promoters, i.e. the TCT motif (Parry TJ. et al. Genes & Development). Using a different new terminology (R-INR) may confuse the readers.

We originally renamed the TCT motif the Ribosomal Initiator (R-INR) in our work based on our bioinformatic study that showed a strong enrichment in the ribosomal genes. We however understand that this could be misleading for the reader. We thus followed the reviewer's recommendation and replaced R-INR with the former terminology TCT along the whole manuscript.

We also discuss the contrast with Parry et al.'s findings in the Discussion on p. 18 l. 596:

".... However, not all well-characterized motifs have a significant effect on expression in our measurements. This is especially the case with TCT. This stands in contrast with the strong loss of transcriptional activity observed by Parry et al. in their mutational analysis (Parry et al., 2010). The differences may arise from transcription originating at another location on the reporter plasmid or differences in translation efficiency, as discussed above..."

An on p. 17 l. 583:

“...As shown by Parry et al. (Parry et al., 2010), genes containing the TCT are not regulated by TFIID, but by a special RNA polymerase II system for ribosomal protein genes....”

- Confirmation of positional motif preferences and co-occurrences on page 11 did not cite similar work which has been previously published (see Ohler U. et al. Genome biology 2002, Ohler W. Nucleic Acid Research 2006 and subsequent work from the Ohler group).

We apologize for the oversight and added the references in the manuscript on p. 13 l. 441:

“The XXmotif analysis showed the strong positional preferences of some motifs (Appendix Table S1), as already reported by Ohler et al. (Ohler, 2006; Ohler et al., 2002; Rach et al., 2009)....”

- Functional differences between developmental and constitutive core-promoters have been introduced by Zabidi. et al. Nature 2015, which is not referenced

We added the missing reference on p. 6 l. 177.

Minor comments

- Half of Figure 4 legend is misplaced to page 7

We thank the reviewer for noticing the mistake and corrected it.

14th Dec 2021

Manuscript Number: MSB-20-9816R
Title: Large-scale analysis of Drosophila core promoter function using synthetic promoters
Author: Christophe Jung
Peter Bandilla
Claudia Ludwig
Mark Heron
Anja Kiesel
Julia Philippou-Massier
Miroslav Nikolov
Alessio Renna
Max Schnepf
Ulrich Unnerstall1
Johannes Söding
Ulrike Gaul
Mariam Museridze
Stefano Ceolin
Bettina Mühling
Nicolas Gompel
Zhan Qi

Thank you for sending us your revised manuscript. We have now heard back from the two reviewers who were asked to evaluate your study. As you will see, the reviewers are satisfied with the modifications made and think that the study is now suitable for publication.

Before we can formally accept your manuscript, we would ask you to address the following issues:

1. The remaining minor issues raised by Reviewer #2.

On a more editorial level :

2. Please reduce the keyword number to five.

3. Author contribution: Miroslav Nikolov is missing. Please fix it.

4. Please update the email address for Max Schnepf when you submit the revised manuscript.

5. Please rename "DECLARATION OF INTERESTS" to "Conflicts of interest".

6. FIGURE CALLOUTS: Fig 1C,D are missing; Fig 1f-I are called out after Fig 2A; Appendix Figure S3 and Appendix Table S6 are not called out. Please fix these issues. Please check the figure callouts in the main article and make sure that all Figures and Tables are called for and are in the order that represents the order of the figure display.

7. FUNDING:
- Please merge it with Acknowledgements.
- Center for Integrated Protein Science (CIPSM), DFG, German Research Foundation) - Project-ID 213249687 -, and the Humboldt- Foundation (Alexander von Humboldt-Professorship) are not in the online submission system. Please make sure the funding information in the manuscript file matches those entered in the submission system.

8. Please remove "Data not shown". Please note that our editorial policy does not allow "data not shown". All data referred to in the paper should be displayed in the main or Expanded View figures.

9. We would also encourage you to include the source data for figure panels that show essential data. Numerical data should be provided as individual .xls or .csv files (including a tab describing the data). Additional information on source data and instruction on how to label the files are available at https://www.embopress.org/page/journal/17444292/authorguide#sourcedata

10. Our journal encourages the inclusion of *data citations in the reference list* to directly cite datasets that were re-used and obtained from public databases. Data citations in the article text are distinct from normal bibliographical citations and should

directly link to the database records from which the data can be accessed. In the main text, data citations are formatted as follows: "Data ref: Smith et al, 2001". In the Reference list, data citations must be labeled with "[DATASET]". A data reference must provide the database name, accession number/identifiers and a resolvable link to the landing page from which the data can be accessed at the end of the reference. Further instructions are available at .

11. Please upload EV figures as separate files and provide individual production quality figure files as .eps, .tif, .jpg (one file per figure).

12. Appendix file:
- add a Table of Content on the 1st page.
-"Additional text" seems to be a method description. Please move it to the main manuscript text and merge it with Materials and Methods.

13. Data availability:
- you wrote, "The DNA sequences and their raw expression measurements are provided for all tested sequences in Supplementary File". Please specify the file names, as we do not have "Supplementary file".
- Next-generation sequencing data and computer code need to be deposited in an appropriate public database (see https://www.embopress.org/page/journal/17444292/authorguide#dataavailability).

Data Availability
- [data type]: [name of the resource] [accession number/identifier/doi] ([URL or identifiers.org/DATABASE:ACCESSION])

*** Note - All links should resolve to a page where the data can be accessed. ***

14. Please provide a "standfirst text" summarizing the study in one or two sentences (approximately 250 characters, including space), three to four "bullet points" highlighting the main findings and a "synopsis image" (550px width and 400-600px height, PNG format) to highlight the paper on our homepage.

Here are a couple of examples:
https://www.embopress.org/doi/10.15252/msb.20199356
https://www.embopress.org/doi/10.15252/msb.20209475
https://www.embopress.org/doi/10.15252/msb.209495

15. Our data editors have seen the manuscript, and they have made some comments and suggestions that need to be addressed (see attached). Please send back a revised version (in track change mode), as we will need to go through the changes.

When you resubmit your manuscript, please download our CHECKLIST (https://bit.ly/EMBOPressAuthorChecklist) and include the completed form in your submission. *Please note* that the Author Checklist will be published alongside the paper as part of the transparent process (https://www.embopress.org/page/journal/17444292/authorguide#transparentprocess)

Click on the link below to submit your revised paper.

https://msb.msubmit.net/cgi-bin/main.plex

As a matter of course, please make sure that you have correctly followed the instructions for authors as given on the submission website.

I look forward to receiving your revised manuscript as soon as possible.

Kind regards,
Jingyi

Jingyi Hou
Editor
Molecular Systems Biology

If you do choose to resubmit, please click on the link below to submit the revision online before 13th Jan 2022.

https://msb.msubmit.net/cgi-bin/main.plex

IMPORTANT: When you send your revision, we will require the following items:
1. the manuscript text in LaTeX, RTF or MS Word format
2. a letter with a detailed description of the changes made in response to the referees. Please specify clearly the exact places in the text (pages and paragraphs) where each change has been made in response to each specific comment given
3. three to four 'bullet points' highlighting the main findings of your study
4. a short 'blurb' text summarizing in two sentences the study (max. 250 characters)
5. a 'thumbnail image' (550px width and max 400px height, Illustrator, PowerPoint or jpeg format), which can be used as 'visual title' for the synopsis section of your paper.
6. Please include an author contributions statement after the Acknowledgements section (see https://www.embopress.org/page/journal/17444292/authorguide#manuscriptpreparation)
7. Please complete the CHECKLIST available at (https://bit.ly/EMBOPressAuthorChecklist).
Please note that the Author Checklist will be published alongside the paper as part of the transparent process (https://www.embopress.org/page/journal/17444292/authorguide#transparentprocess).
8. Please note that corresponding authors are required to supply an ORCID ID for their name upon submission of a revised manuscript (EMBO Press signed a joint statement to encourage ORCID adoption) (https://www.embopress.org/page/journal/17444292/authorguide#editorialprocess).
9. When assembling figures, please refer to our figure preparation guideline in order to ensure proper formatting and readability in print as well as on screen:
https://bit.ly/EMBOPressFigurePreparationGuideline
See also figure legend guidelines: https://www.embopress.org/page/journal/17444292/authorguide#figureformat

Currently, our records indicate that the ORCID for your account is 0000-0002-9774-1125.

Please click the link below to modify this ORCID:
Link Not Available

The system will prompt you to fill in your funding and payment information. This will allow Wiley to send you a quote for the article processing charge (APC) in case of acceptance. This quote takes into account any reduction or fee waivers that you may be eligible for. Authors do not need to pay any fees before their manuscript is accepted and transferred to the publisher.

As a matter of course, please make sure that you have correctly followed the instructions for authors as given on the submission website.

*** PLEASE NOTE *** As part of the EMBO Press transparent editorial process initiative (see our Editorial at https://dx.doi.org/10.1038/msb.2010.72 , Molecular Systems Biology will publish online a Review Process File to accompany accepted manuscripts. When preparing your letter of response, please be aware that in the event of acceptance, your cover letter/point-by-point document will be included as part of this File, which will be available to the scientific community. More information about this initiative is available in our Instructions to Authors. If you have any questions about this initiative, please contact the editorial office (msb@embo.org).

Reviewer #1:

I went very carefully though all reviewer comments and found all them addressed in a satisfactory manner. The manuscript has improved substantially, I now recommend publication.

Reviewer #2:

The authors have substantially revised their manuscript and addressed all our comments. We support publication once two minor comments regarding the revised figures are addressed.

Figure 1 panel F:
The labels inside the embryo images make it hard to see the fluorescent signal of the reporters. The labels also don't indicate the type of mutation or motif KO that are mentioned in the figure legend. Please clarify.

Figure 4: To interpret the result of motifs replacement and rescue it would be good to know if the motifs are sufficient to drive transcription when placed in a random background. Please add this information if available or else note this as a caveat (no experiment required!).

Reviewer #2:
Figure 1 panel F:
The labels inside the embryo images make it hard to see the fluorescent signal of the reporters. The labels also don't indicate the type of mutation or motif KO that are mentioned in the figure legend. Please clarify.

We clarified the Figure by moving the labels on the outside of the embryos and specifying more precisely the type of mutations in the figure as well as in the figure caption.

Figure 4: To interpret the result of motifs replacement and rescue it would be good to know if the motifs are sufficient to drive transcription when placed in a random background. Please add this information if available or else note this as a caveat (no experiment required!).

The deeper investigation of the influence of the sequence context was beyond the scope of this study, and we therefore didn’t test motif activities in random context sequences. We added this information on p.19 in the Discussion:
“We didn’t test motif activity in random sequence context. We nevertheless see an influence of the sequence context independent of the motifs, which may obey complicated rules. It is however beyond the scope of this study.”

13th Jan 2022
Manuscript number: MSB-20-9816RR
Title: Large-scale analysis of Drosophila core promoter function using synthetic promoters

Dear Dr. Jung,

Thank you again for sending us your revised manuscript. We are now satisfied with the modifications made and I am pleased to inform you that your paper has been accepted for publication.

*** PLEASE NOTE *** As part of the EMBO Publications transparent editorial process initiative (see our Editorial at https://dx.doi.org/10.1038/msb.2010.72), Molecular Systems Biology publishes online a Review Process File with each accepted manuscripts. This file will be published in conjunction with your paper and will include the anonymous referee reports, your point- by-point response and all pertinent correspondence relating to the manuscript. If you do NOT want this File to be published, please inform the editorial office at msb@embo.org within 14 days upon receipt of the present letter.

Should you be planning a Press Release on your article, please get in contact with msb@wiley.com as early as possible, in order to coordinate publication and release dates.

LICENSE AND PAYMENT:
All articles published in Molecular Systems Biology are fully open access: immediately and freely available to read, download and share.

Molecular Systems Biology charges an article processing charge (APC) to cover the publication costs. You, as the corresponding author for this manuscript, should have already received a quote with the article processing fee separately. Please let us know in case this quote has not been received.

Once your article is at Wiley for editorial production you will receive an email from Wiley's Author Services system, which will ask you to log in and will present you with the publication license form for completion. Within the same system the publication fee can be paid by credit card, an invoice or pro forma can be requested.

Payment of the publication charge and the signed Open Access Agreement form must be received before the article can be published online.

Molecular Systems Biology articles are published under the Creative Commons licence CC BY, which facilitates the sharing of scientific information by reducing legal barriers, while mandating attribution of the source in accordance to standard scholarly practice.

Proofs will be forwarded to you within the next 2-3 weeks.

Thank you very much for submitting your work to Molecular Systems Biology.

Sincerely,
Jingyi

Jingyi Hou
Editor
Molecular Systems Biology

EMBO PRESS

YOU MUST COMPLETE ALL CELLS WITH A PINK BACKGROUND

PLEASE NOTE THAT THIS CHECKLIST WILL BE PUBLISHED ALONGSIDE YOUR PAPER

Corresponding Author Name: Christophe Jung
Journal Submitted to: Molecular Systems Biology
Manuscript Number: MSB-20-9816

USEFUL LINKS FOR COMPLETING THIS FORM

http://www.antibodypedia.com
http://1degreebio.org
http://www.equator-network.org/reporting-guidelines/improving-bioscience-research-repor
http://grants.nih.gov/grants/olaw/olaw.htm
http://www.mrc.ac.uk/Ourresearch/Ethicsresearchguidance/Useofanimals/index.htm
http://ClinicalTrials.gov
http://www.consort-statement.org
http://www.consort-statement.org/checklists/view/32-consort/66-title
http://www.equator-network.org/reporting-guidelines/reporting-recommendations-for-tumo
http://datadryad.org
http://figshare.com
http://www.ncbi.nlm.nih.gov/gap
http://www.ebi.ac.uk/ega
http://biomodels.net/
http://biomodels.net/miriam/
http://jjj.biochem.sun.ac.za
https://osp.od.nih.gov/biosafety-biosecurity-and-emerging-biotechnology/
http://www.selectagents.gov/

Reporting Checklist For Life Sciences Articles (Rev. June 2017)

This checklist is used to ensure good reporting standards and to improve the reproducibility of published results. These guidelines are consistent with the Principles and Guidelines for Reporting Preclinical Research issued by the NIH in 2014. Please follow the journal's authorship guidelines in preparing your manuscript.

A- Figures

1. Data

The data shown in figures should satisfy the following conditions:

- the data were obtained and processed according to the field's best practice and are presented to reflect the results of the experiments in an accurate and unbiased manner.
- figure panels include only data points, measurements or observations that can be compared to each other in a scientifically meaningful way.
- graphs include clearly labeled error bars for independent experiments and sample sizes. Unless justified, error bars should not be shown for technical replicates.
- if n< 5, the individual data points from each experiment should be plotted and any statistical test employed should be justified
- Source Data should be included to report the data underlying graphs. Please follow the guidelines set out in the author ship guidelines on Data Presentation.

2. Captions

Each figure caption should contain the following information, for each panel where they are relevant:

- a specification of the experimental system investigated (eg cell line, species name).
- the assay(s) and method(s) used to carry out the reported observations and measurements
- an explicit mention of the biological and chemical entity(ies) that are being measured.
- an explicit mention of the biological and chemical entity(ies) that are altered/varied/perturbed in a controlled manner.

- the exact sample size (n) for each experimental group/condition, given as a number, not a range;
- a description of the sample collection allowing the reader to understand whether the samples represent technical or biological replicates (including how many animals, litters, cultures, etc.).
- a statement of how many times the experiment shown was independently replicated in the laboratory.
- definitions of statistical methods and measures:
  - common tests, such as t-test (please specify whether paired vs. unpaired), simple χ2 tests, Wilcoxon and Mann-Whitney tests, can be unambiguously identified by name only, but more complex techniques should be described in the methods section;
  - are tests one-sided or two-sided?
  - are there adjustments for multiple comparisons?
  - exact statistical test results, e.g., P values = x but not P values < x;
  - definition of 'center values' as median or average;
  - definition of error bars as s.d. or s.e.m.

Any descriptions too long for the figure legend should be included in the methods section and/or with the source data.

In the pink boxes below, please ensure that the answers to the following questions are reported in the manuscript itself. Every question should be answered. If the question is not relevant to your research, please write NA (non applicable). We encourage you to include a specific subsection in the methods section for statistics, reagents, animal models and human subjects.

B- Statistics and general methods

Please fill out these boxes (Do not worry if you cannot see all your text once you press return)

1.a. How was the sample size chosen to ensure adequate power to detect a pre-specified effect size?	Thousands of different promoter sequences were individually measured. For each synthetic Core Promoter sequence, we measure at least 3-4 biological replicates, th emasurements beeing conducted on different days for the different replicates.
1.b. For animal studies, include a statement about sample size estimate even if no statistical methods were used.	For every enhancer-reporter construct in this study, three or more biological replicates were measured. For the in cellulo assay, outliers were excluded using a score defined in the section "Oulier identification and filtering" of Materials and Methods
2. Describe inclusion/exclusion criteria if samples or animals were excluded from the analysis. Were the criteria pre-established?	We discarded only movies affected by technical issues, e.g. in which the sample moved during the experiment, making the analysis more difficult or not possible. After this exclusion, we could always analyze three independent biological replicates for each enhancer-reporter construct.
3. Were any steps taken to minimize the effects of subjective bias when allocating animals/samples to treatment (e.g. randomization procedure)? If yes, please describe.	Such steps were not taken.
For animal studies, include a statement about randomization even if no randomization was used.	Organisms have been grouped based on the carried enhancer-reporter construct. Embryos to be imaged were chosen randomly among embryos at the proper stage of developpment (stage 5).
4.a. Were any steps taken to minimize the effects of subjective bias during group allocation or/and when assessing results (e.g. blinding of the investigator)? If yes please describe.	Such steps were not taken.
4.b. For animal studies, include a statement about blinding even if no blinding was done	Organisms have been grouped based on the carried enhancer-reporter construct and selected randomly. Blinding was not relevant in this study.
5. For every figure, are statistical tests justified as appropriate?	We performed for every figure including comparion of samples a Wilcoxon rank-sum test since this non-parametric test has a great statistical power to compare independant sample measurement sets.
Do the data meet the assumptions of the tests (e.g., normal distribution)? Describe any methods used to assess it.	The Wilcoxon rank-sum test does not require to assume normal distribution.
Is there an estimate of variation within each group of data?	We used the median standard deviation as an estimate of variation for the expression levels (mean variation for all our data ~21%).

Is the variance similar between the groups that are being statistically compared?	Yes

C- Reagents

6. To show that antibodies were profiled for use in the system under study (assay and species), provide a citation, catalog number and/or clone number, supplementary information or reference to an antibody validation profile. e.g., Antibodypedia (see link list at top right), 1DegreeBio (see link list at top right).	No antibody staining was used in this study.
7. Identify the source of cell lines and report if they were recently authenticated (e.g., by STR profiling) and tested for mycoplasma contamination.	Drosophila Schneider 2 (S2) cells were obtained from the laboratory stock of Prof. Klaus Förstemann (gene Center, Munich). They were tested for mycoplasma contamination.

* for all hyperlinks, please see the table at the top right of the document

D- Animal Models

8. Report species, strain, gender, age of animals and genetic modification status where applicable. Please detail housing and husbandry conditions and the source of animals.	Transgenic Drosophila Melanogaster fly stocks were generated by integrating reporter constructs in the same attP2 docking site using PhiC31 integrase.
9. For experiments involving live vertebrates, include a statement of compliance with ethical regulations and identify the committee(s) approving the experiments.	No ethical approval was required for this study.
10. We recommend consulting the ARRIVE guidelines (see link list at top right) (PLoS Biol. 8(6), e1000412, 2010) to ensure that other relevant aspects of animal studies are adequately reported. See author guidelines, under 'Reporting Guidelines'. See also: NIH (see link list at top right) and MRC (see link list at top right) recommendations. Please confirm compliance.	We confirm compliance.

E- Human Subjects

11. Identify the committee(s) approving the study protocol.	n.a.
12. Include a statement confirming that informed consent was obtained from all subjects and that the experiments conformed to the principles set out in the WMA Declaration of Helsinki and the Department of Health and Human Services Belmont Report.	n.a.
13. For publication of patient photos, include a statement confirming that consent to publish was obtained.	n.a.
14. Report any restrictions on the availability (and/or on the use) of human data or samples.	n.a.
15. Report the clinical trial registration number (at ClinicalTrials.gov or equivalent), where applicable.	n.a.
16. For phase II and III randomized controlled trials, please refer to the CONSORT flow diagram (see link list at top right) and submit the CONSORT checklist (see link list at top right) with your submission. See author guidelines, under 'Reporting Guidelines'. Please confirm you have submitted this list.	n.a.
17. For tumor marker prognostic studies, we recommend that you follow the REMARK reporting guidelines (see link list at top right). See author guidelines, under 'Reporting Guidelines'. Please confirm you have followed these guidelines.	n.a.

F- Data Accessibility

18: Provide a "Data Availability" section at the end of the Materials & Methods, listing the accession codes for data generated in this study and deposited in a public database (e.g. RNA-Seq data: Gene Expression Omnibus GSE39462, Proteomics data: PRIDE PXD000208 etc.) Please refer to our author guidelines for 'Data Deposition'.<br><br>Data deposition in a public repository is mandatory for:<br>a. Protein, DNA and RNA sequences<br>b. Macromolecular structures<br>c. Crystallographic data for small molecules<br>d. Functional genomics data<br>e. Proteomics and molecular interactions	We provide a "Data Availibility" statement at the end of the Materials and Methods. No publicity datasets were used. All figures have associated raw data and there is no restriction about data availability.
19. Deposition is strongly recommended for any datasets that are central and integral to the study; please consider the journal's data policy. If no structured public repository exists for a given data type, we encourage the provision of datasets in the manuscript as a Supplementary Document (see author guidelines under 'Expanded View' or in unstructured repositories such as Dryad (see link list at top right) or Figshare (see link list at top right).	The datasets of the expression levels for all promoters are available as an Excel file.
20. Access to human clinical and genomic datasets should be provided with as few restrictions as possible while respecting ethical obligations to the patients and relevant medical and legal issues. If practically possible and compatible with the individual consent agreement used in the study, such data should be deposited in one of the major public access-controlled repositories such as dbGAP (see link list at top right) or EGA (see link list at top right).	n.a.
21. Computational models that are central and integral to a study should be shared without restrictions and provided in a machine-readable form. The relevant accession numbers or links should be provided. When possible, standardized format (SBML, CellML) should be used instead of scripts (e.g. MATLAB). Authors are strongly encouraged to follow the MIRIAM guidelines (see link list at top right) and deposit their model in a public database such as Biomodels (see link list at top right) or JWS Online (see link list at top right). If computer source code is provided with the paper, it should be deposited in a public repository or included in supplementary information.	We provide a "Data Availibility" statement at the end of the Materials and Methods. No publicity datasets were used. All figures have associated raw data and there is no restriction about data availability.

G- Dual use research of concern

22. Could your study fall under dual use research restrictions? Please check biosecurity documents (see link list at top right) and list of select agents and toxins (APHIS/CDC) (see link list at top right). According to our biosecurity guidelines, provide a statement only if it could.	n.a.
